# From Single-Modal to Multi-Modal Artificial Intelligence in Alzheimer’s Disease: A Systematic Review of Databases, Modalities, Diagnostic Performance, and Clinical Translation Challenges

**DOI:** 10.3390/s26175489

**Published:** 2026-08-29

**Authors:** José Menezes, Maria Inês Barbosa, Pedro Miguel Rodrigues

**Affiliations:** CBQF—Centro de Biotecnologia e Química Fina, Escola Superior de Biotecnologia, Portuguesa, Universidade Católica Portuguesa, 4169-005 Porto, Portugal; s-jmmenezes@ucp.pt (J.M.); mibarbosa@ucp.pt (M.I.B.)

**Keywords:** Alzheimer’s disease, multi-modal artificial intelligence, machine learning, deep learning, data fusion, neuroimaging, biomarkers, early diagnosis

## Abstract

Alzheimer’s disease (AD) is the leading cause of dementia and a major cause of death worldwide, making early detection a critical clinical priority. Because pathological changes may begin 15–20 years before symptom onset, artificial intelligence (AI) has emerged as a promising tool for identifying and characterizing AD. In particular, multi-modal approaches that integrate cognitive, biological, and sensor-based data have attracted growing interest. This systematic review compares single- and multi-modal AI strategies for AD detection, covering machine learning and deep learning methods, feature representations, validation strategies, and classification tasks. Searches of major databases identified 568 studies published between 2016 and early 2026; 278 met the inclusion criteria according to PRISMA guidelines. Multi-modal approaches generally achieved higher performance than single-modal strategies, particularly for challenging tasks such as predicting progression between closely related disease stages, although direct comparisons under identical conditions remain scarce. Critically, only about 4% of studies evaluated their models on a genuinely independent external cohort, raising substantial concerns about model generalizability. Overall, current AI systems remain highly dependent on existing datasets and heterogeneous evaluation protocols, which limit generalizability and clinical applicability. Future research should prioritize representative multimodal datasets, rigorous external validation, and clinically interpretable AI systems.

## 1. Introduction

Alzheimer’s disease (AD) is the leading cause of dementia, accounting for 60–80% of cases and ranking as the seventh leading cause of mortality worldwide [1]. First described by Alois Alzheimer in 1906 [2], AD is characterised by a slow and insidious progression that leads to irreversible neurodegeneration [3], widespread brain atrophy and the accumulation of pathological protein aggregates, namely amyloid-β (Aβ) plaques and hyperphosphorylated tau tangles [4,5]. These neuropathological changes manifest as progressive memory loss, cognitive impairment and difficulties with visuospatial processing [1], ultimately resulting in loss of independence and complete dependence on caregivers [6].

The diagnosis of AD relies on a combination of clinical and paraclinical approaches. Neuropsychological assessments form the basis of clinical evaluation and commonly include tools such the Mini-Mental State Examination (MMSE), the Montreal Cognitive Assessment (MoCA) and the Clinical Dementia Rating (CDR), which quantify cognitive domains including memory, attention, reasoning and executive function [7,8,9]. These assessments are complemented by laboratory-based biomarkers, particularly cerebrospinal fluid (CSF) measures of Aβ and tau proteins, which provide insight into the underlying neuropathology and can improve diagnostic accuracy [10,11]. Neuroimaging also plays a central role in AD evaluation, with magnetic resonance imaging (MRI) and positron emission tomography (PET) enabling the visualisation of structural, metabolic and molecular brain alterations associated with disease progression [12].

Despite the range of clinical and paraclinical tools available, a persistent gap remains between how clinicians diagnose AD in practice and how AI systems are trained to detect it. Clinical diagnosis is an integrative and longitudinal process in which physicians synthesize information from multiple sources, including neuropsychological performance, fluid and imaging biomarkers, medical history, symptom progression, and the frequent presence of mixed pathologies and comorbidities [3,13]. AI models, by contrast, are typically optimized to identify statistical patterns associated with predefined diagnostic categories, often aiming to distinguish discrete diagnostic classes using cross-sectional data from curated research cohorts [14,15]. As a result, models may achieve high accuracy on benchmark datasets while capturing only a limited portion of the complexity, uncertainty, and heterogeneity that characterize real-world diagnostic practice [16,17]. Bridging this gap, rather than incrementally improving classification accuracy alone, is essential for translating AI into clinically useful decision-support tools [18]. This perspective motivated the present review’s emphasis on dataset composition, methodological quality, model evaluation, and clinical applicability alongside raw predictive performance.

Despite advances in diagnostic techniques and biological understanding, the global burden of AD continues to increase. According to the Global Burden of Disease study, approximately 57.4 million individuals were living with dementia in 2019, with projections indicating an increase to 152.8 million by 2050 [19,20]. This rise is largely driven by population growth and demographic aging [20]. Moreover, up to 45% of dementia cases are attributed to modifiable risk factors such as hypertension, obesity, diabetes, physical inactivity, smoking, depression and low educational attainment, suggesting that prevention and early intervention strategies could substantially reduce disease burden [6].

A defining feature of AD is its long preclinical phase, during which pathological processes may begin one to two decades before clinical symptom onset [21,22]. Longitudinal studies of both autosomal-dominant and sporadic AD have helped characterise the temporal sequence of these events relative to the onset of symptoms. CSF Aβ42 abnormalities may emerge 20–25 years before symptoms appear, while fibrillar Aβ deposition detectable by PET, together with increasing CSF tau levels and early brain atrophy, typically occurs 15–17 years before clinical manifestation. Cerebral hypometabolism and subtle episodic memory decline often become detectable approximately 10 years before symptom onset, whereas measurable global cognitive impairment typically appears 3–5 years before diagnosis of dementia [21,23,24,25]. This trajectory defines a disease continuum central to early diagnosis.

This sequence reflects the Aβ cascade hypothesis, in which Aβ dysregulation precedes tau hyperphosphorylation, neurodegeneration, and cognitive decline [26]. Under this model, Aβ abnormalities are an early, upstream event, whereas the structural atrophy and metabolic changes most visible on imaging accumulate later as downstream consequences [23]. This staged structure is now formalised in the AT(N) framework, which separates Aβ (A), tau (T) and neurodegeneration (N) markers rather than treating them as interchangeable indicators of a single disease state [27].

Initially, individuals are often classified as cognitively normal (CN) based on standard neuropsychological testing, despite the presence of underlying neuropathology [28,29]. As disease processes progress, some individuals report subjective memory concerns without objective deficits on cognitive testing, a condition referred to as Subjective Memory Complaint (SMC). Although SMC is not considered a formal diagnostic category, longitudinal studies indicate that individuals with SMC have a significantly increased risk of progression to objective cognitive impairment and dementia [30,31,32]. With further neurodegeneration, individuals may belong to the Mild Cognitive Impairment (MCI) stage, an intermediate clinical stage known by measurable cognitive decline that exceeds age- and education-adjusted norms but does not yet significantly impair daily functioning [33,34,35]. Regarding AD, it is accompanied by medial-temporal (hippocampal) atrophy and Aβ/tau positivity, and progresses to dementia at an annual rate of roughly 5–10% [36,37]. Because MCI encompasses a heterogeneous population with distinct clinical trajectories, further subdivision has become common in both clinical and Machine learning (ML) studies [38,39].

MCI is further stratified along two complementary axes. The Alzheimer’s Disease Neuroimaging Institute (ADNI) distinguishes early MCI (EMCI) and late MCI (LMCI) by severity, with LMCI reflecting a more pronounced impairment and is associated with a higher risk of conversion to AD dementia [33,40]. By prognosis, many longitudinal and ML studies further stratify MCI patients into stable MCI (sMCI), whose cognitive impairment remains relatively unchanged during follow-up, and progressive MCI (pMCI), who subsequently convert to AD dementia within the study follow-up period. These clinical categories are practical constructs based on thresholds applied to continuous neuropsychological measures rather than distinct biological events [33,40]. As a result, the boundaries that models are asked to learn, whether between EMCI and LMCI or between sMCI and pMCI, often lie along a biological continuum where differences between groups are relatively subtle. This ambiguity is further compounded by the heterogeneity of the MCI population, as a substantial proportion of individuals diagnosed with MCI do not exhibit underlying AD pathology [38,39,41,42]. This distinction inside the MCI group represents one of the most clinically relevant and challenging prognostic tasks in AD research, as it aims to identify individuals at increased risk of future disease progression [41,42]. Ultimately, disease progression leads to AD, marked by severe cognitive and functional decline, loss of autonomy, and increased mortality [1].

Understanding this clinical continuum is essential for effective diagnosis and management. Early identification of individuals at risk allows for timely intervention, improved care planning and better quality of life for patients and caregivers [43]. Importantly, emerging disease-modifying therapies have demonstrated greater efficacy when administered during early stages of AD, before extensive neuronal loss has occurred [44,45]. Consequently, the development of sensitive, reliable, and cost-effective diagnostic tools capable of detecting AD in its earliest phases has become a major priority in Alzheimer’s research [19].

In recent years, artificial intelligence (AI) has emerged as a promising approach to address the challenges of early AD detection [46]. Traditional ML and deep learning (DL) techniques can identify complex and subtle patterns in high-dimensional biomedical data that may not be apparent through conventional analytical methods [46,47]. Early studies primarily focused on single data modalities. For example, pioneering work demonstrated that computational analysis of MRI features could distinguish AD from CN individuals [48]. However, AD is a multifactorial disease and no single modality can fully capture its biological complexity [49]. Thus, increasing attention has been given to multi-modal AI approaches that integrate different data sources, including neuroimaging, genetic information, fluid biomarkers and cognitive assessments [50,51]. Multi-modal strategies have been shown to improve diagnostic performance, particularly for challenging tasks such as predicting conversion from MCI to AD [41,43]. However, the rapid growth of AI-based methodologies led to increasing datasets heterogeneity, feature engineering strategies, validation procedures and model interpretation approaches, making it difficult to draw robust conclusions about the advantages and clinical applicability of different methods [16,52].

Although numerous reviews have investigated ML, neuroimaging biomarkers or specific AI applications in AD, a comprehensive assessment integrating datasets, data modalities, feature engineering strategies, modelling approaches, validation practices, explainability trends and challenges related to generalizability and clinical translation remains limited [53,54]. In this context, the present work provides a systematic review of AI-based models for the detection and diagnosis of AD. By synthesising evidence from studies published between 2016 and 2026, this review aims to compare single-modal and multi-modal methods, evaluate the impact of different data modalities and algorithms on diagnostic performance and identify methodological limitations that hinder clinical translation. Specifically, this review addresses the following research questions:

- Q1: What are the main methodological trends, data sources and performance characteristics of current AI-based approaches for AD diagnosis and prognosis?
- Q2: Which types of algorithms, including traditional ML and DL, demonstrate superior performance in AD identification?
- Q3: What are the most widely used databases for AD research and how do dataset choices affect model evaluation and generalizability?
- Q4: Which data modalities and modality combinations provide the greatest diagnostic value across different stages of the disease?
- Q5: What feature extraction techniques are most commonly employed and how do they influence model performance?
- Q6: To what extent do current validation strategies and external testing procedures support the generalizability and clinical translation of AI-based AD models?
- Q7: What do these findings tell us about AD itself?

By addressing these questions, this review examines single- and multi-modal AI in AD, identifies key gaps and outlines priorities for improving the robustness and clinical applicability of AI-based diagnostic systems.

## 2. Materials and Methods

### 2.1. Search Strategy

A comprehensive literature search was conducted in early 2026 following PRISMA guidelines. The final search was performed in April 2026 and all retrieved records were assessed for eligibility. Multiple scholarly databases and publisher platforms were used to ensure broad coverage across both biomedical and technical domains. Specifically, we searched PubMed, Google Scholar, Scopus and Web of Science for articles in major publication outlets (including but not limited to Elsevier, Frontiers Media, Oxford University Press, Wiley, ASM, PLOS, Copernicus, Springer Nature, MDPI, American Chemical Society and SAGE). The search queries were constructed using relevant keywords and their combinations with appropriate Boolean operators (e.g., “Alzheimer’s disease classification” AND “machine learning” OR “deep learning” OR “multi-modal” OR “brain imaging” OR “cognitive decline”; additional terms such as “beta-amyloid”, “p-tau”, “cognitive assessments” and “mild cognitive impairment” were also used). The initial search covered publications from 1995 to early 2026 to trace the evolution of AI-based approaches to AD diagnosis.

### 2.2. Eligibility Criteria

We included peer-reviewed research articles that presented an ML or DL model for AD diagnosis or prognosis (e.g., classifying AD vs. cognitively normal or predicting progression from MCI to AD) and reported quantitative evaluation metrics such as classification accuracy, area under the receiver operating characteristic curve (AUC) or related performance measures. We excluded studies that did not involve a predictive modelling component (e.g., papers focused solely on epidemiology, genetic associations or clinical trial outcomes without an AI model) or that lacked quantitative performance results. Conference abstracts, editorials, commentaries, preprints, letters and studies without sufficient methodological detail or quantitative performance reporting were also excluded. Furthermore, although the database search returned records from 1995 onwards, only studies published between 2016 and 2026 were considered eligible for inclusion. This restriction was applied to focus the review on recent methods and datasets and to ensure that the included studies were more directly comparable. Studies published before 2016 were used only for background and context and were not included in the quantitative synthesis.

### 2.3. Study Selection

**Figure 1:**
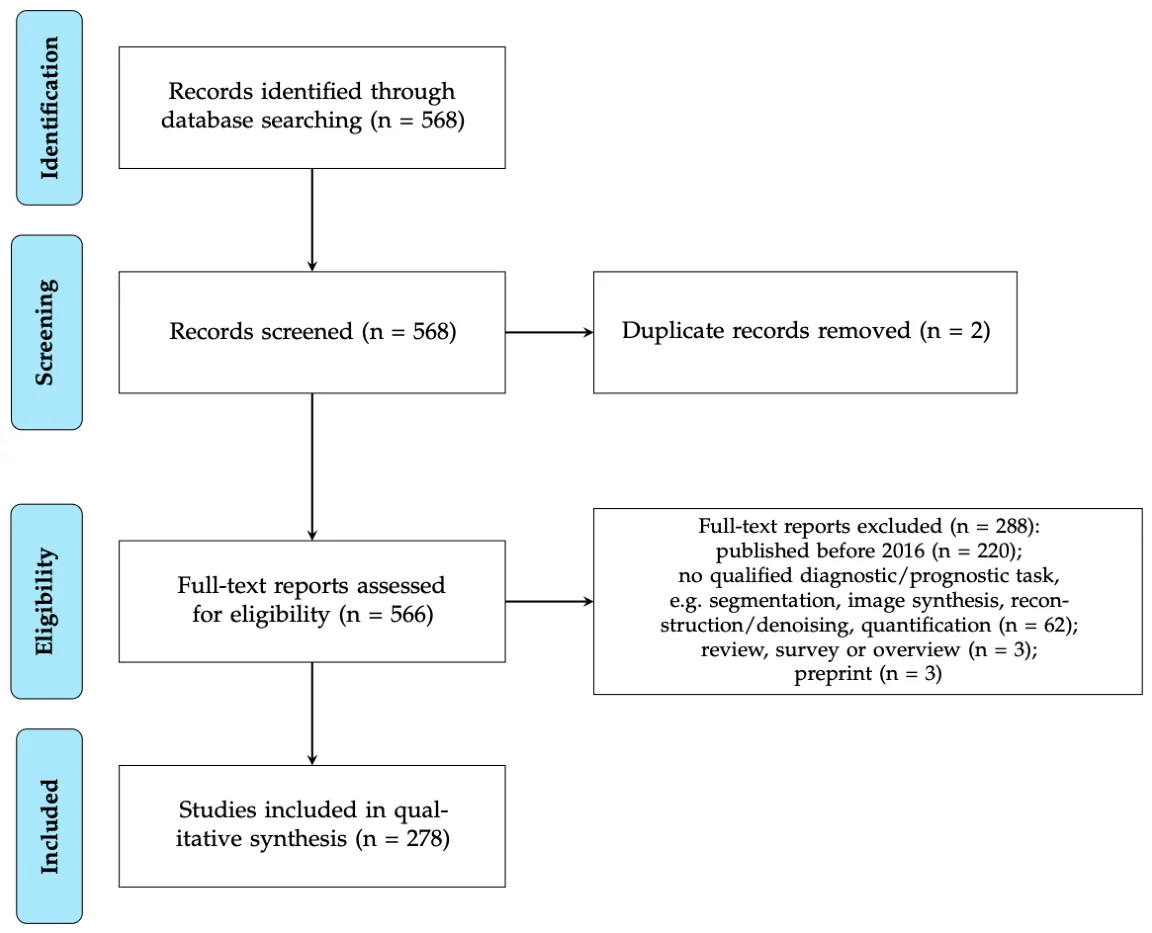
PRISMA flow diagram of the study selection process.

All references identified through the search were imported into a reference management system. Of the 568 records identified, 2 were duplicates, leaving 566 unique records. These records were independently screened by title and abstract, followed by full-text assessment. Each of the 566 unique records was evaluated against the inclusion and exclusion criteria in Section 2.2. Of these, 288 records were excluded: 220 were published before 2016; 62 did not address a qualified diagnostic or prognostic task (e.g., studies whose contribution was image segmentation, cross-modality synthesis, image reconstruction or denoising, quantification or volume-of-interest generation, rather than the classification or prediction of disease status); 3 were reviews, surveys or overviews; and 3 were preprints. Table S1 [48,50,54,55,56,57,58,59,60,61,62,63,64,65,66,67,68,69,70,71,72,73,74,75,76,77,78,79,80,81,82,83,84,85,86,87,88,89,90,91,92,93,94,95,96,97,98,99,100,101,102,103,104,105,106,107,108,109,110,111,112,113,114,115,116,117,118,119,120,121,122,123,124,125,126,127,128,129,130,131,132,133,134,135,136,137,138,139,140,141,142,143,144,145,146,147,148,149,150,151,152,153,154,155,156,157,158,159,160,161,162,163,164,165,166,167,168,169,170,171,172,173,174,175,176,177,178,179,180,181,182,183,184,185,186,187,188,189,190,191,192,193,194,195,196,197,198,199,200,201,202,203,204,205,206,207,208,209,210,211,212,213,214,215,216,217,218,219,220,221,222,223,224,225,226,227,228,229,230,231,232,233,234,235,236,237,238,239,240,241,242,243,244,245,246,247,248,249,250,251,252,253,254,255,256,257,258,259,260,261,262,263,264,265,266,267,268,269,270,271,272,273,274,275,276,277,278,279,280,281,282,283,284,285,286,287,288,289,290,291,292,293,294,295,296,297,298,299,300,301,302,303,304,305,306,307,308,309,310,311,312,313,314,315,316,317,318,319,320,321,322,323,324,325,326,327,328,329,330,331,332,333,334,335,336,337,338,339] lists all the excluded articles from the corpus, together with the reason for their exclusion. After assessing full texts against these criteria, a final set of 278 studies was included in the qualitative synthesis, as shown in Table S2 [5,41,42,51,340,341,342,343,344,345,346,347,348,349,350,351,352,353,354,355,356,357,358,359,360,361,362,363,364,365,366,367,368,369,370,371,372,373,374,375,376,377,378,379,380,381,382,383,384,385,386,387,388,389,390,391,392,393,394,395,396,397,398,399,400,401,402,403,404,405,406,407,408,409,410,411,412,413,414,415,416,417,418,419,420,421,422,423,424,425,426,427,428,429,430,431,432,433,434,435,436,437,438,439,440,441,442,443,444,445,446,447,448,449,450,451,452,453,454,455,456,457,458,459,460,461,462,463,464,465,466,467,468,469,470,471,472,473,474,475,476,477,478,479,480,481,482,483,484,485,486,487,488,489,490,491,492,493,494,495,496,497,498,499,500,501,502,503,504,505,506,507,508,509,510,511,512,513,514,515,516,517,518,519,520,521,522,523,524,525,526,527,528,529,530,531,532,533,534,535,536,537,538,539,540,541,542,543,544,545,546,547,548,549,550,551,552,553,554,555,556,557,558,559,560,561,562,563,564,565,566,567,568,569,570,571,572,573,574,575,576,577,578,579,580,581,582,583,584,585,586,587,588,589,590,591,592,593,594,595,596,597,598,599,600,601,602,603,604,605,606,607,608,609,610,611,612,613]. This systematic review was conducted in accordance with the PRISMA guidelines (PRISMA diagram is presented in Figure 1 and the guidelines check list is at Table S3). The protocol was registered in Propero with the registration number CRD420261484879.

Given the substantial methodological heterogeneity across the included studies, particularly regarding outcome definitions, pre-processing pipelines, feature representations and validation strategies, this review was conducted as a qualitative synthesis rather than a quantitative meta-analysis. Instead of applying a single risk-of-bias instrument, methodological quality and potential sources of bias were assessed narratively throughout the review, with particular attention to dataset selection, validation practices, class imbalance, data leakage and reporting transparency, in line with recent recommendations for AI-based prediction models [18].

### 2.4. Data Extraction and Synthesis

From each of the 278 included studies, we extracted key information including the data modality or modalities used (e.g., MRI, PET, EEG, MEG, CSF biomarkers, cognitive tests), the ML or DL algorithms employed, the data source (e.g., ADNI), the diagnostic or prognostic task, the validation strategy used, the use of explainability methods when applicable, and the reported performance metrics. Each article was reviewed in full and the mentioned information was systematically extracted and recorded in Table S2. If anything was missing or unclear, it was recorded as “not specified” or reported using the wording provided in the article rather than being inferred. Then, data was systematically compared and synthesised in Section 3 which is organised by key themes, including data modality, model type, validation strategy and clinical stage. This approach allowed to highlight current trends, strengths and limitations of AI approaches for AD diagnosis. Moreover, a study was classified as multi-modal when it combined two or more distinct types of data (i.e., PET + CSF) as separate inputs to the model, even if they originated from the same imaging platform. For example, T1-weighted MRI combined with diffusion MRI (DTI) or T2-weighted MRI was considered multi-modal because these sequences capture complementary aspects of brain anatomy and tissue characteristics [614,615,616].

## 3. Results

The next sections synthesise the 278 studies that met the inclusion criteria. To orient the reader, Table 1 summarises how often each analysed aspect appears across the corpus.

### 3.1. Data Sources and Dataset Usage

**Figure 2:**
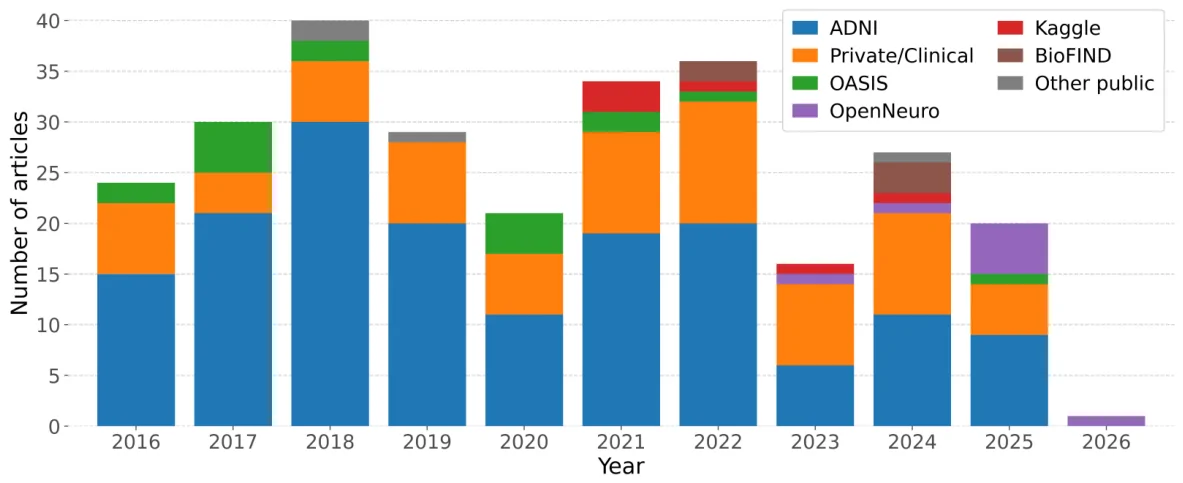
Distribution of dataset type usage among the included studies.

Across the reviewed studies, a small number of datasets accounted for the majority of the published work. As shown in Figure 2, the ADNI was the predominant data source, being used in 160 of the 278 included studies (approximately 58%). Beyond ADNI, institution-specific clinical cohorts represented the second most common data source. These datasets were particularly prevalent in EEG and MEG studies, which are frequently based on local hospital or memory-clinic populations. The Open Access Series of Imaging Studies (OASIS) was the next most used public repository, appearing in 20 studies, while Kaggle-hosted MRI datasets and OpenNeuro-hosted EEG collections were used in 13 and 8 studies, respectively. Several additional datasets, including NACC, AIBL, MIRIAD, BioFIND, BioFINDER, CADDementia and DementiaBank’s Pitt Corpus, appeared only sporadically, each being used in fewer than five studies.

A relatively small proportion of studies employed multiple datasets. In total, only 20 of the 278 reviewed studies (≈8%) relied on more than one data source. In most cases, one cohort was used for model development and another for external validation. For example, Pan et al. [522] trained a model using a local Chinese clinical cohort and validated it on ADNI to assess cross-population generalizability. Similarly, Palmqvist et al. [519] developed a classifier using the BioFINDER cohort and tested it on ADNI as an external validation dataset. Other studies combined multiple datasets to improve cohort diversity and disease-stage representation. Muksimova et al. [509] merged ADNI and OASIS data, while Liu et al. [490] evaluated a model trained on ADNI across independent cohorts including MIRIAD and AIBL. Although these approaches represent important steps toward robust and generalizable AI systems, they remain uncommon within the current literature.

Clear differences were observed between multi- and single-modal studies with respect to dataset selection. Of the 278 studies, 105 used multi-modal approaches integrating more than one data modality. Approximately 69% of these studies relied on ADNI, reflecting the scarcity of alternative repositories that provide multiple well-aligned data types for the same individuals. The main exceptions were some MEG-based studies that combined electrophysiological and neuroimaging data from clinical cohorts or the BioFIND dataset. In contrast, the remaining 173 single-modal studies exhibited substantially greater diversity in dataset usage. Although ADNI remained the most frequently used source (102 studies), many investigations relied on private clinical cohorts, OASIS, Kaggle-hosted MRI datasets, OpenNeuro EEG collections and other specialised repositories. Notably, several datasets, including OpenNeuro, CADDementia, NACC, Kaggle-derived collections and DementiaBank’s Pitt Corpus, appeared exclusively in single-modality studies.

### 3.2. Feature Representation and Extraction

The features used to train classifiers in the reviewed studies ranged from simple hand-crafted descriptors to high-dimensional representations combining multiple data sources. Earlier studies typically relied on carefully engineered features extracted from specific biomarkers or imaging characteristics, whereas more recent approaches increasingly employ large-scale feature sets and automatically learned representations derived from DL models. Table 2 summarises the principal feature extraction strategies reported in the literature, organised according to data modality and their underlying biological rationale. Representative references are included for each feature family, although the cited studies are intended as illustrative examples rather than an exhaustive inventory of the literature.

### 3.3. Classification Methods

The reviewed studies span two broad methodological categories for classification: traditional ML with hand-crafted features and DL approaches with automated feature learning. A smaller subset of studies employed hybrid pipelines, where deep neural networks generate feature representations that are then fed into a conventional classifier (e.g., an support vector machine (SVM) or logistic regression). Traditional ML models generally rely on a smaller number of trainable parameters, making them suitable for modest-sized datasets and often easier to interpret [618], whereas DL models typically require larger training samples and learn more complex representations, usually at the expense of interpretability.

**Figure 3:**
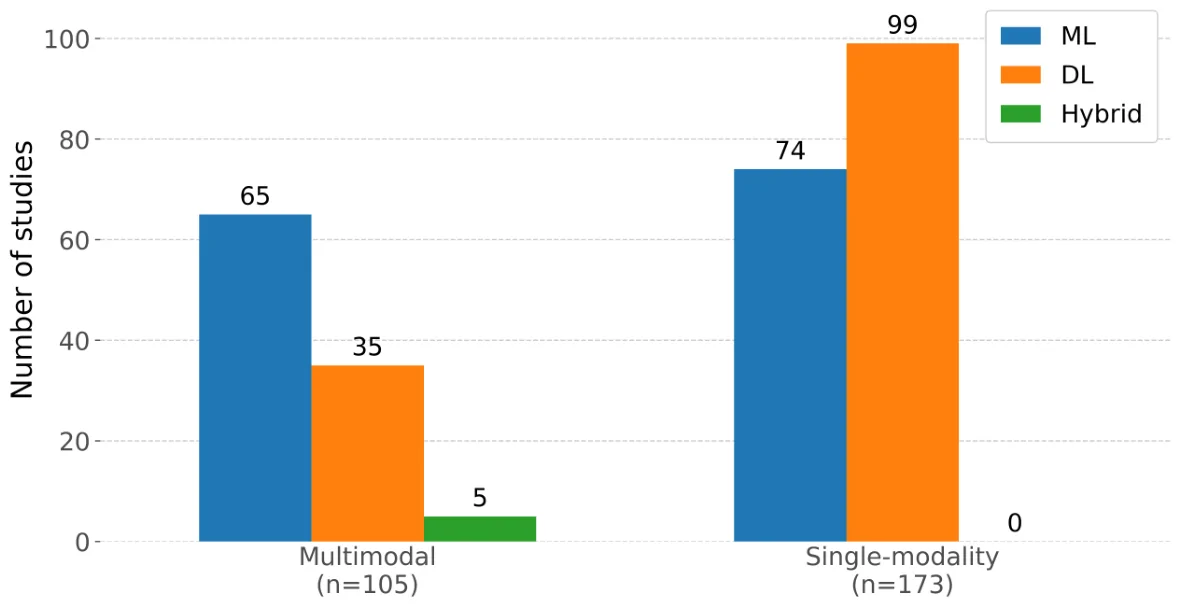
Distribution of classification approaches across the reviewed studies.

As shown in Figure 3, the overall distribution of modelling approaches was relatively balanced, with 139 studies (50.0%) employing classical ML methods and 134 (48.2%) using DL approaches. Nevertheless, distinct patterns emerged when studies were stratified according to the number of modalities considered. Among the 105 multi-modal studies, classical ML remained the main approach, adopted by 65/105, with pure DL in 35/105 and hybrid strategies in the remaining 5/105. The picture inverts for the 173 single-modality studies, with DL taking the lead at 99/173 compared to 74/173 for ML.

Among ML-based studies, the SVM and its variants were by far the most popular classifiers. SVMs (including multi-kernel implementations) were used in 21.9% of all reviewed studies, representing roughly 44% of the ML-only models. Other ML classifiers appeared much less frequently: ensemble methods like random forests and boosting (e.g., AdaBoost, XGBoost) were each used in only 1–4% of total studies, and simpler methods such as logistic regression, linear discriminant analysis, k-nearest neighbours (kNN) and others accounted for less than 2%. In multi-modal contexts, SVMs were often combined with multiple kernel learning techniques to effectively fuse heterogeneous features at the kernel level, whereas single-modality studies typically employed standard linear or radial basis function SVMs since multi-kernel strategies were unnecessary for a single data type.

Convolutional Neural Networks (CNNs) is the most common DL architecture, used in 18.0% of the studies and representing approximately one-third (37%) of the DL-based subset. Many single-modality imaging studies used 2D CNNs with transfer learning (using models like VGG, ResNet or DenseNet pre-trained on large natural image datasets) to classify MRI slices or projections. In the multi-modal DL studies, researchers often designed specialised 3D CNNs or multi-branch networks to handle different data types concurrently. Beyond CNNs, other DL methods played smaller roles: unsupervised deep feature learners (e.g., stacked autoencoders or deep belief networks) and standard fully connected neural networks (MLP-like architectures, including variants like extreme learning machines) were the next most used, each accounting for only a few percent of the studies. Recurrent architectures (Long Short-Term Memory (LSTM), Gated Recurrent Unit (GRU) and other Recurrent Neural Networks (RNNs)) also appeared in a small number of studies (14 as the best-performing model), often applied to sequential or electrophysiological data. Only a few studies used newer architectures such as attention-based models, graph neural networks (GNNs/GCNs), transformers or generative adversarial networks (GANs) for data augmentation. Although still uncommon, their use increased in recent years.

#### Validation Strategies

Robust validation is essential for reliable classifier performance in medical AI, where datasets are often small and imbalanced. Three main validation strategies were identified in the reviewed literature: (i) cross-validation (CV) methods, in which data are repeatedly partitioned into training and test sets (including *k*-fold CV, leave-one-out cross-validation (LOOCV), nested CV, and stratified variants); (ii) single hold-out evaluations, where the dataset is split once into fixed training, validation and test subsets; and (iii) external or independent validation, in which a model trained on one cohort is evaluated on a completely separate dataset. External validation was used in only a 4% of the reviewed studies, limiting the evidence for the generalizability of these models.

**Figure 4:**
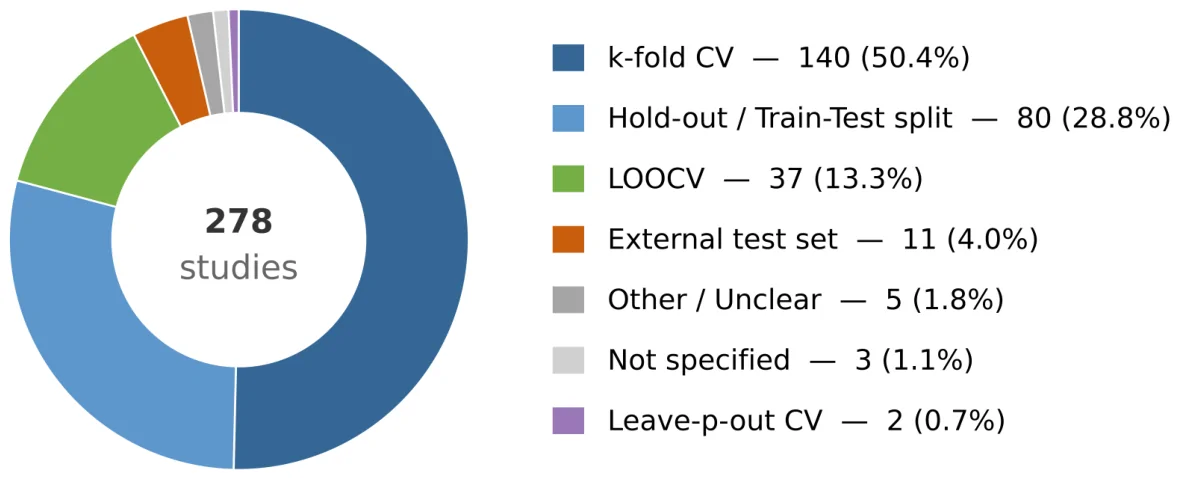
Distribution of internal validation strategies among the reviewed studies.

Overall, *k*-fold CV was the most frequently used evaluation approach, appearing in just over half of the studies (50%). Single hold-out models, including both train–test and train–validation–test splits, represent the second most common strategy, used in 80 studies (approximately 28.8%). Within the CV family, 10-fold CV was the most prevalent configuration (73 studies), followed by 5-fold CV (47 studies) and LOOCV (37 studies). Other values of *k* (such as 3, 4, 6, 9, 15 or 100) were used only sporadically and were typically motivated by specific sample-size constraints. More rigorous validation variants were comparatively rare: nested CV was employed in 11 studies, repeated CV in 36 studies and explicitly stratified folds in 17 studies. These distributions are summarised in Figure 4.

Clear differences emerged between multi-modal and single-modality studies with respect to validation choices. Multi-modal studies relied mainly on *k*-fold CV, whereas single-modality studies more frequently adopted hold-out validation schemes. Validation strategy also varied by data modality. Among MRI-only studies, validation practices were relatively balanced, with 45 studies using *k*-fold CV and 47 employing hold-out splits. A smaller number relied on LOOCV (6 studies) or did not clearly report their validation procedure (2 studies). Studies based on modalities with smaller cohorts or higher feature dimensionality, such as resting-state fMRI or diffusion tensor imaging (DTI), more often used LOOCV to maximise training data in small samples. (e.g., Suk et al. [561], Tang et al. [565] and Chen et al. [382]). A small number of studies using only cognitive, speech or language features tended to apply standard train–validation–test splits, although validation reporting in this subset was occasionally incomplete.

Finally, a clear temporal shift in validation practices was observed from studies published between 2016 and 2017 and in studies from 2024–2025. In early studies, 63.0% used *k*-fold CV and only 18.5% relied on hold-out splits, while LOOCV was still present in 13.3% of studies. By contrast, more recent studies, the use of *k*-fold CV declined to 40.4%, while hold-out strategies increased to 44.7% and LOOCV was relatively uncommon (8.5%).

### 3.4. Overall Model Predictive Scores

**Figure 5:**
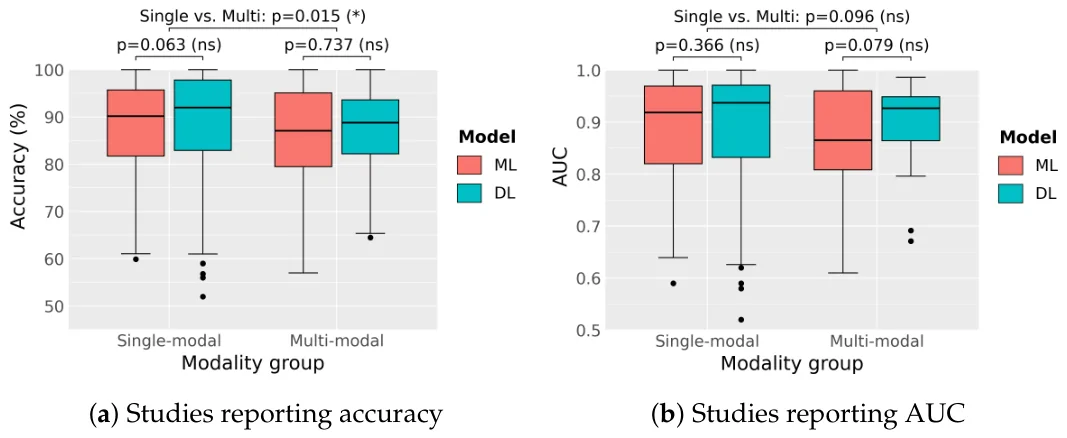
Single- vs. Multi-modal box-plot accuracy and AUC scores. Note: Reported *p*-values were obtained from two-sided Mann–Whitney *U* tests comparing the ML and DL groups. Significance is annotated as: ns, p≥0.05; *, p<0.05.

To provide a quantitative overview of the reported performance across the reviewed literature, a series of box plots were constructed based on Table S2 using the two most frequently reported evaluation metrics: classification accuracy and AUC. Accordingly, Figure 5a,b compare single- vs. multimodal approaches in both ML and DL across the two metrics. It should be noted that not all studies reported both metrics and many of them presented multiple classification tasks (e.g., AD vs. CN and MCI vs. CN). For viewing purposes, each comparison was treated as an independent observation.

Reported classification performance was summarised using the median and interquartile range (IQR) of the best accuracy reported by each study. Results were grouped by task, validation strategy, and database used (Table 3), with task-specific results shown in Table 4. For MCI conversion prediction, the median accuracy was 86.0% for multi-modal studies and 80.9% for single-modal studies. Although not included among the reported results, class balance was considered as an exploratory methodological variable during study assessment to evaluate whether the degree of class imbalance was associated with differences in reported model performance. To classify a dataset as imbalanced, the ratio between the largest and smallest diagnostic groups was calculated using the pooled class counts across all classification tasks reported in each study. Studies with a largest-to-smallest group ratio greater than 2.0 were classified as imbalanced, whereas those with a ratio of 2.0 or less were considered relatively balanced. Under this criterion, 245 of the 278 included studies could be classified according to class balance, of which 156 were considered balanced and 89 imbalanced. Each study was represented by the mean of its reported task-level accuracies and, when available, AUC values. Balanced studies showed a median accuracy of 89.6% (IQR: 83.3–95.3), compared with 88.8% (IQR: 82.4–94.5) for imbalanced studies; the corresponding median AUCs were 0.91 and 0.90, respectively. No statistically significant differences were observed between the two groups [test and *p*-value]. These exploratory findings provide no evidence that the class-balance category, as defined in this analysis, was associated with the performance reported across the reviewed studies.

Beyond the descriptive medians and IQRs, the box plots in Figure 5a,b show statistical significance, helping to interpret the differences between groups. When single- and multi-modal studies were compared as a whole (Figure 5), the difference between the two groups was statistically significant for accuracy (p=0.015), but not for AUC (p=0.090). When ML and DL approaches were compared, no statistically significant differences were found within either the single-modal group (accuracy p=0.063, AUC p=0.366) or the multi-modal group (accuracy p=0.737, AUC p=0.079). These results suggest similar predictive performance across the two algorithm families.

**Figure 6:**
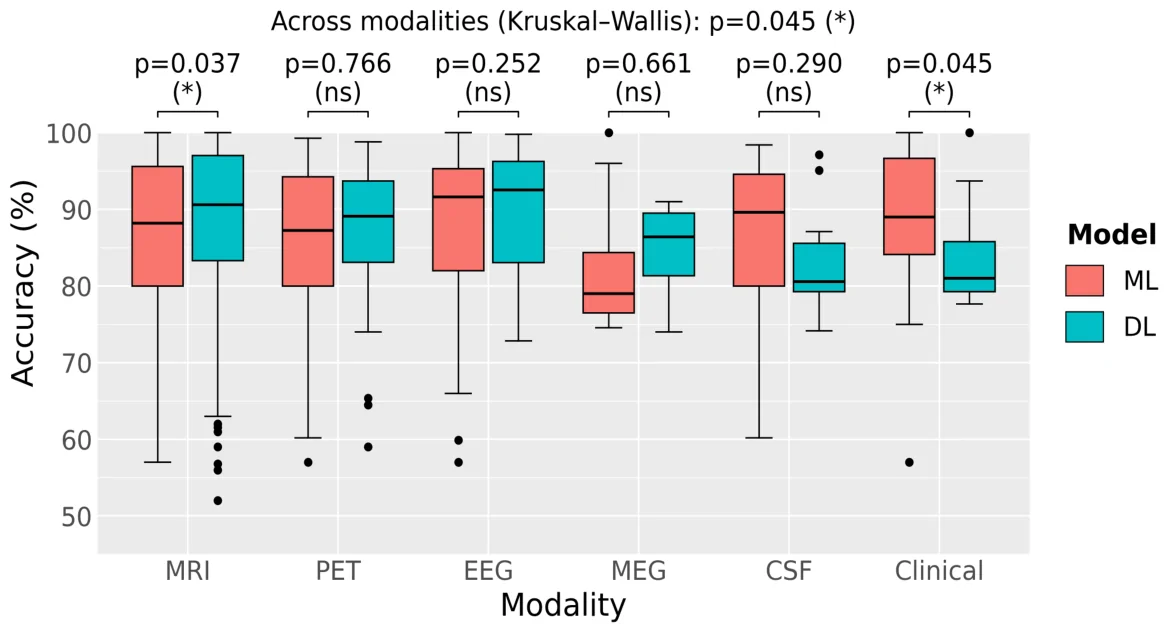
Classification accuracy box plots for ML and DL approaches across PET and MRI studies. Note: *p*-values were obtained using two-sided Mann–Whitney *U* tests for ML vs. DL and Kruskal-Wallis tests across modalities. Significance is shown as: ns, p≥0.05; *, p<0.05.

**Figure 7:**
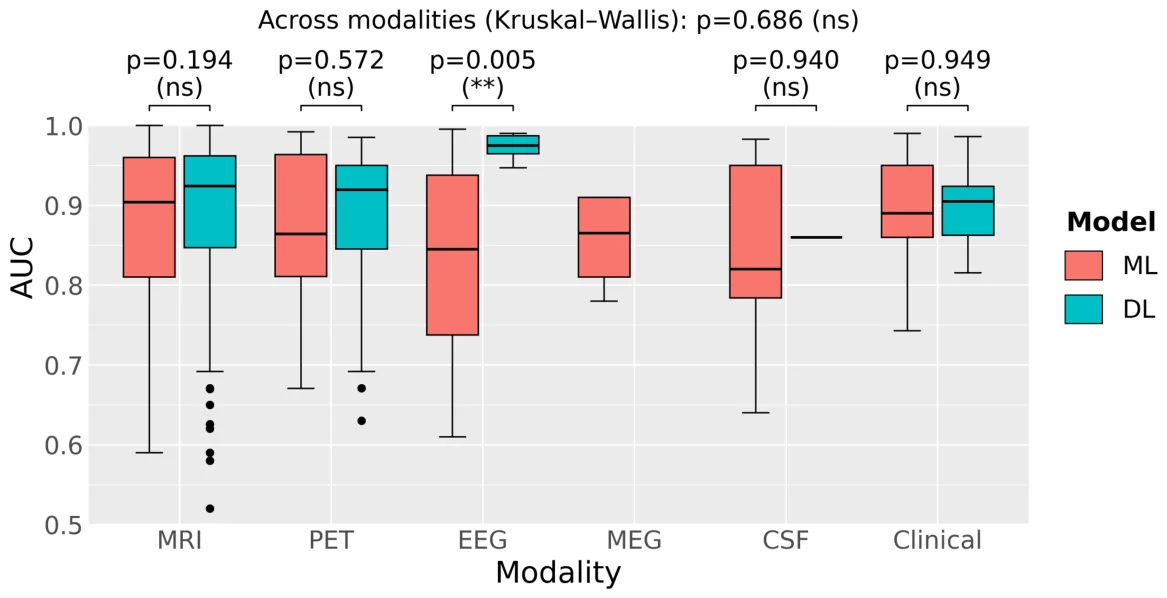
Classification AUC box plots for ML and DL approaches across PET and MRI studies. Note: *p*-values were obtained using two-sided Mann–Whitney *U* tests for ML vs. DL and Kruskal-Wallis tests across modalities. Significance is shown as: ns, p≥0.05; **, p<0.01.

Figure 6 and Figure 7 compare ML and DL performance across data modalities. Studies were grouped by the modalities used, whether alone or in multi-modal settings. Thus, studies combining MRI and PET were included in both analyses. For simplicity, MRI variants (e.g., fMRI and DTI) were grouped as MRI, while all PET modalities were grouped as PET.

For the two dominant imaging modalities, MRI and PET, DL models generally achieved slightly higher median performance than ML approaches. In MRI studies, median accuracy increased from 88.2% (ML) to 90.6% (DL), with median AUC rising from 0.90 to 0.92. A similar pattern was observed for PET, where median accuracy increased from 87.2% to 89.1%, and median AUC from 0.86 to 0.92. EEG studies showed comparable median accuracies for ML and DL methods (91.6% and 92.6%, respectively), although DL models achieved a substantially higher median AUC (0.98 versus 0.85). For CSF data, median accuracy decreased from 89.6% (ML) to 80.6% (DL), whereas median AUC increased slightly from 0.82 to 0.86. Similarly, for clinical and cognitive data, median accuracy declined from 89.0% to 81.0%, while median AUC increased modestly from 0.89 to 0.91. MEG constituted a notable exception, with median accuracy increasing from 79.0% (ML) to 86.4% (DL). Considering all modalities jointly, accuracy differed significantly between ML and DL studies (p=0.045), whereas no significant difference was observed for AUC (p=0.686). At the modality-specific level, significant differences were identified only for MRI accuracy (p=0.037), EEG AUC (p=0.005), and clinical/cognitive data (p=0.045).

## 4. Discussion

Across the studies reviewed, a wide range of methodologies and approaches were used, with differences in their reported performance. Table S2 summarises the main findings of the included studies and provides a comparison of their reported results. The Discussion is organised around the research questions introduced in the Introduction (Q1–Q7), which are discussed throughout the relevant sections.

### 4.1. Overview of the Evidence

Overall, the reviewed studies provide a broad picture of the different ways in which the data can be analysed. Data sources are heavily concentrated: ADNI alone appears in 160 studies (approximately 58%), followed by private clinical cohorts (69 studies) and OASIS (20 studies), while only about 7% of studies (22 of 278) draw on more than one dataset. Imaging remains the dominant modality, but scalp EEG has emerged as the second most common approach, reflecting growing interest in low-cost and portable biomarkers. Other modalities, including PET, fMRI, DRI, fluid biomarkers, cognitive assessments and genetic features, appear quite less. Over a third of studies (105, 38%) were multi-modal however, these typically combined only two data sources, most often sMRI and FDG-PET. Methodologically, classical ML and DL are almost evenly represented (139 studies, 50.0%, vs. 134, 48.2%), with SVMs (21.9% of all studies) and CNNs (18.0%) being the most common algorithms, respectively. The most widely used validation method was k-fold CV (50%), followed by single hold-out splits (about 28.8%), while external validation was relatively rare. The highest reported performance was generally seen for AD vs. CN, with some studies reaching around 99% accuracy. In contrast, more challenging tasks, such as MCI vs. AD or sMCI vs. pMCI, typically presenting accuracies between 70 and 85%. Interestingly, higher reported performance for multi-modal integration does not appear uniformly across classification tasks. The largest differences between multi-modal and single-modal studies were observed in more challenging tasks, such as distinguishing between closely related disease stages (sMCI vs. pMCI). However, because these findings are based on heterogeneous studies rather than direct comparisons under the same conditions, they indicate a trend in the literature rather than a clear advantage of multi-modal fusion itself.

These findings allow to answer *Q1—What are the main methodological trends, data sources, and performance characteristics of current AI-based approaches for AD diagnosis and prognosis?*. The reviewed literature is characterised by a strong reliance on a small number of benchmark datasets, particularly ADNI, and by the continued predominance of neuroimaging-based approaches. At the same time, the growing incorporation of EEG and other complementary biomarkers indicates a gradual broadening of the methodological landscape. From a modelling perspective, both classical ML and DL have become established paradigms, with their relative adoption largely influenced by data availability and modality characteristics. Despite major advances in methodology and very high performance in AD vs. CN classification, important clinical challenges remain, particularly in disease staging and prognosis. Consequently, future progress is likely to depend less on incremental improvements in classification accuracy and more on the development of robust, generalisable and clinically validated AI systems.

### 4.2. Concentrations of Datasets

Table 5 and Table 6 help answer *Q3—What are the most widely used databases for AD research, and how do dataset choices affect model evaluation and generalizability?*. The reviewed literature relies heavily on a small number of cohorts, with ADNI dominating most clinical tasks and modalities. This dependence is particularly evident in multi-modal studies, where sMRI, PET, CSF, cognitive and genetic information are frequently sourced from the same ADNI participants. ADNI’s prominence is largely attributable to its multi-site longitudinal design and the availability of complementary imaging, biomarker and clinical measurements collected from the same individuals. Overall, approximately 69% of multi-modal studies relied on ADNI, whereas single-modality studies were distributed across a wider range of datasets. While this has facilitated methodological comparability, it also raises concerns regarding dataset dependence and the external generalizability of reported results.

This concentration has both advantages and disadvantages. ADNI was designed mainly as a research cohort rather than to represent routine clinical practice. Participants are often selected using strict criteria and standardised protocols, unlike the diverse populations seen in memory clinics. While common datasets allow models to be compared under similar conditions, repeated use of the same or overlapping populations limits their generalisability to new patients.

Thus, although the widespread use of ADNI has facilitated methodological comparison, greater use of independent cohorts and cross-dataset validation is needed to establish the real-world generalisability of AI models for AD.

The lack of independent testing adds to this concern, as only about 8% of studies used more than one dataset, and even fewer performed true external validation on an independent cohort. A few studies did perform cross-cohort validation: Pan et al. [522] trained a model on a Chinese clinical cohort and validated it on ADNI, Palmqvist et al. [519] developed a classifier using BioFINDER and tested it on ADNI, and Liu et al. [490] trained on ADNI and evaluated the model using MIRIAD and AIBL. Until cross-cohort validation becomes more common, the high accuracies reported in the literature should be interpreted with caution, as they may reflect performance under favourable conditions rather than how well models perform on new populations.

### 4.3. From an Imaging-Centric Field to a Broader Modality Landscape

Counting how often each modality appears across the corpus reveals a field in transition rather than a monopoly of a single technique. Although sMRI remains the most widely used modality, EEG emerged as the second most common approach, surpassing PET, CSF biomarkers and genetic information. Its increasing adoption reflects the growing demand for scalable and clinically accessible biomarkers, given its low cost, portability, non-invasive nature, and ability to capture neural dynamics at high temporal resolution, because EEG measures the brain’s electrical activity directly through electrodes on the scalp, revealing a characteristic “slowing” often found in AD patients (a drift of cortical rhythms toward lower frequencies) along with weakened functional coupling between regions [619,620,621,622,623,624,625]. EEG studies were mainly based on single-site hospital cohorts rather than large benchmark datasets, in line with the high use of private datasets for this modality. However, the growing number of publicly available EEG datasets may support more data sharing and reproducible research in the future. MEG occupies an interesting middle ground. Simililarly to this modality, MEG records electrophysiological activity arising from synchronised post-synaptic currents, but instead it senses the magnetic fields these currents generate rather than the voltages they produce at the scalp [626,627,628]. However, MEG was less commonly studied and was based on a small number of datasets and research groups. It was most often combined with sMRI to capture both functional and structural information. PET use remained relatively stable throughout the review period. Most studies used FDG-PET, while tau PET was rarely studied. Other imaging methods were used only occasionally.

Overall, the results show growing use of different biomarkers for AD classification. While sMRI remains the main data source, EEG, MEG, and other biomarkers are receiving more attention as more accessible and scalable alternatives.

### 4.4. Multi-Modal Integration

The reviewed literature is largely motivated by the recognition that AD affects multiple biological systems and therefore cannot be fully characterised using a single biomarker. Nevertheless, the practical adoption of multi-modal approaches remains constrained by data availability and the difficulty of obtaining matched measurements from the same individuals across multiple domains.

Most multi-modal studies combined only two modalities (74 studies), whereas 24 combined three modalities, four studies combined four modalities, and only three integrated five or more data sources. Among two-modality designs, the most common combination was sMRI and FDG-PET (27 studies), reflecting the complementary structural and metabolic information provided by these modalities. Beyond this dominant pairing, other frequently reported combinations included sMRI with fMRI (13 studies) and MEG with sMRI (11 studies). Among three-modality approaches, the most common configuration combined sMRI, FDG-PET and CSF biomarkers. Approaches incorporating cognitive assessments, fluid biomarkers or genetic information were comparatively less common, largely because assembling complete multi-domain datasets remains challenging.

The relative performance of single- and multi-modal approaches is strongly influenced by the clinical task under investigation. Although single-modal studies report slightly higher overall performance when all studies are pooled, this comparison should be interpreted cautiously because the two approaches are often applied to different classification problems. Single-modal models are more frequently evaluated on simpler tasks, particularly AD versus CN classification, where very high performance can already be achieved using a single modality. In contrast, multi-modal approaches are typically applied to more challenging scenarios, such as distinguishing MCI from AD or predicting MCI-to-AD conversion, where complementary information from multiple sources may be required.

A clearer picture emerges when performance is stratified by clinical task (Table 4). Across most tasks, multi-modal approaches performed at least as well as single-modal approaches and often achieved higher median performance. The largest difference was observed for MCI-to-AD conversion, where median accuracy reached 86.0% for multi-modal models compared with 80.9% for single-modal models. These findings suggest that the slightly higher aggregate performance reported for single-modal studies is largely driven by differences in task composition, as single-modal approaches are more frequently evaluated on simpler classification problems. After stratification by task, this apparent advantage largely disappears, illustrating a pattern consistent with Simpson’s paradox, whereby relationships observed in aggregated data may differ from those observed after subgroup analysis [629,630]. Furthermore, relatively few studies directly compare single- and multi-modal approaches under identical experimental conditions. Among those that do, studies such as Vaghari et al. [577] and Jomeiri et al. [613] reported superior performance for multi-modal models, particularly for MCI-versus-CN classification. These improvements are generally attributed to the complementary structural, metabolic, molecular and clinical information provided by different modalities, which may enhance discrimination beyond what can be achieved using any single modality alone.

Several representative studies illustrate the potential of multi-modal integration for clinically challenging tasks. Varatharajah et al. [42] combined six modalities (sMRI, FDG-PET, CSF biomarkers, genetics, cognitive scores and demographics) to predict short-term MCI-to-AD progression with an accuracy of 81% (AUC 0.93). Gupta et al. [425] integrated six imaging-related sources and achieved 98.5% accuracy for AD versus CN and 95.0% for progressive versus stable MCI. Similarly, El-Sappagh et al. [401] combined MRI, FDG-PET, clinical, cognitive and genetic information, obtaining 93.9% accuracy for the three-class CN/MCI/AD problem and 91.9% for pMCI versus sMCI classification.

By contrast, the relatively easier AD-versus-CN task appears close to performance saturation even with limited modality combinations. For example, Fang et al. [405] and Vu et al. [583] reported accuracies of 99.3% and 98.8%, respectively, using only MRI and FDG-PET. The increasing difficulty of distinguishing intermediate disease stages is further illustrated by studies evaluating multiple classification tasks. Zheng et al. [609] achieved 98.70% accuracy for CN versus AD but only 73.82% for MCI versus AD, whereas Khazaee et al. [462] reported 97.46% for AD-versus-rest classification but only 72.03% for MCI-versus-rest classification.

With respect to *Q4—Which data modalities and modality combinations provide the greatest diagnostic value across different stages of the disease?*, the available evidence indicates that no single modality is optimal for all clinical tasks. While sMRI alone frequently achieves very high performance for AD-versus-CN classification, more challenging problems, such as distinguishing closely related disease stages or predicting MCI-to-AD conversion, appear to benefit more consistently from multi-modal integration. The most successful approaches typically combine imaging biomarkers with cognitive, fluid or genetic information, suggesting that their principal advantage lies in capturing complementary aspects of disease biology that become increasingly important as clinical differences become more subtle. However, these findings should be interpreted cautiously, as differences in datasets, cohorts and evaluation protocols may also contribute to the reported performance gains.

### 4.5. Modelling Paradigms and Data Structure

ML and DL are almost evenly represented across the corpus, but this balance reflects a clear inversion driven by the number of modalities a study uses, since among the 105 multi-modal studies, ML remained the most common option (61.7%), with DL representing only 33.6% and hybrid pipelines 4.7%. Conversely, among single-modality studies, DL was the most common approach, accounting for 50.4% of the studies. This divergence might be due to that single-modality studies typically draw on large, homogeneous public repositories, such as ADNI or Kaggle, which supply the volume of data that deep networks require. Multi-modal studies often rely on smaller cohorts and require the integration of heterogeneous features, making them well suited to traditional ML methods, which can offer greater data efficiency, interpretability and flexibility in fusing different types of information. Regarding the choice of algorithms, SVMs, often with multiple-kernel learning, accounted for roughly 44% of ML-based models, while CNNs, frequently with transfer learning, made up about 37% of the DL subset.

Considering the question *Q2—Which types of algorithms, including traditional ML and DL, demonstrate superior performance in AD identification?*, the evidence does not support the existence of a universally superior algorithmic family for AD classification. Instead, performance appears strongly dependent on the interaction between model architecture, data characteristics, sample size and task complexity. DL holds a consistent but modest advantage in imaging-based categories, where it is also the most frequently adopted approach. Median accuracy increases from 88.2% to 90.6% for MRI and from 87.2% to 89.1% for PET, accompanied by corresponding gains in median AUC, as seen in Figure 6. This pattern is reversed for several non-imaging categories, where traditional ML remains highly competitive and, in some cases, achieves higher median performance values than DL. Importantly, these differences do not translate directly into the single- vs. multi-modal comparison, as both families perform similarly within multimodal frameworks. Consequently, reported superiority of one modelling approach over another should be interpreted cautiously, particularly when comparisons are drawn across different datasets, modalities and diagnostic tasks.

### 4.6. Feature Representation and Extraction Strategies

The recent EEG literature provides a notable exception to the broader trend towards DL. Among the 61 EEG studies identified, a majority still relied on classical ML methods, reflecting the long-established use of handcrafted spectral, entropy-based and connectivity-derived features within the electrophysiology community. More broadly, the reviewed literature documents a gradual transition from explicit feature engineering towards automated representation learning. Across the reviewed literature, studies generally followed a common analytical pipeline. However, from approximately 2018 onwards, a gradual transition can be observed from handcrafted feature engineering and conventional machine-learning classifiers towards CNN-based architectures and transfer-learning approaches. Learned representations generated by CNNs and autoencoders are believed to capture latent neurodegenerative patterns that frequently overlap with information traditionally represented through handcrafted features, although in a more complex and non-linear manner. At the same time, newer architectures, including transformers, graph neural networks, attention-based models and generative approaches for data augmentation, are starting to appear in the field, although they are still not widely used. Their appearance coincides with the growing use of explainable AI techniques, including saliency maps and attention visualisation methods. Together, these developments reflect an increasing emphasis on model transparency and interpretability, recognising that clinical adoption will likely depend not only on predictive performance but also on the ability to provide understandable and trustworthy decision support.

Regarding *Q5—What feature extraction techniques are most commonly employed, and how do they influence model performance?*, the reviewed literature suggests that no single feature-extraction strategy consistently outperforms all others across datasets and classification tasks. Structural imaging studies most commonly rely on volumetric, morphometric and ROIs descriptors, whereas electrophysiological studies predominantly employ spectral, entropy-based and connectivity-derived features. Although DL approaches increasingly replace handcrafted features with automatically learned representations, performance appears to depend more on the biological relevance and quality of the extracted information than on the extraction method itself. Consequently, effective control of dimensionality and redundancy remains crutial for developing robust and generalisable AD models.

### 4.7. The Emergence of Explainable AI

A reliable model designed to support dementia diagnosis should not only be accurate but also provide explanations that clinicians can understand and evaluate. Thus, a theme rarely discussed in early literature but has since become an established concern is the use of explainable and interpretable AI (XAI). Accordingly, the studies combine predictive models with methods that explain their decisions, such as SHAP values, attention mechanisms and saliency or activation maps. These methods highlight the regions or modalities influencing predictions, making models easier for clinicians to understand and trust.

This way, 24 studies were found using keywords explicitly related to explainability, tagged with terms such as “XAI”, “explainable AI”, and “interpretable AI”. The temporal distribution of these studies is the most revealing aspect, since XAI is absent from the first five years of the review window (2016 to 2020), whereas XAI begins to appear from 2021 onward, including in studies by El-Sappagh et al. [401], Bloch et al. [377], Lombardi et al. [493], Vlontzou et al. [581] and the remaining ones. In these articles, studies relied almost exclusively on post-hoc methods applied after training, where the SHAP appeared in seven studies and Local Interpretable Model-agnostic Explanations (LIME) in six. Both are model-agnostic methods that estimate the contribution of each input feature to an individual prediction, usually connected with tree-based or kernel classifiers operating on tabular and multi-modal features, where individual biomarkers, cognitive scores and demographic variables can be ranked by importance (e.g., [401]).

For image-based deep models, gradient-based saliency dominated. Gradient-weighted Class Activation Mapping (Grad-CAM) was used in six studies to produce spatial heat maps indicating which regions of an MRI or PET scan most influenced the prediction, while Layer-wise Relevance Propagation (LRP) appeared in four studies. Additional saliency, sensitivity analysis, and occlusion approaches appear in a few other studies. Attention-based maps are also featured in several transformer- and attention-augmented architectures, in which attention weights were displayed as pseudo-saliency maps over the input.

These findings indicate that explainability has evolved from a largely neglected topic before 2020 into an increasingly common component of recent AD-AI studies. Although current approaches remain heterogeneous and lack standardised evaluation frameworks, the growing use of explainability techniques reflects a broader recognition that transparency and interpretability are essential for clinical trust and real-world adoption.

### 4.8. Validation Rigour and Its Evolution

Because AD datasets are often small and have uneven class sizes, the way models are tested can affect how reliable their reported performance is. Across the studies, k-fold CV was the most common method, with 10-fold CV being the most frequently used. Hold-out and LOOCV were also used. More advanced methods were less common: nested CV was used 11 times, repeated CV 36 times, and stratified folds 17 times. Testing models on a separate, independent dataset was the least common approach.

When analysing the considered temporal range, a shifted over time in the validation strategies is noticeable. In studies published during 2016–2017, 63.0% employed k-fold CV and only 18.5% relied on hold-out splits. Between 2024 and 2025, the corresponding proportions were 40.4% and 44.7%, respectively, while LOOCV declined to 8.5%. One possible explanation for this trend is the growing use of DL architectures, for which repeated resampling strategies become computationally demanding. However, the increasing reliance on hold-out evaluation, often performed on relatively small cohorts and rarely complemented by external validation, suggests that advances in model complexity have not been matched by comparable improvements in validation rigour.

Despite the use of external validation in the field, its limited application remains one of the main weaknesses identified in the reviewed studies. Methods such as k-fold CV and hold-out testing mainly measure performance on data from the same dataset. Therefore, they provide limited evidence that a model will work well on data from different scanners, recruitment methods, or demographic groups, which are more similar to real-world use [16,17]. As a result, even studies using external validation often tested their models on datasets with similar data collection methods and patient selection criteria, such as ADNI, AIBL, and MIRIAD. Therefore, there is still limited evidence that many published models can generalise well to new data, and the high accuracies reported should be viewed in the context of the controlled research settings in which they were achieved.

These findings address *Q6—To what extent do current validation strategies and external testing procedures support the generalizability and clinical translation of AI-based AD models?*. The results show that current validation methods are useful for comparing models within research datasets, but they provide limited evidence that these models will work well in real-world settings. The limited use of nested CV and, especially, the lack of external validation reduce confidence in the reported results. More testing on independent cohorts is therefore needed before these AI models can be considered ready for routine clinical use.

### 4.9. Challenges in Interpreting High Reported Performance

Across the reviewed literature, high performance was frequently reported for several AD classification tasks, particularly AD vs. CN classification. Across studies reporting this particular task, the median classification accuracy was approximately 93.5%, with roughly 71% of studies exceeding 90% accuracy and approximately 41% exceeding 95%. Nevertheless, despite these encouraging results, relatively few models have progressed to prospective clinical evaluation or routine clinical application. This observation highlights the challenges involved in interpreting high reported performance within heterogeneous research settings. Reported performance may be influenced by factors such as dataset composition, validation design, cohort characteristics and study-specific methodological choices. Consequently, high reported accuracy should be interpreted together with the underlying study design and validation framework.

#### 4.9.1. Repeated Reliance on a Single Benchmark

As already noted, this field is dominated by a single dataset. ADNI was used in about 58% of studies (160/278) and just over half (roughly 48%) relied on ADNI alone. Also, close to 92% of studies drew on a single database and fewer than 8% combined two or more. Alternative repositories such as OASIS, AIBL, NACC or Kaggle-hosted collections appeared far less frequently. Because ADNI is a research dataset with standardised data collection methods and a relatively similar population—typically highly educated, mostly of European ancestry, and with many patients showing memory-related symptoms—models are often trained and tested on “clean” data with less variation. This does not fully represent the more diverse patients seen in memory clinics or primary care. When researchers repeatedly develop and adjust models using the same dataset, the improvements may partly reflect better performance on that specific benchmark rather than better performance across different populations and data collection settings.

#### 4.9.2. Small Samples and Unstable Performance Estimates

Despite the size of ADNI and the use of different datasets, the effective sample sizes remain modest: approximately 40% of studies have fewer than 200 participants and nearly 25% have fewer than 100. At these sample sizes, CV accuracy carries large uncertainty, with error bars on the order of ±10% expected for around 100 samples, and the standard error across folds systematically underestimates this variability [631]. Consequently, many of the accuracy differences reported between competing architectures fall within the noise floor, and the common practice of foregrounding the single best-performing configuration introduces an optimistic bias that inflates apparent state-of-the-art performance.

#### 4.9.3. Internal Validation over External

Validation practices further inflate reported performance. About 95% of studies relied exclusively on internal validation, either resorting to *k*-fold CV or a single hold-out split drawn from the same dataset, whereas only around 4% evaluated their model on a genuinely independent cohort and roughly 1% did not report their validation scheme clearly enough to be classified. However, internal resampling estimates optimism rather than generalisation, leading to models that appear excellent on one dataset that routinely degrade when applied to cohorts with different scanners, inclusion criteria or demographics [16]. The near-absence of external and, especially, prospective validation means that the generalisation ability of most published models remains fundamentally unknown.

#### 4.9.4. Data Leakage at the Subject Level

A more subtle source of optimistic performance estimation is data leakage between training and test partitions. This issue has been identified as a recurrent methodological concern in previous assessments of AD AI literature. Around 31% of the reviewed studies represented imaging data as slices or patches, an approach that is particularly vulnerable when subject-level separation is not explicitly enforced. Among studies using slice- or patch-based CNN pipelines, reporting of subject-level partitioning was uncommon, making it difficult to assess the extent to which information from the same participant may have been present in both training and testing partitions. This concern is especially relevant for DL models, where large numbers of images are often generated from relatively small cohorts. When partitioning is performed at the image level rather than at the participant level, reported performance may become artificially inflated despite apparently rigorous validation procedures. Similar risks may arise when data augmentation is applied before partitioning or when publicly available image collections do not provide sufficient subject-level information. Importantly, reporting of partitioning strategies remains inconsistent across the literature, which introduces uncertainty when interpreting exceptionally high performance figures. Previous reproducibility analyses have suggested that such methodological issues may partially explain some of the highest reported accuracies and may reduce the apparent advantage of complex DL models when evaluation protocols are applied more rigorously [16,632]. A related concern is the potential circularity between inputs and diagnostic labels, whereby biomarkers used as model inputs may also contribute to the diagnostic definitions used as prediction targets.

#### 4.9.5. Excessive Tuning and Optimistic Reporting

The combination of extensive hyperparameter search, repeated CV, aggressive data augmentation and selective reporting of the best classifier or configuration may further contribute to the mentioned issues. Without a locked, untouched test set, tuning decisions leak test information into model selection, and metrics are reported at the peak of a search rather than as an unbiased expectation. Such practices produce results that are difficult to reproduce and that tend to regress once independent groups reimplement them [631].

#### 4.9.6. Narrow Tasks, Missing Metrics and the Translation Gap

Finally, the tasks and metrics that dominate the literature are not necessarily those that matter most clinically. Balanced AD vs. CN discrimination, where this is most evident, is comparatively easy, whereas clinically important tasks, such as predicting conversion from MCI to AD (pMCI vs. sMCI, eMCI vs. LMCI) or forecasting future decline, are both more difficult and much less common. Reported performance rarely includes calibration, decision-curve or clinical-utility analysis, or evaluation of net benefit at a clinically meaningful operating point. Besides that, majority of studies adhere to established reporting and risk-of-bias standards for AI-based prediction models [18]. These optimistic single-cohort metrics, unaddressed bias, and the lack of evidence required by regulators and clinicians help explain why models reporting ≥95% accuracy in publications have often failed to reach clinical practice or the market. These observations point to a clear methodological priority for future work. Beyond the accuracy and AUC that currently dominate the literature, discrimination metrics should be complemented with calibration assessments, sensitivity and specificity, positive and negative predictive values, and clinically selected decision thresholds evaluated through decision-curve analysis. Direct comparisons against clinicians and established diagnostic workflows would further provide a more robust assessment of real-world clinical benefit and facilitate translation into practice, particularly in settings characterised by class imbalance.

#### 4.9.7. Implications

The performance ceiling should therefore not be seen as evidence that AD diagnosis is solved, but rather as a sign of benchmark saturation. Moving beyond this will require larger, multi-site and demographically diverse cohorts, strict subject-level partitioning, external and ideally, prospective validation and evaluation using clinically meaningful outcomes. Greater attention should also be given to practical aspects such as XAI application, rather than focusing mainly on small improvements in balanced accuracy on a single benchmark.

### 4.10. Interpretation of Observed Literature Patterns

To address the final research question, Q7—What do these findings tell us about AD itself?, four hypotheses may help to contextualise the patterns observed in the reviewed studies. These interpretations should be considered exploratory because the studies were primarily designed to evaluate diagnostic performance rather than disease mechanisms. Consequently, the observations below should not be seen as direct findings of the review.

The first interpretation concerns the persistent 70–85% performance ceiling observed in MCI vs. AD and sMCI vs. pMCI classification tasks. Given its recurrence across diverse architectures, datasets, modalities and feature representations, this observation cannot be fully explained by differences in modelling approaches alone. One possible explanation is that clinical diagnostic labels are imposed on a continuous pathological process. This is reinforced by the heterogeneous nature of the MCI category itself, as a substantial proportion of individuals classified as MCI may not have underlying AD pathology. Under this view, the challenge is not simply that models fail to separate two well-defined disease stages, but that they attempt to identify a sharp boundary within a biological continuum.

A second possible interpretation is that different modalities may provide complementary information across different stages of the disease process. However, the reviewed studies do not directly evaluate disease mechanisms or the temporal sequence of pathological events and, therefore, such interpretation should be viewed cautiously. Whereas MRI-based approaches achieve near-saturated performance in the AD vs. CN classification task, biomarkers such as CSF, plasma and blood markers, together with EEG-derived measures, appear particularly valuable at earlier stages of the disease process. The observed use of EEG in these settings is consistent with previous reports highlighting its potential relevance in early-stage assessment, as spectral slowing and reduced functional connectivity are thought to reflect synaptic dysfunction and large-scale network disruption, processes that are believed to precede measurable neuronal loss. The dominant EEG feature families reinforce this interpretation, since spectral band-power measures and entropy- or complexity-based descriptors represent operationalisations of two well-established observations regarding AD pathophysiology: a shift towards slower cortical rhythms and a reduction in the complexity of neural activity. The limited representation of emerging biomarkers further supports this interpretation. For example, Tau PET appears in only a single study within the reviewed studies, reflecting its relatively recent introduction rather than a lack of biological relevance.

The third interpretation focuses on how reliance on a small number of benchmark cohorts shapes not only model generalizability but also the very phenotype of AD represented in the literature. Longitudinal research cohorts such as ADNI recruit predominantly through memory clinics, enrich for Aβ positivity and commonly exclude individuals with substantial cerebrovascular disease or other neurological comorbidities. In addition, these cohorts are often not fully representative of the broader population. Dementia in unselected older adults, by contrast, is frequently characterised by mixed pathology, with vascular, Lewy body and TDP-43 changes co-occurring alongside AD [3]. Consequently, the models reviewed are generally trained and evaluated on a comparatively pure expression of AD rather than on the more heterogeneous presentations encountered in clinical practice. This discrepancy may contribute to explain the gap between benchmark performance and real-world clinical utility that does not depend exclusively on methodological limitations.

The fourth interpretation relates these findings to the Aβ cascade hypothesis [23,26]. Across the reviewed literature, the features most commonly driving model performance, including structural atrophy from sMRI, hypometabolism from FDG-PET, and spectral slowing from EEG, predominantly reflect downstream [N] markers of neurodegeneration rather than the initiating Aβ pathology. This may help explain why single-modal models often approach performance saturation in AD versus CN classification, where neurodegenerative changes are already extensive, yet show lower performance in sMCI versus pMCI prediction, where discriminative information is expected to emerge earlier in the pathological cascade. Consistent with this interpretation, multi-modal approaches frequently show greater benefit when integrating modalities that capture earlier pathological processes, including amyloid and tau PET, CSF biomarkers, or plasma measures of Aβ and p-tau (Table 4). Several studies explicitly formulate their prediction tasks around this biological framework rather than treating diagnostic labels as biology-agnostic categories. Mathotaarachchi et al. [5] used amyloid PET SUVR to predict incipient dementia, while Shojaie et al. [554] combined amyloid PET, tau PET, and sMRI to approximate the complete AT(N) profile. Others anchored prediction to Aβ status through less invasive proxies. Pan et al. [522] and Palmqvist et al. [519] used plasma biomarkers to predict Aβ pathology and future progression, whereas Menezes et al. [633] stratified participants according to Aβ status to define a six-stage classification framework based solely on sMRI features. These studies illustrate how the Aβ cascade hypothesis can influence task formulation and label definition, rather than serving solely as a post-hoc interpretation of model outputs.

However, this last interpretation framing should be treated with caution, since this review was not properly designed to test hypothesis such as this and that the hypothesis is itself debated: the imperfect correlation between Aβ burden and cognition, and the contribution of tau, neuroinflammation and mixed pathologies, have prompted refinements to the strictly linear view [3], while anti-Aβ therapies that slow but do not halt decline [44,45] suggest Aβ removal alone is not sufficient. The practical implication for AI research is to recognise that a model’s discriminative signal occupies a specific position along a multifactorial cascade, and that its stage-dependent behaviour is more interpretable when that position is made explicit.

Overall, these findings suggest that the challenges reported across many studies may result from a combination of biological differences, dataset characteristics and methodological limitations identified in the reviewed literature. Since the included studies were not designed to directly investigate disease mechanisms, these points should be seen as possible explanations for the patterns observed in the literature rather than as definitive conclusions about AD itself.

### 4.11. Limitations of This Review

Several limitations should be considered when interpreting the findings of this review. Performance values presented in Table S2 are not directly comparable across studies due to substantial cohorts heterogeneity, sample sizes, pre-processing pipelines, validation strategies, outcome definitions and, critically, the definitions of key labels such as the conversion window used to distinguish pMCI from sMCI. Hyperparameter optimization procedures were also not analysed systematically, as their reporting was often incomplete and highly heterogeneous across studies. Since hyperparameter choices can substantially influence model performance, part of the observed variability may reflect differences in model tuning rather than intrinsic methodological superiority. This heterogeneity prevented the conduct of a formal meta-analysis with pooled effect sizes and quantitative estimates of between-study variability. Consequently, the present work should be interpreted as a qualitative synthesis of the available evidence rather than a quantitative meta-analysis. Cross-study comparisons should therefore be viewed as indicative rather than direct comparisons.

The corpus may also favor positive and high-performing results due to publication bias. Studies reporting exceptionally high results are more likely to be published, cited and reused as methodological benchmarks, potentially creating an overly optimistic impression of current AI capabilities. Moreover, studies typically reported only their best-performing configuration so, the reported metrics may reflect optimal tuning rather than expected real-world performance. Additional factors known to influence apparent performance, including subject- vs. image-level evaluation, were too inconsistently reported to permit meaningful stratification across studies. This review also did not apply a formal, structured risk-of-bias instrument to each included study. Instead, the principal methodological concerns, including dataset concentration, limited external validation, potential subject-level data leakage and optimistic reporting, were examined narratively.

The reviewed literature is strongly imaging-centred, with MRI accounting for the majority of studies, with speech and language-based approaches represent only 2% of the studies. Although this distribution reflects broader research trends rather than any selection bias, it inevitably constrains the generalizability of modality-specific conclusions. Consequently, comparative interpretations involving under-represented modalities should be approached with caution. These findings highlight the need for greater methodological attention to non-imaging data sources, whose broader adoption and rigorous evaluation may provide complementary perspectives for AD detection and diagnosis.

Finally, the strong use of ADNI and a small number of other benchmark datasets means that many studies rely on highly overlapping patient populations and similar experimental settings. As a result, the consistency of the reported results may give a stronger impression of reproducibility and generalisability than is actually supported by the evidence. Combined with the publication bias toward positive findings, this may contribute to an overly optimistic view of model performance in the literature. These limitations do not change the main conclusions of this review, but they suggest that absolute performance results should be interpreted with caution.

## 5. Conclusions

This systematic review synthesised 278 studies published between 2016 and 2026 and addressed key questions regarding datasets, modalities, feature engineering strategies, modelling approaches, validation practices and the clinical translation of AI-based models for AD. Four structural findings organise the corpus. First, research remains mainly concentrated on a small number of benchmarks, which has accelerated methodological progress at the cost of external validity, since models are repeatedly trained and evaluated on overlapping populations and only about 8% of studies use more than one dataset. Second, the field is progressively expanding beyond its traditional imaging-centred focus. In particular, electrophysiological approaches, especially scalp EEG, have emerged as one of the fastest-growing areas of research, reflecting increasing interest in scalable, low-cost and clinically accessible biomarkers. Third, methodological choices remain strongly influenced by data characteristics. DL approaches are most commonly associated with large-scale imaging datasets, whereas traditional ML methods continue to perform competitively in smaller and more heterogeneous multi-modal cohorts. Fourth, multi-modal studies most frequently reported performance improvements on the most clinically demanding tasks, distinguishing closely related stages such as MCI and AD and predicting conversion, whereas the easier contrasts are already near ceiling for single-modal models. Importantly, these findings indicate that the principal limitation of the current literature is no longer the ability to achieve high performance, but rather the difficulty of demonstrating robustness and generalizability across independent cohorts and real-world clinical environments.

This way, the reviewed evidences demonstrate that AI has become an indispensable component of contemporary AD research, with the ability to extract clinically relevant information from diverse and complementary data sources. Nevertheless, the field remains constrained by heavy reliance on a limited number of benchmark datasets, insufficient external validation and substantial methodological heterogeneity.

Future progress will depend less on developing increasingly complex algorithms and more on improving dataset diversity, validation rigour, model interpretability and biological grounding. Particular attention should be directed toward clinically meaningful tasks, including early-stage risk prediction, disease-progression forecasting and identification of individuals most likely to benefit from emerging disease-modifying therapies. Achieving these goals will require large, diverse and externally validated multi-modal cohorts capable of supporting robust evaluation across different populations and healthcare settings.

Ultimately, the challenge facing the field is no longer demonstrating that AI can detect AD, but establishing that these systems can do so reliably, transparently and consistently across diverse populations and clinical settings. Future progress will depend less on the development of increasingly complex algorithms and more on improvements in dataset diversity, validation rigour, model interpretability and clinical evaluation. Only through these advances can the considerable diagnostic potential identified throughout the reviewed literature be translated into robust, generalizable and clinically deployable tools capable of supporting the early detection and management of AD.

## Figures and Tables

**Table 1:** Reviewed corpus (n=278) overview, showing the frequency of each aspect. Percentages are based on the total number of included studies. For categories marked “*”, counts are not mutually exclusive, as studies may contribute to multiple rows. Thus, percentages may not sum to 100%.

Aspect	Frequency, *n* (%)
Modelling approach	
Single-modal	173 (62%)
Multi-modal	105 (38%)
Data modality *	
sMRI	199 (71%)
EEG	61 (22%)
PET	48 (17.1%)
Cognitive/clinical/demographic	27 (10%)
Genetic/omics/plasma	32 (11.5%)
Functional MRI (fMRI)	20 (7%)
CSF biomarkers	14 (5%)
Diffusion MRI (DTI/DWI)	13 (5%)
MEG	13 (5%)
Speech/language	5 (2%)
Model family *	
ML	139 (50%)
DL	134 (48%)
Hybrid	5 (2%)
Explainable AI (XAI)	24 (9%)
Longitudinal design	12 (4%)
Data source *	
ADNI	160 (58%)
Private/clinical cohort	82 (29%)
OASIS	20 (7%)
Kaggle-hosted	13 (5%)
OpenNeuro	8 (3%)
AIBL/NACC/MIRIAD (each)	≤5
Multi-dataset (≥2 databases)	22 (8%)
Validation strategy *	
K-fold cross-validation	140 (50%)
Single hold-out/split	80 (29%)
External validation	11 (4%)
Nested cross-validation	11 (4%)

Note: Percentages show how often each modality appears in the reviewed studies. They reflect research activity in the field and do not represent proportions of a curated or balanced dataset.

**Table 2:** Summary of the principal feature representations identified across the reviewed studies.

Modality	Feature Family	Representation and Biological Rationale	Representative Studies
sMRI	Atlas-based volumetric features	Regional grey-matter volumes, mean intensities or related morphometric measures are extracted from predefined anatomical ROIs, commonly using AAL, HAMMER/JHU or FreeSurfer parcellations. These features provide compact and interpretable representations of AD-related regional atrophy.	[496,524,561,599]
sMRI	Targeted ROI and voxel-level features	Targeted approaches restrict analysis to regions strongly implicated in AD, including the hippocampus, entorhinal cortex, precuneus, and temporal areas. Voxel-level representations retain finer spatial information but require feature selection or dimensionality reduction because of their high dimensionality.	[5,482,602,613]
sMRI	Cortical and surface-based features	Cortical thickness, surface area, cortical volume, curvature, gyrification, and sulcal depth characterise morphological changes associated with cortical neurodegeneration, as the loss of neurons, synapses and dendritic arborization in the cortical ribbon manifests directly as a thinning of the grey matter. Targeted measures frequently include hippocampal volume and entorhinal cortical thickness.	[473,475,484,609]
sMRI	Intensity, texture, and frequency-domain features	First-order intensity statistics capture global tissue-composition changes as grey matter is progressively replaced by cerebrospinal fluid, while GLCM and related texture features quantify spatial heterogeneity reflecting microstructural changes such as gliosis that may precede macroscopic atrophy. Wavelet-based descriptors represent abnormalities at multiple spatial scales and may be combined with texture measures.	[354,472,515,542,555,584]
sMRI	DL representations	CNNs, autoencoders, vision transformers, and related architectures learn image representations directly from slices, patches or volumes. Learned embeddings may encode regional atrophy, cortical thinning, ventricular enlargement and other spatial patterns without predefined feature engineering.	[511,613]
PET	FDG-PET metabolic features	Regional uptake ratios and mean tracer intensities reflect cerebral glucose metabolism as an indirect measure of synaptic activity. Characteristic temporoparietal hypometabolism often precedes detectable structural atrophy, making these features informative at prodromal and MCI stages, often aligned with MRI parcellations for multi-modal integration.	[496,524,561,599,612]
PET	Aβ and tau PET features	Amyloid PET measures regional Aβ deposition, while tau PET measures neurofibrillary tau pathology. Regional SUVR values support molecular characterisation and implementation of Aβ, tau and neurodegeneration frameworks.	[5,554]
DRI	Scalar diffusion and tractography features	Fractional anisotropy, mean diffusivity, axial and radial diffusivity, kurtosis measures, fibre counts, and tract-density descriptors characterise white-matter microstructural integrity. Anisotropy typically decreases and diffusivity increases as axonal and myelin barriers break down, providing sensitive markers of white-matter degeneration.	[382,544,565]
DRI	Structural connectivity representations	Diffusion-derived fibre pathways are represented as connectivity matrices or networks, capturing disruption of the structural connections between anatomically defined regions. These may be integrated with functional-connectivity matrices.	[478,503]
rs-fMRI	Functional connectivity and BOLD-derived features	Pairwise correlations between regional BOLD time series generate functional-connectivity matrices that are sensitive to disruption of large-scale networks, notably the default mode network, whose posterior cingulate and medial-temporal synchrony declines early in the disease. ALFF and ReHo quantify regional spontaneous activity and local synchrony, respectively, providing complementary indicators of functional disruption.	[425,450,590]
rs-fMRI	Graph-based representations	Graph-theoretic measures, including degree, clustering, centrality and small-worldness, summarise network organisation. Operate directly on nodes and edges to learn connectivity patterns without reducing the graph to predefined measures.	[357,433,453,462,464]
EEG/MEG	Spectral and band-power features	Absolute or relative power in the delta, theta, alpha, beta, and gamma bands captures the characteristic slowing of neural oscillations associated with AD. Equivalent representations are obtained from MEG power spectral density.	[347,504,546,568,617]
EEG/MEG	Complexity and non-linear features	Entropy, fractal-dimension, chaos-based, and Hjorth descriptors measure signal complexity and irregularity. They reflect the reduced dynamical complexity and increased predictability of neural activity in neurodegeneration.	[359,361,362,404,500,537]
EEG/MEG	Time-frequency features	Wavelet transforms, empirical mode decomposition, variational mode decomposition, and related approaches capture transient and non-stationary oscillatory patterns that may be obscured by global spectral summaries.	[355,422,426,442,508,597]
EEG/MEG	Connectivity and graph features	Coherence, phase-locking value, phase lag indices, amplitude-envelope correlation, and mutual-information measures characterise disrupted functional coupling. Connectivity matrices may also be summarised using graph metrics or processed directly using graph neural networks.	[393,397,516,529,543,548,595,601]
EEG/MEG	Source-level and learned representations	Source reconstruction maps electrophysiological signals onto anatomically defined cortical regions, improving anatomical interpretation. Recent studies also use raw or minimally processed signals as inputs to deep networks that learn representations directly from temporal or spectral data.	[365,379,443,466,492,514]
Fluid biomarkers	CSF biomarkers	CSF representations are dominated by Aβ42, total tau and phosphorylated tau. Aβ42 paradoxically decreases as it is sequestered into cerebral plaques, whereas tau and p-tau rise as they leak from degenerating neurons, with p-tau being the most AD-specific; together these reflect amyloid deposition, neurodegeneration, and tau pathology. These compact vectors are commonly combined with imaging, cognitive, demographic, or genetic information.	[42,356,401,475,561,569,599]
Fluid biomarkers	Plasma biomarkers	Plasma Aβ42, Aβ40, p-tau181, p-tau217, NfL, GFAP and proteomic panels provide less invasive molecular measures and can be joined with cognitive, imaging, and genetic predictors.	[470,519,522]
Cognitive and clinical	Global scores, domains, and subscales	MMSE, CDR, ADAS-Cog, MoCA, RAVLT, FAQ and ECog assess cognition, severity, memory and functional ability. Studies may use total/sub scores, composite measures or individual items; orientation to time and place often declines early due to hippocampal and medial-temporal involvement.	[352,460,475,493,530]
Demographic and genetic	Demographic and APOE features	Age, education, and sex or gender are included as predictors or potential confounders. APOE ϵ4 is usually represented by genotype or allele count and is frequently combined with imaging, cognitive, and molecular features.	[5,41,377,424,457,511]
Genetic	Genome-wide SNP features	Genome-wide or biologically targeted SNP sets represent polygenic AD susceptibility. Their high dimensionality generally requires quality control, filtering, and feature selection before integration with imaging or clinical information.	[340,421,581]
Multi-modal	Concatenated and jointly learned representations	Combination of structural, metabolic, molecular, cognitive, genetic, demographic and electrophysiological information. Fusion ranges from combining compact feature vectors to learning high-dimensional joint representations.	[51,401,421,424,425]
Longitudinal	Rates of change and sequential representations	Repeated observations are represented as annualised changes, visit-level feature sequences or variable-length trajectories. Recurrent architectures, including GRUs and model temporal progression are particularly relevant to MCI-to-AD conversion.	[389,457,476]

Note: Abbreviations: AAL—Automated Anatomical Labelling; AD—Alzheimer’s disease; ALFF—Amplitude of Low Frequency Fluctuations; APOE—Apolipoprotein E; AV-45—florbetapir; Aβ—amyloid-beta; Aβ40—amyloid-beta 40; Aβ42—amyloid-beta 42; BOLD—Blood-Oxygen-Level-Dependent; CDR—Clinical Dementia Rating; CNN—Convolutional Neural Network; CSF—cerebrospinal fluid; DTI—Diffusion Tensor Imaging; DWI—Diffusion-Weighted Imaging; ECog—Everyday Cognition scale; EEG—Electroencephalography; FAQ—Functional Activities Questionnaire; FDG-PET—Fluorodeoxyglucose Positron Emission Tomography; fMRI—functional Magnetic Resonance Imaging; GFAP—Glial Fibrillary Acidic Protein; GLCM—Gray-Level Co-occurrence Matrix; GNN—Graph Neural Network; GRU—Gated Recurrent Unit; HAMMER—Hierarchical Attribute Matching Mechanism for Elastic Registration; JHU—Johns Hopkins University; MEG—Magnetoencephalography; MCI—Mild Cognitive Impairment; MMSE—Mini-Mental State Examination; MoCA—Montreal Cognitive Assessment; MRI—Magnetic Resonance Imaging; NfL—Neurofilament Light chain; PET—Positron Emission Tomography; RAVLT—Rey Auditory Verbal Learning Test; ReHo—Regional Homogeneity; ROI—Region of Interest; rs-fMRI—resting-state functional Magnetic Resonance Imaging; sMRI—structural Magnetic Resonance Imaging; SNP—Single-Nucleotide Polymorphism; SUVR—Standardised Uptake Value Ratio; p-tau—phosphorylated tau; p-tau181—phosphorylated tau 181; p-tau217—phosphorylated tau 217.

**Table 3:** Tiered summary of reported classification performance, expressed as median and IQR, over the 231 studies reporting an explicitly labelled accuracy.

Stratum	Category	Median and IQR Accuracy (%) (*n*)	Median and IQR AUC (*n*)
Validation type	*k*-fold CV	94.0 [89.6–97.2] (159)	0.95 [0.90–0.98] (94)
	Hold-out/split	94.0 [90.3–98.7] (80)	0.95 [0.83–0.99] (25)
	LOOCV	89.0 [83.7–94.2] (37)	0.95 [0.88–0.96] (5)
	Nested CV	84.0 [80.3–93.2] (11)	0.91 [0.91–0.95] (5)
	External validation	92.3 [88.0–98.0] (11)	0.95 [0.91–0.96] (13)
Databases	ADNI	93.3 [88.2–97.2] (155)	0.96 [0.92–0.98] (88)
	Private/clinical	91.7 [84.0–94.8] (58)	0.94 [0.85–0.98] (26)
	OASIS	96.4 [93.2–98.5] (20)	0.95 [0.95–1.00] (5)
	Kaggle	97.6 [90.4–99.8] (13)	0.97 [0.94–0.99] (7)
	OpenNeuro	95.0 [81.7–96.4] (8)	1.00 [1.00–1.00] (2)
	BioFIND	77.0 [74.6–82.7] (5)	0.95 [0.93–0.96] (3)
	NACC	97.4 [94.1–98.2] (4)	0.89 [0.86–0.93] (3)
	Other/unclear	92.0 [82.0–97.5] (30)	0.97 [0.91–0.98] (9)
Class imbalance	Balanced	89.6 [83.3–95.3] (156)	0.91 [0.86–0.97] (66)
	Imbalanced	88.8 [82.4–94.5] (89)	0.90 [0.84–0.95] (44)

**Table 4:** Median and IQR of reported classification accuracy for single- and multi-modal studies, stratified by clinical task. Values represent the task-specific accuracy reported by each study.

Clinical Task	Single-Modal Median and IQR (*n*)	Multi-Modal Median and IQR (*n*)	Δ (pts)
AD vs. CN	91.2 [85.6–96.3] (74)	94.8 [91.8–97.1] (41)	+3.6
MCI vs. CN	86.7 [73.6–92.5] (24)	86.5 [80.1–94.0] (24)	−0.2
MCI-conversion (sMCI/pMCI)	80.9 [74.6–84.1] (15)	86.0 [79.7–93.8] (19)	+5.1
Multi-class (≥3 groups)	91.6 [86.4–98.0] (25)	94.0 [84.1–97.9] (15)	+2.4

Δ = multi-modal median − single-modal median. Values are best-reported, task-specific accuracies from heterogeneous studies and do not constitute paired comparisons.

**Table 5:** Evidence map of dataset used across the reviewed corpus by clinical task.

Dataset	AD vs. CN	MCI vs. CN	pMCI/sMCI	Multi-Class (≥3)	Other	Total
ADNI	60	28	33	84	14	160
Private/clinical	24	11	1	43	5	82
OASIS	12	0	0	8	0	20
Kaggle	2	0	0	12	0	13
OpenNeuro	2	0	0	6	0	8
BioFIND	0	4	0	1	1	5
NACC	1	1	0	2	1	4
Other/unclear	10	3	3	16	2	30

Note: Studies may contribute to multiple cells; therefore, rows and columns do not sum to the corpus total.

**Table 6:** Evidence map of dataset use across the reviewed corpus by data modality.

Dataset	sMRI	fMRI	DTI	PET	EEG	MEG	CSF	Clin./Cog.	Gen./Omics	Total
ADNI	146	17	8	45	1	0	13	26	23	160
Private/clinical	24	5	5	3	52	8	0	9	3	82
OASIS	20	0	0	1	0	0	0	4	1	20
Kaggle	13	0	0	0	0	0	0	1	1	13
OpenNeuro	0	0	0	0	8	0	0	0	0	8
BioFIND	5	0	0	0	0	5	0	0	0	5
NACC	3	0	0	0	0	0	0	2	0	4
Other/unclear	16	0	0	0	10	0	1	7	6	30

Note: Studies may contribute to multiple cells; therefore, rows and columns do not sum to the corpus total.

## Data Availability

No new data were created or analyzed in this study. Data sharing is not applicable to this article.

## Supplementary Materials

The following supporting information can be downloaded at: https://www.mdpi.com/article/10.3390/s26175489/s1, Table S1: Records excluded during full-text screening, with the reason for exclusion [48,50,54,55,56,57,58,59,60,61,62,63,64,65,66,67,68,69,70,71,72,73,74,75,76,77,78,79,80,81,82,83,84,85,86,87,88,89,90,91,92,93,94,95,96,97,98,99,100,101,102,103,104,105,106,107,108,109,110,111,112,113,114,115,116,117,118,119,120,121,122,123,124,125,126,127,128,129,130,131,132,133,134,135,136,137,138,139,140,141,142,143,144,145,146,147,148,149,150,151,152,153,154,155,156,157,158,159,160,161,162,163,164,165,166,167,168,169,170,171,172,173,174,175,176,177,178,179,180,181,182,183,184,185,186,187,188,189,190,191,192,193,194,195,196,197,198,199,200,201,202,203,204,205,206,207,208,209,210,211,212,213,214,215,216,217,218,219,220,221,222,223,224,225,226,227,228,229,230,231,232,233,234,235,236,237,238,239,240,241,242,243,244,245,246,247,248,249,250,251,252,253,254,255,256,257,258,259,260,261,262,263,264,265,266,267,268,269,270,271,272,273,274,275,276,277,278,279,280,281,282,283,284,285,286,287,288,289,290,291,292,293,294,295,296,297,298,299,300,301,302,303,304,305,306,307,308,309,310,311,312,313,314,315,316,317,318,319,320,321,322,323,324,325,326,327,328,329,330,331,332,333,334,335,336,337,338,339]; Table S2: Summary of the reviewed studies on ML-based classification of AD and related dementias, ordered by data modality (neuroimaging first, followed by EEG/MEG) [5,41,42,51,340,341,342,343,344,345,346,347,348,349,350,351,352,353,354,355,356,357,358,359,360,361,362,363,364,365,366,367,368,369,370,371,372,373,374,375,376,377,378,379,380,381,382,383,384,385,386,387,388,389,390,391,392,393,394,395,396,397,398,399,400,401,402,403,404,405,406,407,408,409,410,411,412,413,414,415,416,417,418,419,420,421,422,423,424,425,426,427,428,429,430,431,432,433,434,435,436,437,438,439,440,441,442,443,444,445,446,447,448,449,450,451,452,453,454,455,456,457,458,459,460,461,462,463,464,465,466,467,468,469,470,471,472,473,474,475,476,477,478,479,480,481,482,483,484,485,486,487,488,489,490,491,492,493,494,495,496,497,498,499,500,501,502,503,504,505,506,507,508,509,510,511,512,513,514,515,516,517,518,519,520,521,522,523,524,525,526,527,528,529,530,531,532,533,534,535,536,537,538,539,540,541,542,543,544,545,546,547,548,549,550,551,552,553,554,555,556,557,558,559,560,561,562,563,564,565,566,567,568,569,570,571,572,573,574,575,576,577,578,579,580,581,582,583,584,585,586,587,588,589,590,591,592,593,594,595,596,597,598,599,600,601,602,603,604,605,606,607,608,609,610,611,612,613]; Table S3: PRISMA 2020 Checklist.
