# Supplementary material for: From Single-Modal to Multi-Modal Artificial Intelligence in Alzheimer’s Disease: A Systematic Review of Databases, Modalities, Diagnostic Performance, and Clinical Translation Challenges
Source: Sensors (Basel). 2026 Aug 29;26(17):5489. doi: 10.3390/s26175489 (PMC13568021; doi:10.3390/s26175489)
Supplement: Supplementary file 1 [file sensors-26-05489-s001.zip › Table S1.pdf]

Table S1: Records excluded during full-text screening, with the reason for exclusion.

Reference	Title	Year	Reason for Exclusion
[107]	Discriminant analysis of MRI measures as a method to determine the presence of dementia of the Alzheimer type	1995	Study before 2016
[169]	Midline cerebral morphometry distinguishes frontotemporal dementia and Alzheimer's disease	1997	Study before 2016
[48]	MR image texture analysis applied to the diagnosis and tracking of Alzheimer's disease	1998	Study before 2016
[307]	Hippocampal volume discriminates between normal cognition; questionable and mild dementia in the elderly	2001	Study before 2016
[71]	Volumetric MRI measurements can differentiate Alzheimer's disease, mild cognitive impairment, and normal aging	2002	Study before 2016
[241]	Hippocampus and entorhinal cortex in mild cognitive impairment and early AD	2004	Study before 2016
[74]	Large deformation diffeomorphic metric mapping of vector fields	2005	Study before 2016
[295]	Altered functional connectivity in early Alzheimer's disease: a resting-state fMRI study	2006	Study before 2016
[147]	Small-world anatomical networks in the human brain revealed by cortical thickness from MRI	2007	Study before 2016
[183]	Hippocampal shape analysis of Alzheimer disease based on ML methods	2007	Study before 2016

Continued on next page

Reference	Title	Year	Reason for Exclusion
[296]	Large deformation diffeomorphism and momentum based hippocampal shape discrimination in dementia of the Alzheimer type	2007	Study before 2016
[124]	Structural and functional biomarkers of prodromal Alzheimer's disease: a high-dimensional pattern classification study	2008	Study before 2016
[207]	Support vector machine-based classification of Alzheimer's disease from whole-brain anatomical MRI	2008	Study before 2016
[113]	MRI-based automated computer classification of probable AD versus normal controls	2008	Study before 2016
[125]	Spatial patterns of brain atrophy in MCI patients, identified via high-dimensional pattern classification, predict subsequent cognitive decline	2008	Study before 2016
[294]	Alzheimer's disease diagnosis in individual subjects using structural MR images: validation studies	2008	Study before 2016
[173]	Automatic classification of MR scans in Alzheimer's disease	2008	Study before 2016
[214]	Collaborative computational anatomy: an MRI morphometry study of the human brain via diffeomorphic metric mapping	2008	Study before 2016

Continued on next page

Reference	Title	Year	Reason for Exclusion
[182]	Automated cortical thickness measurements from MRI can accurately separate Alzheimer's patients from normal elderly controls	2008	Study before 2016
[64]	Automated method for identification of patients with Alzheimer's disease based on three-dimensional MR images	2008	Study before 2016
[320]	Patterns of structural complexity in Alzheimer's disease and frontotemporal dementia	2008	Study before 2016
[248]	Parallel transport in diffeomorphisms distinguishes the time-dependent pattern of hippocampal surface deformation due to healthy aging and the dementia of the Alzheimer's type	2008	Study before 2016
[103]	Detection of prodromal Alzheimer's disease via pattern classification of magnetic resonance imaging	2008	Study before 2016
[104]	Individual patient diagnosis of AD and FTD via high-dimensional pattern classification of MRI	2008	Study before 2016
[153]	Combining voxel-based morphometry with artificial neural network theory in the application research of diagnosing Alzheimer's disease	2008	Study before 2016
[204]	Neurological image classification for the Alzheimer's disease diagnosis using kernel PCA and support vector machines	2009	Study before 2016

Continued on next page

Reference	Title	Year	Reason for Exclusion
[133]	Multidimensional classification of hippocampal shape features discriminates Alzheimer's disease and mild cognitive impairment from normal aging	2009	Study before 2016
[152]	Differential automatic diagnosis between Alzheimer's disease and frontotemporal dementia based on perfusion SPECT images	2009	Study before 2016
[149]	Spatially augmented LPboosting for AD classification with evaluations on the ADNI dataset	2009	Study before 2016
[128]	Morphological hippocampal markers for automated detection of Alzheimer's disease and mild cognitive impairment converters in magnetic resonance images	2009	Study before 2016
[150]	MKL for robust multi-modality AD classification	2009	Study before 2016
[212]	Alzheimer disease: quantitative structural neuroimaging for detection and prediction of clinical and structural changes in mild cognitive impairment	2009	Study before 2016
[249]	Early diagnosis of Alzheimer's disease using cortical thickness: impact of cognitive reserve	2009	Study before 2016
[324]	Regional white matter integrity differentiates between vascular dementia and Alzheimer disease	2009	Study before 2016

Continued on next page

Reference	Title	Year	Reason for Exclusion
[216]	Baseline and longitudinal patterns of brain atrophy in MCI patients, and their use in prediction of short-term conversion to AD: results from ADNI	2009	Study before 2016
[108]	Automated MRI measures identify individuals with mild cognitive impairment and Alzheimer's disease	2009	Study before 2016
[205]	Automatic tool for Alzheimer's disease diagnosis using PCA and Bayesian classification rules	2009	Study before 2016
[267]	Classification results of artificial neural networks for Alzheimer's disease detection	2009	Study before 2016
[242]	Automated detection of brain atrophy patterns based on MRI for the prediction of Alzheimer's disease	2010	Study before 2016
[114]	Amnestic MCI future clinical status prediction using baseline MRI features	2010	Study before 2016
[174]	Boosting power for clinical trials using classifiers based on multiple biomarkers	2010	Study before 2016
[246]	Multimodal EEG, MRI and PET data fusion for Alzheimer's disease diagnosis	2010	Study before 2016
[144]	Individual prediction of cognitive decline in mild cognitive impairment using support vector machine-based analysis of diffusion tensor imaging data	2010	Study before 2016

Continued on next page

Reference	Title	Year	Reason for Exclusion
[336]	Divergent network connectivity changes in behavioural variant frontotemporal dementia and Alzheimer's disease	2010	Study before 2016
[226]	Use of SVM methods with surface-based cortical and volumetric subcortical measurements to detect Alzheimer's disease	2010	Study before 2016
[219]	Hippocampal atrophy patterns in mild cognitive impairment and Alzheimer's disease	2010	Study before 2016
[252]	Computer-aided diagnosis system for the Alzheimer's disease based on partial least squares and random forest SPECT image classification	2010	Study before 2016
[314]	Computer-aided diagnosis of Alzheimer's disease using multiple features with artificial neural network	2010	Study before 2016
[203]	An automatic unsupervised classification of MR images in Alzheimer's disease	2010	Study before 2016
[179]	Identification of a blood-based biomarker panel for classification of Alzheimer's disease	2011	Study before 2016
[59]	An MRI-derived definition of MCI-to-AD conversion for long-term, automatic prognosis of MCI patients	2011	Study before 2016
[91]	Local MRI analysis approach in the diagnosis of early and prodromal Alzheimer's disease	2011	Study before 2016

Continued on next page

Reference	Title	Year	Reason for Exclusion
[95]	Automated hippocampal shape analysis predicts the onset of dementia in mild cognitive impairment	2011	Study before 2016
[98]	Identification of conversion from mild cognitive impairment to Alzheimer's disease using multivariate predictors	2011	Study before 2016
[100]	Automatic classification of patients with Alzheimer's disease from structural MRI: a comparison of ten methods using the ADNI database	2011	Study before 2016
[105]	Prediction of MCI to AD conversion, via MRI, CSF biomarkers, and pattern classification	2011	Study before 2016
[120]	ML classification of MRI features of Alzheimer's disease and mild cognitive impairment subjects to reduce the sample size in clinical trials	2011	Study before 2016
[151]	Predictive markers for AD in a multi-modality framework: an analysis of MCI progression in the ADNI population	2011	Study before 2016
[175]	Multi-template tensor-based morphometry: application to analysis of Alzheimer's disease	2011	Study before 2016
[213]	Mild cognitive impairment: baseline and longitudinal structural MR imaging measures improve predictive prognosis	2011	Study before 2016

Continued on next page

Reference	Title	Year	Reason for Exclusion
[302]	Sensitivity and specificity of medial temporal lobe visual ratings and multivariate regional MRI classification in Alzheimer's disease	2011	Study before 2016
[303]	AddNeuroMed and ADNI: similar patterns of Alzheimer's atrophy and automated MRI classification accuracy in Europe and North America	2011	Study before 2016
[304]	Multivariate analysis of MRI data for Alzheimer's disease, mild cognitive impairment and healthy controls	2011	Study before 2016
[308]	Multi-method analysis of MRI images in early diagnostics of Alzheimer's disease	2011	Study before 2016
[315]	Independent component analysis-based classification of Alzheimer's disease MRI data	2011	Study before 2016
[50]	Multimodal classification of Alzheimer's disease and mild cognitive impairment	2011	Study before 2016
[79]	Efficient mining of association rules for the early diagnosis of Alzheimer's disease	2011	Study before 2016
[138]	Computer-aided diagnosis system for Alzheimer's disease using brain diffusion tensor imaging features selected by Pearson's correlation	2011	Study before 2016
[300]	Enriched white matter connectivity networks for accurate identification of MCI patients	2011	Study before 2016

Continued on next page

Reference	Title	Year	Reason for Exclusion
[325]	ODVBA: optimally-discriminative voxel-based analysis	2011	Study before 2016
[55]	Effects of hardware heterogeneity on the performance of SVM Alzheimer's disease classifier	2011	Study before 2016
[224]	Multi-modal MRI analysis with disease-specific spatial filtering: initial testing to predict mild cognitive impairment patients who convert to Alzheimer's disease	2011	Study before 2016
[254]	Classification of Alzheimer's disease from structural MRI using sparse logistic regression with optional spatial regularisation	2011	Study before 2016
[318]	Semi-supervised pattern classification: application to structural MRI of Alzheimer's disease	2011	Study before 2016
[76]	High dimensional classification of structural MRI Alzheimer's disease data based on large scale regularisation	2011	Study before 2016
[288]	A two-stage sequential ensemble applied to the classification of Alzheimer's disease based on MRI features	2011	Study before 2016

Continued on next page

Reference	Title	Year	Reason for Exclusion
[83]	Classification of Alzheimer's disease, mild cognitive impairment, and normal cognitive status with large-scale network analysis based on resting-state functional MR imaging	2011	Study before 2016
[115]	Combined evaluation of FDG-PET and MRI improves detection and differentiation of dementia	2011	Study before 2016
[234]	NMF-SVM based CAD tool applied to functional brain images for the diagnosis of Alzheimer's disease	2012	Study before 2016
[143]	Multi-scale classification of disease using structural MRI and wavelet transform	2012	Study before 2016
[96]	Scoring by nonlocal image patch estimator for early detection of Alzheimer's disease	2012	Study before 2016
[191]	Ensemble sparse classification of Alzheimer's disease	2012	Study before 2016
[326]	Multi-modal multi-task learning for joint prediction of multiple regression and classification variables in Alzheimer's disease	2012	Study before 2016
[68]	Generative-discriminative basis learning for medical imaging	2012	Study before 2016
[92]	Individual subject classification for Alzheimer's disease based on incremental learning using a spatial frequency representation of cortical thickness data	2012	Study before 2016

Continued on next page

Reference	Title	Year	Reason for Exclusion
[93]	Does feature selection improve classification accuracy? Impact of sample size and feature selection on classification using anatomical magnetic resonance images	2012	Study before 2016
[97]	Simultaneous segmentation and grading of anatomical structures for patient's classification: application to Alzheimer's disease	2012	Study before 2016
[123]	Prediction of conversion from mild cognitive impairment to Alzheimer's disease dementia based upon biomarkers and neuropsychological test performance	2012	Study before 2016
[176]	Improved classification of Alzheimer's disease data via removal of nuisance variability	2012	Study before 2016
[301]	Prediction of Alzheimer's disease and mild cognitive impairment using cortical morphological patterns	2012	Study before 2016
[305]	Regional magnetic resonance imaging measures for multivariate analysis in Alzheimer's disease and mild cognitive impairment	2012	Study before 2016
[306]	Combining MRI and CSF measures for classification of Alzheimer's disease and prediction of mild cognitive impairment conversion	2012	Study before 2016

Continued on next page

Reference	Title	Year	Reason for Exclusion
[309]	non-linear dimensionality reduction combining MR imaging with non-imaging information	2012	Study before 2016
[80]	Functional brain image classification using association rules defined over discriminant regions	2012	Study before 2016
[81]	Association rule-based feature selection method for Alzheimer's disease diagnosis	2012	Study before 2016
[223]	Using support vector machines with multiple indices of diffusion for automated classification of mild cognitive impairment	2012	Study before 2016
[145]	Individual classification of mild cognitive impairment subtypes by support vector machine analysis of white matter DTI	2012	Study before 2016
[61]	Partial least squares for discrimination in fMRI data	2012	Study before 2016
[262]	The relevance voxel machine (RVoxM): a self-tuning Bayesian model for informative image-based prediction	2012	Study before 2016
[245]	Computer-based classification of MR scans in first-time applicant Alzheimer patients	2012	Study before 2016
[208]	Identification of brain regions responsible for Alzheimer's disease using a self-adaptive resource allocation network	2012	Study before 2016

Continued on next page

Reference	Title	Year	Reason for Exclusion
[184]	Discriminant analysis of longitudinal cortical thickness changes in Alzheimer's disease using dynamic and network features	2012	Study before 2016
[77]	Classification of structural MRI images in Alzheimer's disease from the perspective of ill-posed problems	2012	Study before 2016
[99]	Automated detection of amnestic mild cognitive impairment in community-dwelling elderly adults: a combined spatial atrophy and white matter alteration approach	2012	Study before 2016
[327]	Predicting future clinical changes of MCI patients using longitudinal and multimodal biomarkers	2012	Study before 2016
[102]	Discriminative analysis of early Alzheimer's disease using multi-modal imaging and multi-level characterization with multi-classifier (M3)	2012	Study before 2016
[109]	Identification of mild Alzheimer's disease through automated classification of structural MRI features	2012	Study before 2016
[285]	A method for detecting interstructural atrophy correlation in MRI brain images	2012	Study before 2016
[250]	Imputation and classification of missing data using least square support vector machines: a new approach in dementia diagnosis	2012	Study before 2016

Continued on next page

Reference	Title	Year	Reason for Exclusion
[70]	Classification of Alzheimer's disease from 3D structural MRI data	2012	Study before 2016
[237]	Dimensionality reduced cortical features and their use in the classification of Alzheimer's disease and mild cognitive impairment	2012	Study before 2016
[275]	Detecting global and local hippocampal shape changes in Alzheimer's disease using statistical shape models	2012	Study before 2016
[140]	Multi-region analysis of longitudinal FDG-PET for the classification of Alzheimer's disease	2012	Study before 2016
[279]	MicroRNAs circulate around Alzheimer's disease	2013	Study before 2016
[116]	Meta-analysis based SVM classification enables accurate detection of Alzheimer's disease across different clinical centers using FDG-PET and MRI	2013	Study before 2016
[228]	LVQ-SVM based CAD tool applied to structural MRI for the diagnosis of the Alzheimer's disease	2013	Study before 2016
[268]	Deformation based feature selection for computer aided diagnosis of Alzheimer's disease	2013	Study before 2016
[56]	How early can we predict Alzheimer's disease using computational anatomy?	2013	Study before 2016

Continued on next page

Reference	Title	Year	Reason for Exclusion
[57]	Different multivariate techniques for automated classification of MRI data in Alzheimer's disease and mild cognitive impairment	2013	Study before 2016
[122]	Prediction of Alzheimer's disease in subjects with mild cognitive impairment from the ADNI cohort using patterns of cortical thinning	2013	Study before 2016
[192]	Locally linear embedding (LLE) for MRI based Alzheimer's disease classification	2013	Study before 2016
[281]	An MRI-based index to measure the severity of Alzheimer's disease-like structural pattern in subjects with mild cognitive impairment	2013	Study before 2016
[321]	Accurate multimodal probabilistic prediction of conversion to Alzheimer's disease in patients with mild cognitive impairment	2013	Study before 2016
[82]	Integrating discretization and association rule-based classification for Alzheimer's disease diagnosis	2013	Study before 2016
[101]	Spatial and anatomical regularization of SVM: a general framework for neuroimaging data	2013	Study before 2016
[316]	Discrimination between Alzheimer's disease and mild cognitive impairment using SOM and PSO-SVM	2013	Study before 2016

Continued on next page

Reference	Title	Year	Reason for Exclusion
[193]	Hierarchical fusion of features and classifier decisions for Alzheimer's disease diagnosis	2013	Study before 2016
[117]	Robust automated detection of microstructural white matter degeneration in Alzheimer's disease using machine learning classification of multicenter DTI data	2013	Study before 2016
[141]	Random forest-based similarity measures for multi-modal classification of Alzheimer's disease	2013	Study before 2016
[72]	Classification of Alzheimer's disease from FDG-PET images using favourite class ensembles	2013	Study before 2016
[217]	Enhanced compressibility of EEG signal in Alzheimer's disease patients	2013	Study before 2016
[121]	Machine learning-based method for personalized and cost-effective detection of Alzheimer's disease	2013	Study before 2016
[253]	Detection of Alzheimer's disease through automated hippocampal segmentation	2013	Study before 2016
[206]	Selecting and extracting effective features for automated diagnosis of Alzheimer's disease	2013	Study before 2016
[282]	Deep learning-based feature representation for AD/MCI classification	2013	Study before 2016
[283]	Latent feature representation with stacked auto-encoder for AD/MCI diagnosis	2013	Study before 2016

Continued on next page

Reference	Title	Year	Reason for Exclusion
[142]	Delayed ripening and improved fruit processing quality in tomato by RNAi-mediated silencing of three homologs of 1-aminopropane-1-carboxylate synthase gene	2013	Study before 2016; outside the scope of the review
[238]	Dimensionality reduced cortical features and their use in predicting longitudinal changes in Alzheimer's disease	2013	Study before 2016
[292]	Binary classification of 18F-flutemetamol PET using machine learning: comparison with visual reads and structural MRI	2013	Study before 2016
[84]	Development of a brain MRI-based hidden Markov model for dementia recognition	2013	Study before 2016
[180]	Classification of diffusion tensor images for the early detection of Alzheimer's disease	2013	Study before 2016
[161]	Manifold regularized multi-task feature selection for multi-modality classification in Alzheimer's disease	2013	Study before 2016
[209]	Automatic detection and classification of Alzheimer's disease from MRI scans using principal component analysis and artificial neural networks	2013	Study before 2016
[162]	Topological graph kernel on multiple thresholded functional connectivity networks for mild cognitive impairment classification	2013	Study before 2016

Continued on next page

Reference	Title	Year	Reason for Exclusion
[243]	Integrative EEG biomarkers predict progression to Alzheimer's disease at the MCI stage	2013	Study before 2016
[269]	Index of alpha/theta ratio of the electroencephalogram: a new marker for Alzheimer's disease	2013	Study before 2016
[328]	Classification of Alzheimer disease based on structural magnetic resonance imaging by kernel support vector machine decision tree	2014	Study before 2016
[270]	A Bayesian network decision model for supporting the diagnosis of dementia, Alzheimer's disease and mild cognitive impairment	2014	Study before 2016
[289]	Multiple instance learning for classification of dementia in brain MRI	2014	Study before 2016
[293]	A new approach for Alzheimer's disease diagnosis by using association rule over PET images	2014	Study before 2016
[163]	Integration of network topological and connectivity properties for neuroimaging classification	2014	Study before 2016
[148]	Regions of interest computed by SVM wrapped method for Alzheimer's disease examination from segmented MRI	2014	Study before 2016
[181]	Online learning for classification of Alzheimer disease based on cortical thickness and hippocampal shape analysis	2014	Study before 2016

Continued on next page

Reference	Title	Year	Reason for Exclusion
[126]	An ensemble-of-classifiers based approach for early diagnosis of Alzheimer's disease: classification using structural features of brain images	2014	Study before 2016
[85]	Detecting brain structural changes as biomarker from magnetic resonance images using a local feature based SVM approach	2014	Study before 2016
[257]	Predictive models based on support vector machines: whole-brain versus regional analysis of structural MRI in Alzheimer's disease	2014	Study before 2016
[231]	A comparison of three brain atlases for MCI prediction	2014	Study before 2016
[215]	Multi-atlas based representations for Alzheimer's disease diagnosis	2014	Study before 2016
[190]	Brain region's relative proximity as marker for Alzheimer's disease based on structural MRI	2014	Study before 2016
[185]	An efficient approach for differentiating Alzheimer's disease from normal elderly based on multicenter MRI using gray-level invariant features	2014	Study before 2016
[177]	New approach for automatic classification of Alzheimer's disease, mild cognitive impairment and healthy brain magnetic resonance images	2014	Study before 2016

Continued on next page

Reference	Title	Year	Reason for Exclusion
[62]	ApoE4 effects on automated diagnostic classifiers for mild cognitive impairment and Alzheimer's disease	2014	Study before 2016
[251]	Novel ThickNet features for the discrimination of amnestic MCI subtypes	2014	Study before 2016
[186]	Discriminative analysis of multivariate features from structural MRI and diffusion tensor images	2014	Study before 2016
[322]	Multi-task linear programming discriminant analysis for the identification of progressive MCI individuals	2014	Study before 2016
[194]	Inter-modality relationship constrained multi-modality multi-task feature selection for Alzheimer's disease and mild cognitive impairment identification	2014	Study before 2016
[329]	Integrative analysis of multi-dimensional imaging genomics data for Alzheimer's disease prediction	2014	Study before 2016
[63]	Detection of Alzheimer disease in MR images using structure tensor	2014	Study before 2016
[276]	Distance-informed metric learning for Alzheimer's disease staging	2014	Study before 2016
[195]	Multiple kernel learning in the primal for multimodal Alzheimer's disease classification	2014	Study before 2016

Continued on next page

Reference	Title	Year	Reason for Exclusion
[258]	Longitudinal FDG-PET features for the classification of Alzheimer's disease	2014	Study before 2016
[58]	Identifying regions of interest for discriminating Alzheimer's disease from mild cognitive impairment	2014	Study before 2016
[261]	Extracting salient brain patterns for imaging-based classification of neurodegenerative diseases	2014	Study before 2016
[337]	An optimal decisional space for the classification of Alzheimer's disease and mild cognitive impairment	2014	Study before 2016
[284]	Hierarchical feature representation and multimodal fusion with deep learning for AD/MCI diagnosis	2014	Study before 2016
[196]	Early diagnosis of Alzheimer's disease with deep learning	2014	Study before 2016
[187]	Abnormal changes of multidimensional surface features using multivariate pattern classification in amnestic mild cognitive impairment patients	2014	Study before 2016
[338]	A novel matrix-similarity based loss function for joint regression and classification in AD diagnosis	2014	Study before 2016
[278]	Quantifying anatomical shape variations in neurological disorders	2014	Study before 2016

Continued on next page

Reference	Title	Year	Reason for Exclusion
[188]	Hierarchical interactions model for predicting mild cognitive impairment (MCI) to Alzheimer's disease (AD) conversion	2014	Study before 2016
[160]	Sparse kernel entropy component analysis for dimensionality reduction of neuroimaging data	2014	Study before 2016
[94]	Evolutionary ELM wrapper feature selection for Alzheimer's disease CAD on anatomical brain MRI	2014	Study before 2016
[229]	Automatic ROI selection in structural brain MRI using SOM 3D projection	2014	Study before 2016
[287]	Multimodal imaging in Alzheimer's disease: validity and usefulness for early detection	2015	Study before 2016; review, survey or overview
[236]	A lattice computing approach to Alzheimer's disease computer assisted diagnosis based on MRI data	2015	Study before 2016
[220]	Diffusion weighted imaging-based maximum density path analysis and classification of Alzheimer's disease	2015	Study before 2016
[330]	Detection of Alzheimer's disease by displacement field and machine learning	2015	Study before 2016
[331]	Detection of subjects and brain regions related to Alzheimer's disease using 3D MRI scans based on eigenbrain and machine learning	2015	Study before 2016

Continued on next page

Reference	Title	Year	Reason for Exclusion
[218]	Machine learning framework for early MRI-based Alzheimer's conversion prediction in MCI subjects	2015	Study before 2016
[170]	Identifying patients with Alzheimer's disease using resting-state fMRI and graph theory	2015	Study before 2016
[78]	Gaussian process classification of Alzheimer's disease and mild cognitive impairment from resting-state fMRI	2015	Study before 2016
[127]	Boosting diagnosis accuracy of Alzheimer's disease using high dimensional recognition of longitudinal brain atrophy patterns	2015	Study before 2016
[69]	Probability distribution function-based classification of structural MRI for the detection of Alzheimer's disease	2015	Study before 2016
[286]	Baseline shape diffeomorphometry patterns of subcortical and ventricular structures in predicting conversion of mild cognitive impairment to Alzheimer's disease	2015	Study before 2016
[264]	Magnetic resonance imaging biomarkers for the early diagnosis of Alzheimer's disease: a machine learning approach	2015	Study before 2016

Continued on next page

Reference	Title	Year	Reason for Exclusion
[136]	Inclusion of neuropsychological scores in atrophy models improves diagnostic classification of Alzheimer's disease and mild cognitive impairment	2015	Study before 2016
[172]	Applying automated MR-based diagnostic methods to the memory clinic: a prospective study	2015	Study before 2016
[139]	A multi-contrast MRI study of microstructural brain damage in patients with mild cognitive impairment	2015	Study before 2016
[247]	Brain connectivity and novel network measures for Alzheimer's disease classification	2015	Study before 2016
[165]	Automated classification to predict the progression of Alzheimer's disease using whole-brain volumetry and DTI	2015	Study before 2016
[232]	Effects of imaging modalities, brain atlases and feature selection on prediction of Alzheimer's disease	2015	Study before 2016
[89]	Domain transfer learning for MCI conversion prediction	2015	Study before 2016
[230]	Exploratory graphical models of functional and structural connectivity patterns for Alzheimer's disease diagnosis	2015	Study before 2016
[323]	Multimodal discrimination of Alzheimer's disease based on regional cortical atrophy and hypometabolism	2015	Study before 2016

Continued on next page

Reference	Title	Year	Reason for Exclusion
[164]	Manifold regularized multitask feature learning for multimodality disease classification	2014	Study before 2016
[339]	Multi-view Classification for Identification of Alzheimer's Disease	2015	Study before 2016
[312]	Multi-modality sparse representation-based classification for Alzheimer's disease and mild cognitive impairment	2015	Study before 2016
[118]	Multimodal analysis of functional and structural disconnection in Alzheimer's disease using multiple kernel SVM	2015	Study before 2016
[211]	Improved diagnostic multimodal biomarkers for Alzheimer's disease and mild cognitive impairment	2015	Study before 2016
[197]	Multimodal neuroimaging feature learning for multiclass diagnosis of Alzheimer's disease	2015	Study before 2016
[189]	A robust deep model for improved classification of AD/MCI patients	2015	Study before 2016
[198]	View-centralized multi-atlas classification for Alzheimer's disease diagnosis	2015	Study before 2016
[280]	Early detection of Alzheimer's disease using MRI hippocampal texture	2015	Study before 2016

Continued on next page

Reference	Title	Year	Reason for Exclusion
[73]	Predicting conversion from MCI to AD with FDG-PET brain images at different prodromal stages	2015	Study before 2016
[235]	Volume of interest-based [18F]fluorodeoxyglucose PET discriminates MCI converting to Alzheimer's disease from healthy controls: a European Alzheimer's Disease Consortium (EADC) study	2015	Study before 2016
[335]	Novel cortical thickness pattern for accurate detection of Alzheimer's disease	2015	Study before 2016
[90]	Multimodal manifold-regularized transfer learning for MCI conversion prediction	2015	Study before 2016
[297]	Feed-forward neural network optimized by hybridization of PSO and ABC for abnormal brain detection	2015	Study before 2016
[135]	A novel method for early diagnosis of Alzheimer's disease based on pseudo Zernike moment from structural MRI	2015	Study before 2016
[266]	DeepAD: Alzheimer's Disease Classification via Deep Convolutional Neural Networks using MRI and fMRI	2016	preprint
[298]	Detection of Alzheimer's disease by three-dimensional displacement field estimation in structural magnetic resonance imaging	2015	Study before 2016

Continued on next page

Reference	Title	Year	Reason for Exclusion
[260]	HIPS: a new hippocampus subfield segmentation method	2017	Automated hippocampal subfield labelling; output is per-subfield volumes
[156]	An Ensemble of Deep Convolutional Neural Networks for Alzheimer's Disease Detection and Classification	2017	preprint
[199]	Applications of deep learning to MRI images: a survey	2018	Review, survey or overview
[166]	Adaptive template generation for amyloid PET using a deep learning approach	2018	Generates adaptive templates for amyloid PET spatial normalisation
[119]	KIKI-net: cross-domain convolutional neural networks for reconstructing undersampled magnetic resonance images	2018	Reconstructs undersampled MRI via cross-domain CNNs
[54]	Deep learning in Alzheimer's disease: diagnostic classification and prognostic prediction using neuroimaging data	2019	Review, survey or overview
[137]	Hippocampal segmentation for brains with extensive atrophy using three-dimensional convolutional neural networks	2019	Segments the hippocampus in severely atrophied AD brains; output is an anatomical mask, not a diagnosis
[65]	Fast and precise hippocampus segmentation through deep convolutional neural network ensembles and transfer learning	2019	CNN ensemble delineating the hippocampus; contribution is segmentation speed and accuracy
[233]	Ultra-low-dose PET reconstruction using generative adversarial network with feature matching and task-specific perceptual loss	2019	GAN reconstructing amyloid PET from ultra-low-dose data; output is a restored image enabling reduced tracer dose

Continued on next page

Reference	Title	Year	Reason for Exclusion
[265]	Accurate, rapid and reliable, fully automated MRI brainstem segmentation for application in multiple sclerosis and neurodegenerative diseases	2019	Automated brainstem segmentation providing volumetric measurements across neurodegenerative cohorts
[200]	A combined deep-learning and lattice Boltzmann model for segmentation of the hippocampus in MRI	2020	Hybrid CNN plus lattice Boltzmann model for hippocampal delineation
[310]	Segmentation of tau stained Alzheimer's brain tissue using convolutional neural networks	2020	Segments tau-stained tissue in digitised brain sections
[291]	Diffeomorphic registration with intensity transformation and missing data: application to 3D digital pathology of Alzheimer's disease	2020	Diffeomorphic registration aligning 3D digital pathology to MRI, handling missing data
[157]	GAN-based synthetic brain PET image generation	2020	GAN generating synthetic brain PET images for augmentation and simulation
[130]	Deep residual inception encoder-decoder network for medical imaging synthesis	2020	Cross-modality medical image synthesis network
[75]	Hippocampus segmentation on epilepsy and Alzheimer's disease studies with multiple convolutional neural networks	2021	Compares CNNs for hippocampus segmentation across epilepsy and AD cohorts
[222]	Combined atlas and convolutional neural network-based segmentation of the hippocampus from MRI according to the ADNI harmonized protocol	2021	Atlas-plus-CNN hippocampal segmentation following the ADNI Harmonized Protocol

Continued on next page

Reference	Title	Year	Reason for Exclusion
[311]	Learning to synthesise the ageing brain without longitudinal data	2021	Synthesises aged brain MRI without requiring longitudinal training data
[332]	Improving sensitivity of arterial spin labelling perfusion MRI in Alzheimer's disease using transfer learning of deep learning-based ASL denoising	2021	Denoises arterial spin labelling perfusion MRI to improve sensitivity
[159]	Restoration of amyloid PET images obtained with short-time data using a generative adversarial networks framework	2021	GAN restoring amyloid PET images acquired with shortened scan times
[110]	DeepAtrophy: teaching a neural network to detect progressive changes in longitudinal MRI of the hippocampal region in Alzheimer's disease	2021	Self-supervised network measuring progressive hippocampal change between timepoints
[317]	A deep learning framework identifies dimensional representations of Alzheimer's disease from brain structure	2021	Unsupervised representation learning deriving dimensional AD phenotypes from structure
[66]	The impact of transfer learning on 3D deep learning convolutional neural network segmentation of the hippocampus in mild cognitive impairment and Alzheimer disease subjects	2022	Segments the hippocampus; the AI output is an anatomical mask and the study tests transfer learning on segmentation accuracy
[146]	Hippocampus segmentation using U-Net convolutional network from brain magnetic resonance imaging (MRI)	2022	U-Net that delineates the hippocampus on MRI
[210]	A novel deep learning based hippocampus subfield segmentation method	2022	Segments hippocampal subfields, producing volumetric measurements

Continued on next page

Reference	Title	Year	Reason for Exclusion
[259]	A benchmark for hypothalamus segmentation on T1-weighted MR images	2022	Public benchmark for hypothalamus segmentation; evaluates segmentation methods, not diagnosis
[154]	Semisupervised white matter hyperintensities segmentation on MRI	2022	Semi-supervised segmentation of white matter hyperintensity lesions
[178]	BrainSec: automated brain tissue segmentation pipeline for scalable neuropathological analysis	2022	Automated grey/white matter segmentation pipeline for whole-slide neuropathology
[221]	P-ResUnet: segmentation of brain tissue with purified residual U-Net	2022	Brain tissue segmentation network; contribution is the residual architecture
[313]	Intraretinal layer segmentation using cascaded compressed U-Nets	2022	Cascaded compressed U-Nets for intraretinal layer segmentation
[271]	MR template-based individual brain PET volumes-of-interest generation neither using MR nor using spatial normalization	2022	Generates individual PET volumes-of-interest without spatial normalisation
[272]	Unified deep learning-based mouse brain MR segmentation: template-based individual brain positron emission tomography volumes-of-interest generation without spatial normalization in mouse Alzheimer model	2022	Mouse-brain MR segmentation plus inverse spatial normalisation to generate PET VOIs
[129]	Fast three-dimensional image generation for healthy brain aging using diffeomorphic registration	2022	Uses diffeomorphic registration to generate images of healthy brain ageing

Continued on next page

Reference	Title	Year	Reason for Exclusion
[255]	Degenerative adversarial neuroimage nets for brain scan simulations: application in ageing and dementia	2022	Adversarial model simulating brain scans over ageing and dementia progression
[273]	Deep residual inception encoder-decoder network for amyloid PET harmonization	2022	Harmonises amyloid PET across tracers and scanners so measurements are comparable
[106]	Deep learning based low-activity PET reconstruction of [11C]PiB and [18F]FE-PE2I in neurodegenerative disorders	2022	Reconstructs low-activity PET, enabling reduced tracer dose
[319]	Motion correction in MR image for analysis of VSRAD using generative adversarial network	2022	GAN-based motion correction of MR images prior to VSRAD analysis
[167]	Fast and accurate amyloid brain PET quantification without MRI using deep neural networks	2022	Quantifies amyloid PET burden without MRI; output is a continuous measurement
[277]	Generative adversarial network constrained multiple loss autoencoder: a deep learning-based individual atrophy detection for Alzheimer's disease and mild cognitive impairment	2022	GAN-constrained autoencoder producing individual-level atrophy maps
[86]	RBS-Net: hippocampus segmentation using multi-layer feature learning with the region, boundary and structure loss	2023	Hippocampus segmentation using region, boundary and structure losses
[299]	DeepHipp: accurate segmentation of hippocampus using 3D dense-block based on attention mechanism	2023	Hippocampus segmentation network; contribution is the attention-based architecture

Continued on next page

Reference	Title	Year	Reason for Exclusion
[227]	Preanalytic variable effects on segmentation and quantification machine learning algorithms for amyloid-beta analyses on digitized human brain slides	2023	Tests how pre-analytic slide variables affect segmentation and quantification algorithms; the AI is the object under study
[132]	Automatic segmentation of retinal layers in multiple neurodegenerative disorder scenarios	2023	Segments retinal layers on OCT across neurodegenerative conditions
[131]	Brain status transferring generative adversarial network for decoding individualized atrophy in Alzheimer's disease	2023	GAN that generates individualised atrophy maps by transferring brain status
[256]	Accelerated MRI using intelligent protocolling and subject-specific denoising applied to Alzheimer's disease imaging	2023	Accelerates MRI acquisition via intelligent protocolling and subject-specific denoising
[60]	Alzheimer's disease detection using deep learning on neuroimaging: a systematic review	2024	Review, survey or overview
[263]	Fully automated hippocampus segmentation using T2-informed deep convolutional neural networks	2024	Produces hippocampal label maps from T2 MRI; evaluated on overlap with manual tracing
[244]	Attention-gated 3D CapsNet for robust hippocampal segmentation	2024	Hippocampal segmentation architecture; output is a delineated structure, scored by Dice
[112]	Multi-stage semi-supervised learning enhances white matter hyperintensity segmentation	2024	Multi-stage semi-supervised learning to improve WMH lesion delineation

Continued on next page

Reference	Title	Year	Reason for Exclusion
[333]	Anat-SFSeg: anatomically-guided superficial fiber segmentation with point-cloud deep learning	2024	Segments superficial white matter fibre bundles from tractography point clouds
[155]	Automated deep learning segmentation of neuritic plaques and neurofibrillary tangles in Alzheimer disease brain sections using a proprietary software	2024	Segments neuritic plaques and neurofibrillary tangles in histology sections
[134]	Preprint; Learning precise segmentation of neurofibrillary tangles from rapid manual point annotations	2024	Learns tangle segmentation from sparse manual point annotations
[334]	G-protein signaling in Alzheimer's disease: spatial expression validation of semi-supervised deep learning-based computational framework	2024	Semi-supervised framework used to validate spatial gene expression patterns, not to diagnose
[171]	Improving 18F-FDG PET quantification through a spatial normalization method	2024	Deep learning spatial normalisation method to improve FDG PET quantification
[201]	Subject-aware PET denoising with contrastive adversarial domain generalization	2024	Subject-aware PET denoising with adversarial domain generalisation
[202]	Cross noise level PET denoising with continuous adversarial domain generalization	2024	PET denoising generalising across noise levels
[274]	Enhancing amyloid PET quantification: MRI-guided super-resolution using latent diffusion models	2024	MRI-guided super-resolution via latent diffusion to improve amyloid PET quantification

Continued on next page

Reference	Title	Year	Reason for Exclusion
[240]	Super-resolving and denoising 4D flow MRI of neurofluids using physics-guided neural networks	2024	Physics-guided super-resolution and denoising of 4D flow MRI of neurofluids
[168]	Clinical performance evaluation of an artificial intelligence-powered amyloid brain PET quantification method	2024	Clinical performance evaluation of an AI amyloid PET quantification tool
[111]	Regional deep atrophy: using temporal information to automatically identify regions associated with Alzheimer's disease progression from longitudinal MRI	2024	Extends regional deep atrophy using temporal information to map progression regions
[67]	DLMUSE: robust brain segmentation in seconds using deep learning	2025	Whole-brain multi-structure segmentation tool; contribution is speed and robustness of the parcellation
[87]	Quantitative assessment of brain glymphatic imaging features using deep learning-based EPvs. segmentation and DTI-ALPS analysis in Alzheimer's disease	2025	Segments enlarged perivascular spaces to derive glymphatic imaging measures
[239]	Deep learning-based denoising for unbiased analysis of morphology and stiffness in amyloid fibrils	2025	Denoises AFM data for unbiased measurement of amyloid fibril morphology and stiffness

Continued on next page

Reference	Title	Year	Reason for Exclusion
[290]	Interpretable deep learning framework for understanding molecular changes in human brains with Alzheimer's disease: implications for microglia activation and sex differences	2025	Interpretable model used to understand molecular changes; output is biological insight
[225]	Probabilistic mapping and automated segmentation of human brainstem white matter bundles	2026	Segments brainstem white matter bundles and builds a probabilistic anatomical map
[88]	MRI-to-PET synthesis via deep learning for amyloid-beta quantification in Alzheimer's disease	2026	Synthesises PET from MRI so amyloid can be quantified without a PET scan
[158]	A cross-modality transformer network for MR-guided low-dose tau PET image denoising	2026	Denoises low-dose tau PET using MR guidance; output is a cleaner image
