# Supplementary material for: From Single-Modal to Multi-Modal Artificial Intelligence in Alzheimer’s Disease: A Systematic Review of Databases, Modalities, Diagnostic Performance, and Clinical Translation Challenges
Source: Sensors (Basel). 2026 Aug 29;26(17):5489. doi: 10.3390/s26175489 (PMC13568021; doi:10.3390/s26175489)
Supplement: Supplementary file 1 [file sensors-26-05489-s001.zip › Table S2.pdf]

Table S2: Summary of the reviewed studies on ML-based classification of AD and related dementias, ordered by data modality (neuroimaging first, followed by EEG/MEG).

Reference	Year	Comparison Groups	Database	Data Type	Best Classifier	Validation	Best Result	Features Used
				**Studies with MRI**				
Beheshti *et al.* [373]	2017	AD vs. HC (102 AD, 99 HC)	Japanese-ADNI (J-ADNI)	MRI	Linear SVM combining MRI measures with Functional Assessment Questionnaire (FAQ)	10-fold cross-validation	AD vs. HC (MRI + FAQ): 97.01% Accuracy	Histogram patterns generated from individual Gray Matter (GM) similarity matrices (raw-feature vectors lexicographically ordered) and Functional Assessment Questionnaire (FAQ) scores.
Aderghal *et al.* [344]	2016	AD vs. MCI vs. NC (188 AD, 399 MCI, 228 NC)	ADNI (1.5T screening)	MRI	2-D+ε CNN approach with reduced data balancing	Random split (60% train, 20% valid, 20% test) split with data augmentation	AD vs. NC: 82.8% Acc. (79.6% sens., 85.9% spec.) Trained with blurred, translated and flipped images (x56); MCI vs. NC: 66.0% Acc. (73.7% sens., 58.7% spec.) Trained with blurred, translated and flipped images (x56); AD vs. MCI: 64.7% Acc. (70.0% sens., 60.0% spec.) Without data augmentation but for the flip (x2)	"2-D+ε" sagittal patches (size 28x28x3) of hippocampal regions. Filter kernels learn gray-value intensity patterns directly from raw hippocampal patches.

Continued on next page

Table S2 – *continued from previous page*

Reference	Year	Comparison Groups	Database	Data Type	Best Classifier	Validation	Best Result	Features Used
Khazaee *et al.* [462]	2016	AD vs. MCI vs. HC (34 AD, 89 MCI, 45 HC)	ADNI	rs-fMRI	SVM with RBF (Radius Basis Function) kernel	Holdout cross-validation (repeated with K=23 from each group)	HC vs. MCI vs. AD (Three-group): 88.42% Acc.; Normal vs. MCI&AD: 87.29% Acc.; AD vs. Normal&MCI: 97.46% Acc.; MCI vs. Normal&AD: 72.03% Acc.	2909 Graph measures: 5 global (clustering coeff, global efficiency, path length, etc.) and 2904 local measures (PageRank/ eigenvector centrality, degree, strength, diversity coeff) across 264 nodes. Key features emphasize Default Mode Network hubs.
Ali *et al.* [498]	2016	AD vs. HC (OASIS dataset)	OASIS	MRI	TANNN	Not specified	99.2% Accuracy on OASIS dataset	Texture and Intensity features: Clusters extracted from Haar wavelet transform representing the "Alzheimer region"; gray values and symmetry measurements of the hippocampus area.
Korolev *et al.* [470]	2016	P-MCI vs. N-MCI (139 Progressive-MCI, 120 Non-progressive MCI)	ADNI-1	MRI, clinical markers, plasma proteomic markers	MKL-Gaussian (multi-source model with 5 Gaussian kernels)	Nested 10-fold stratified cross-validation (repeated 10 times)	P-MCI vs. N-MCI: 80.3% Accuracy (83.4% sens., 76.4% spec., AUC 0.87)	Clinical (CAM): 186 variables (ADAS-Cog, FAQ, RAVLT). MRI Morphometrics: 452 ROI measures (left hippocampus vol, middle temporal and inferior parietal thickness). Plasma (PPM): 149 protein analytes (vWF, E-selectin, Tenascin C, etc.).

Continued on next page

Table S2 – *continued from previous page*

Reference	Year	Comparison Groups	Database	Data Type	Best Classifier	Validation	Best Result	Features Used
Liu *et al.* [485]	2016	AD vs. NC, pMCI vs. NC, pMCI vs. sMCI (97 AD, 128 NC, 117 pMCI, 117 sMCI)	ADNI-1	MRI	Inherent Structure based Multi-view Learning (ISML) ensemble	10-fold cross-validation	AD vs. NC: 93.83% Acc. (92.78% sens, 95.69% spec, AUC 0.94): pMCI vs. sMCI: 80.90% Acc. (85.95% sens., 78.41% spec.)	Multi-view regional GM density maps: D=1500 features per view extracted from 10 AP-selected templates (tissue density maps reflecting local volumetric measurement).
Wang *et al.* [584]	2016	AD vs. HC (28 AD, 98 HC)	OASIS	MRI	WE + MLP + BBO	50 runs of 10-fold stratified cross-validation	92.40% Acc. (92.14% sens., 92.47% spec.)	Wavelet Entropy (WE): 7-element vector calculated from DWT subbands (LL3, HL3, LH3, HH3, HL2, LH2, LL2) using bior4.4 wavelet. Measures disorder/order degree of spatial-frequency resolution.
Cheng *et al.* [383]	2017	AD vs. NC (186 AD, 226 NC); MCI vs. NC (395 MCI, 226 NC); pMCI vs. sMCI (167 pMCI, 228 sMCI)	ADNI	MRI	Multi-Domain Transfer Learning (MDTL = MDTFS + MDTC)	10-fold cross-validation	AD vs. NC: 94.7% Acc. (0.988 AUC); MCI vs. CN: 81.5% Acc. (0.882 AUC); pMCI vs. sMCI : 73.8% Acc. (0.796 AUC)	ROI-based: Grey Matter (GM) tissue volume extracted from 93 ROIs manually labeled via Jacob template. Volumetric combination of GM + White Matter (WM) + CSF from volumetric tissue type extracted from the sMRI images.

Continued on next page

Table S2 – *continued from previous page*

Reference	Year	Comparison Groups	Database	Data Type	Best Classifier	Validation	Best Result	Features Used
Suk *et al.* [563]	2017	AD vs. NC (186 AD, 226 NC); MCI vs. NC (393 MCI, 226 NC); pMCI vs. sMCI (167 pMCI, 226 sMCI)	ADNI	MRI	JLLR + DeepESRNet	10-fold cross-validation	AD vs. NC: 91.02% Acc.,(92.72% Sens., 89.94% Spec., 0.9272 AUC); MCI vs. NC: 73.02% Acc., (77.60% Sens., 68.22% Spec., 0.7361 AUC); pMCI vs. sMCI: 74.82% Acc., (70.93% Sens., 78.82% Spec., 0.7539 AUC)	Regional gray matter (GM) tissue volumes of 93 ROIs based on the Kabani atlas. Also utilizes target-level representations (predicted clinical labels and MMSE/ ADAS-Cog scores) as input for the deep network.
Hon *et al.* [434]	2017	AD vs. HC (100 AD, 100 HC)	OASIS	MRI	Inception V4 (transfer learning)	5-fold cross-validation	AD vs. HC: 96.25% Accuracy	32 most informative axial slices (2D images) per 3D scan, selected by sorting all slices in descending order based on image entropy.
Aderghal *et al.* [345]	2017	AD vs. NC (188 AD, 188 NC); MCI vs. NC (228 MCI, 228 NC); AD vs. MCI (188 AD, 188 MCI)	ADNI (screening)	MRI	Majority Vote Late Fusion	Train-validation split	AD vs. NC: 91.41% Acc., 93.75% Sens., 89.06% Spec.; MCI vs. NC: 66.25% Acc., 65.00% Sens., 66.25% Spec. (Mean Fusion); AD vs. MCI: 69.53% Acc., 71.88% Sens., 67.19% Spec.	Hippocampal ROI patches (28x28x3 units). These represent three 2D slices (low dimension 28x28 pixels) extracted from the 3D volume of the sagittal, coronal, and axial projections.

Continued on next page

Table S2 – *continued from previous page*

Reference	Year	Comparison Groups	Database	Data Type	Best Classifier	Validation	Best Result	Features Used
Li *et al.* [479]	2017	AD vs. NC (199 AD, 229 NC)	ADNI	MRI	Multi-model combination (3D CNN + 3D CAEs)	5-fold cross-validation	AD vs. NC: 88.31% Acc., 91.40% Sens., 84.42% Spec., 92.73% AUC	Generic compact features from whole brain 3D images (69x59x57 input) and multi-scale 3D images downsampled by ratios of 2, 3, and 4 (sizes: 102x88x85, 69x59x57, and 54x44x43); concatenated into a 270-dimensional feature vector.
Korolev *et al.* [471]	2017	AD vs. NC (50 AD, 61 NC); EMCI vs. NC (77 EMCI, 61 NC); LMCI vs. NC (43 LMCI, 61 NC)	ADNI	MRI	ResNet	5-fold cross-validation	AD vs. NC: 80% Acc. (0.87 AUC); EMCI vs. NC: 56% Acc. (0.58 AUC); LMCI vs. NC: 61% Acc. (0.65 AUC); AD vs. EMCI: 63% Acc. (0.67 AUC); AD vs. LMCI: 59% Acc. (0.62 AUC); LMCI vs. EMCI: 52% Acc. (0.52 AUC)	3D MRI voxel intensity values from baseline structural T1-weighted scans, represented as a 3D tensor of shape 110x110x110.
Cheng *et al.* [385]	2017	AD vs. NC (199 AD, 229 NC)	ADNI	MRI	Multi-CNNs ensemble	10-fold cross-validation	AD vs. NC: 87.15% Acc., 86.36% Sens., 85.93% Spec., 92.26% AUC	27 local 3D patches (50x41x40 voxels) extracted from the whole brain structural T1 MRI; most informative patches were located in the hippocampus, parahippocampal gyrus, and amygdala.

Continued on next page

Table S2 – *continued from previous page*

Reference	Year	Comparison Groups	Database	Data Type	Best Classifier	Validation	Best Result	Features Used
Valliani *et al.* [579]	2017	AD vs. CN (188 AD, 229 CN); AD vs. MCI vs. CN (188 AD, 243 MCI, 229 CN)	ADNI	MRI	Pretrained ResNet + augmentation	80/20 train/ test split	AD vs. CN: 81.3% Acc.; 3-way (AD vs. MCI vs. CN): 56.8% Acc.	Median axial slice (single 2D image) extracted from skull-stripped and registered structural T1-weighted MRI scans.
Gunawardena *et al.* [423]	2017	AD vs. MCI vs. NL (Dataset 1: 7 AD, 14 MCI, 15 NL; Dataset 2: 9 AD, 16 MCI, 11 NL)	ADNI	MRI	CNN (Evaluation 3 segmentation)	AD vs. MCI vs. NL: 80/20 split	AD vs. NL: Acc 84.4% (Sens 95.3%, Spec 71.4% via SVM); AD vs. MCI vs. NL: Acc 96% (Sens 96%, Spec 98% via CNN)	2D image intensities: Matrix-flattened 160x160 pixel intensities from coronal slices. Biomarkers: Focus on hippocampal atrophy, ventricle enlargement, and cortex shrinkage. Specific Feature Type: Extended ROI-based intensities without boundary detection (Evaluation 3).
Zhang *et al.* [603]	2016	AD vs. CTL (194 AD, 228 CTL); MCI-C vs. NC (142 MCI-C, 246 MCI-NC); CTL-C vs. NC (39 CTL-C, 73 CTL-NC)	ADNI	MRI	Adaboost (RD+mTBM)	N-fold leave-one-out cross-validation	AD vs. CTL: 81% Acc. (0.78 AUC); MCI-C vs. MCI-NC: 77% Acc. (0.75 AUC); CTL-NC vs. CTL-C: 71% Acc. (0.67 AUC)	Hippocampal Surface Morphometry: 4x1 vectors per vertex on meshes of 15,000 points (150x100). Composed of radial distance (RD) and 3 independent mTBM components.

Continued on next page

Table S2 – *continued from previous page*

Reference	Year	Comparison Groups	Database	Data Type	Best Classifier	Validation	Best Result	Features Used
Hosseini-Asl *et al.* [436]	2016	AD vs. MCI vs. NC (70 AD, 70 MCI, 70 NC)	CADDementia (source domain) and ADNI (target domain)	MRI	3D Adaptable CNN (3D-ACNN)	10-fold cross-validation	AD vs. NC: 97.6% Acc.; AD vs. MCI: 95.0% Acc.; MCI vs. CN: 90.8% Acc.	Hierarchical 3D feature maps: Capture anatomical shape variations including cortex thickness, brain size (gender-related), ventricle size, and hippocampus models. Map dimensions: (102x76x76), (52x40x40), (28x22x22).
Suk *et al.* [562]	2016	MCI vs. NC (ADNI2: 31 MCI, 31 NC; In-house: 12 MCI, 25 NC)	ADNI-2 and In-house	rs-fMRI	Deep Auto-Encoder (DAE) + Hidden Markov Model (HMM)	Leave-one-out cross-validation	NC vs. MCI: 81.08% Acc. (77.78% sens., 82.14% spec.) (In-House)	Regional Time Series: Mean time series of 116 ROIs (AAL template) transformed by DAE into low-dimensional encoded representations of complex non-linear functional networks.
Wang *et al.* [585]	2017	MCI vs. HC (400 MCI, 229 HC; 8000 images after augmentation)	ADNI	MRI	Expedited CNN (with Tucker decomposition)	62,5/ 18,75/ 18,75 train/ test/ validation	MCI vs. HC: Acc 90.6%, Recall (Sens) 92.8% (F-score 89.4%)	Deep Image Features: Representative features automatically extracted via a 9-layer architecture (5 convolutional/pooling and 2 fully connected layers). Input: Augmented and pre-trained image intensity representations from serial MRI scans.

Continued on next page

Table S2 – *continued from previous page*

Reference	Year	Comparison Groups	Database	Data Type	Best Classifier	Validation	Best Result	Features Used
Zhang *et al.* [604]	2018	AD vs. HC (98 AD, 98 HC)	OASIS and Local Hospitals	MRI (MP-RAGE)	SNN trained by PP-PSO	10-fold cross validation repeated 50	AD vs. HC: Acc 92.73% (Sens 92.69%, Spec 92.78%, AUC 0.95)	Texture Features (13): Information entropy values derived from 13 sub-bands generated by a 4-level Stationary Wavelet Transform (SWT). These features are "translation invariant," capturing the degree of order/disorder in gray-level fluctuations caused by hippocampal shrinkage.
Tong *et al.* [569]	2017	Training: AD vs. NC (191 AD, 229 NC); Testing: PMCI vs. SMCI (171 PMCI, 129 SMCI)	ADNI	MRI	Combined biomarkers (Grading + Cognitive) with RF	10-fold	PMCI vs. SMCI: AUC 91.7%, Acc 84.1% (Sens 88.7%, Spec 76.5%); AD vs. NC: Acc 88% (at 5mm registration)	Primary Biomarker: "Global Grading" value (-1 to 1) representing similarity to AD vs. NC groups, calculated via sparse representation of voxel intensities. Cognitive Measures: MMSE, CDR-SB, RAVLT, FAQ, and ADAS-cog. Other: Age as a predictive risk factor.

Continued on next page

Table S2 – *continued from previous page*

Reference	Year	Comparison Groups	Database	Data Type	Best Classifier	Validation	Best Result	Features Used
Wang *et al.* [587]	2018	MCI-C vs. MCI-NC (101 Converters, 115 Non-converters)	ADNI	MRI	Temporal-GAN	five repetitions of random data division (10%/ 20%/50% test splits).	MCI Conversion (cMCI vs. sMCI): 78.18% Acc. (10% test split)	Longitudinal Gray Matter (GM) volumes normalized by total intracranial volume. Features were extracted from 93 ROIs across 6 longitudinal time points: baseline (BL), month 6, 12, 18, 24, and 36. The regression network learns to predict volume changes (xt to xt+1) to uncover temporal correlation structures.
Kamathe *et al.* [539]	2017	AD vs. MCI vs. NC (26 AD, 68 MCI, 107 NC)	OASIS	MRI (Axial)	AdaBoost	K-fold CV (k=15)	AD vs. NC: 100% Acc; AD vs. MCI: 100% Acc; MCI vs. NC: 90.28% Acc; Abnormal vs. Normal: 87% Acc	6 Robust optimized Gray Level Co-occurrence Matrix (GLCM) features: Contrast, Correlation, Energy, Homogeneity, Information Measure of Correlation, and Absolute Value. These were selected from an initial set of 11 Haralick texture features extracted from axial T1-MRI slices.

Continued on next page

Table S2 – *continued from previous page*

Reference	Year	Comparison Groups	Database	Data Type	Best Classifier	Validation	Best Result	Features Used
Islam *et al.* [444]	2018	AD Multi-class (Non-demented, Very mild, Mild, Moderate AD) (416 total)	OASIS	MRI	Ensemble (M1+M2+M3)	80/20 train/ test split (10% of train for validation)	Multi-class (4-stage): Acc 93.18%, Precision 94%, Recall (Sens) 93%, F1 92%.	Spatial Patches: 2D image patches (size 112×112) extracted from three planes: axial (horizontal), coronal (frontal), and sagittal (median). Hierarchical: Multi-level representations learned directly from input sMRI, capturing latent hidden disease patterns.
Bäckström *et al.* [366]	2018	AD vs. NC (199 AD, 141 NC)	ADNI	MRI	Proposed 3D ConvNet	60/20/20 train/val/ test split	AD vs. NC: Acc 98.74%, SEN (AD detection) 100%, SPE (NC classification) 97.56%.	Global Brain: The entire 3D brain scan volume normalized to 110×110×110 size. Structural Atrophy: Automatically learned features describing hippocampal/ cerebral cortex shrinkage and enlargement of the ventricles.

Continued on next page

Table S2 – *continued from previous page*

Reference	Year	Comparison Groups	Database	Data Type	Best Classifier	Validation	Best Result	Features Used
Altaf *et al.* [358]	2018	AD vs. Normal vs. MCI (92 AD, 90 Normal, 105 MCI)	ADNI	MRI, clinical features	Multi-class: KNN; Binary (AD/NC): SVM/Ensemble	5-fold cross-validation (80/20 train-test split)	AD vs. Normal: 98.9% Acc. (CSF portion, GM + WM + CSF); Normal vs. MCI: 91.8% Acc. (Whole image); AD vs. MCI: 85.3% Acc. (Whole image); Multi-class (3-way): 79.8% Acc. (Whole image).	Hybrid Texture and Clinical Vector: (1) Texture (from whole and segmented images): 11 GLCM statistics (contrast, correlation, homogeneity, entropy, etc.) averaged over slices (interval of 10); SIFT key points; HOG (8x8 cell size); and LBP (16x16 neighborhood). (2) Behavioral/Clinical data: Functional Activities Questionnaire (FAQ), Neuropsychiatric Inventory (NPI), and Geriatric Depression Scale (GDS) scores.
Lin *et al.* [484]	2018	MCIc vs. MCInc (169 MCIc, 139 MCInc); AD vs. NC (188 AD, 229 NC)	ADNI	MRI	ELM (CNN + FreeSurfer features)	LOOCV	MCIc vs. MCInc (3-yr conversion): Acc 81.4%, AUC 87.8%, SEN 89.6%, SPE 68%; AD vs. NC (Baseline): Acc 88.79%.	CNN-based: 1024 high-level features from 151 2.5D patches per subject (axial/coronal/sagittal) centred on the hippocampus. Morphological: 325 features including cortical volume, surface area, and thickness average/SD. Top ROI: average thickness of Left FrontalPole.

Continued on next page

Table S2 – *continued from previous page*

Reference	Year	Comparison Groups	Database	Data Type	Best Classifier	Validation	Best Result	Features Used
Liu *et al.* [487]	2018	AD vs. NC (199 AD, 229 NC); pMCI vs. sMCI (167 pMCI, 226 sMCI)	ADNI-1, ADNI-2, MIRIAD	MRI (1.5T and 3T)	LDMIL-G (with transferred knowledge)	Independent testing (Train ADNI-1, Test ADNI-2/ MIRIAD)	AD vs. NC (ADNI-2): Acc 91.09%, AUC 0.959. AD vs. NC (MIRIAD): Acc 92.75%, AUC 0.972. pMCI vs. sMCI: Acc 76.90%, AUC 0.776. pMCI vs. sMCI (Guided): Acc 78.34%, AUC 0.790.	Local: Bag-of-patches (size 24×24×24) from 40 discovered anatomical landmarks (e.g., bilateral hippocampal, parahippocampal, and fusiform areas). Global: Bag-level representations capturing complex relational patterns between all landmarks.
Senanayake *et al.* [545]	2018	AD vs. MCI vs. CN (161 AD, 193 MCI, 161 CN)	ADNI	MRI, neuropsychological measures (NM)	Deep fusion pipeline	85/15 train/ test split	CN vs. All: Acc ∼83%. MCI vs. All: Acc ∼82%. CN vs. MCI: Acc ∼75%. MCI vs. AD: Acc ∼77%. CN vs. AD: Acc ∼79%.	Neuroimaging: 3D MR volumes reduced from 10 million voxels to comparable feature dimensions. Cognitive: 35 unidimensional features based on neuropsychological measure (NM) vectors.
Cui *et al.* [389]	2018	AD vs. Normal Control (198 AD, 229 NC)	ADNI	MRI	MLP + Stacked Bidirectional GRU (BGRU)	5-fold cross-validation	AD vs. NC: 89.69% ACC (86.87% sens., 92.58% spec.).	Spatial and Longitudinal Features: Gray Matter (GM) density maps (downsampled to 49x41x40). MLP-extracted features: 300 spatial intermediate feature vectors per single time point. Longitudinal features: Sequence of feature vectors learned across multiple time points (t0 to t4) using stacked BGRU.

Continued on next page

Table S2 – *continued from previous page*

Reference	Year	Comparison Groups	Database	Data Type	Best Classifier	Validation	Best Result	Features Used
Alickovic *et al.* [354]	2020	AD vs. Normal Control (72 AD, 195 CN)	ADNI	MRI (1.5T)	Random Forest (Best overall ACC); k-NN (Best SEN)	10-fold cross-validation	AD vs. CN: 85.77% ACC (54.17% sens., 97.44% spec.).	10-bin normalized histogram feature vectors: Represents the probability estimation of brain gray level appearance within a range of digital gray levels (e.g., 0 to M-1).
Rieke *et al.* [534]	2018	AD vs. NC (193 AD, 151 NC subjects; total 969 scans)	ADNI	MRI	3D CNN	5-fold CV (patient-level split)	AD vs. NC: 77% Acc, 0.78 AUC	Voxel-wise intensity values from full-brain structural 3D T1-MRI scans. Features are represented as standardized volumes of 193 × 229 × 193. The 3D CNN learns meaningful internal representations directly from these normalized voxel intensities instead of relying on hand-crafted features.
Taqi *et al.* [567]	2018	AD vs. NC (250 AD, 250 NC)	ADNI	MRI (T2)	TF-CNN with RMSProp optimizer	Holdout validation/test split (0.16)	AD vs. NC: 100% Acc	Augmented 2D axial T2-MRI slices (1200 total training images). Original raw image resolution was 2048 x 2048, cropped down to 1740 × 1740 pixels. The 3D CNN extracts high-order features designed to be rotation-invariant based on the augmented dataset.

Continued on next page

Table S2 – *continued from previous page*

Reference	Year	Comparison Groups	Database	Data Type	Best Classifier	Validation	Best Result	Features Used
Wu *et al.* [593]	2018	sMCI vs. cMCI vs. NC (150 sMCI, 157 cMCI, 150 NC)	ADNI	MRI	CaffeNet	5-fold CV (66.6/33.3 Train/Test)	NC identification: 98.71% Acc; cMCI identification: 92.35% Acc; sMCI identification: 72.04% Acc; 3-way (sMCI/cMCI/NC): 87.78% Acc; MCI Conversion Risk: 97.01% Acc	Synthesized multi-view 3-channel RGB color images. These were created by combining 3 specific slices from the 3D T1-MRI volume at an interval of 4 to meet 3-channel CNN requirements. 16 such synthesized RGB images were generated per subject, resized to 256×256 pixels.
Bi *et al.* [375]	2018	AD vs. HC (25 AD, 36 HC)	ADNI	rs-fMRI (3.0T)	Random Elman NN Cluster	Ensemble majority voting (8:2 training/ test split)	AD vs. HC: 92.31% Acc.	Functional Connectivity: 4,005 Pearson correlation coefficients between 90 AAL brain regions. 180 optimal features selected. 23 highest-weight abnormal regions identified, including Precentral Gyrus (PreCG), Olfactory Cortex (OLF), Superior Frontal Gyrus (ORBsup), and Middle Frontal Gyrus (MFG).

Continued on next page

Table S2 – *continued from previous page*

Reference	Year	Comparison Groups	Database	Data Type	Best Classifier	Validation	Best Result	Features Used
Zheng *et al.* [609]	2018	NC vs. AD vs. MCI (165 NC, 142 AD, 221 MCI); sMCI vs. pMCI (95 sMCI, 126 pMCI)	ADNI	MRI	MFN combined with Morphological Features and Network Properties using SVM	Leave-one-out cross-validation (LOOCV) for primary results. Reliability further tested via N-fold cross-validation (N=5, 7, 10) repeated 30 times.	Best results achieved by combining MFN + MF + NP: NC vs. AD: 98.70% Acc (0.9989 AUC); NC vs. MCI: 97.93% Acc (0.9934 AUC); MCI vs. AD: 73.82% Acc (0.7731 AUC); sMCI vs. pMCI: 67.42% Acc (0.7213 AUC)	Total Multi-modal Features: 6 Morphological Features (MF): Cortical thickness (CT), surface area (SA), volume (VOL), local gyrification index (LGI), sulcal depth (SD), and gyrus height (GH) across 78 AAL regions. Network Connections (MFN): 6,006 binary asymmetric cross-regional relationships. Network Properties (NP): 391 features including Clustering Coefficient (CC), global efficiency, degree (in/out/in-out), and average shortest path length.

Continued on next page

Table S2 – *continued from previous page*

Reference	Year	Comparison Groups	Database	Data Type	Best Classifier	Validation	Best Result	Features Used
Liu *et al.* [488]	2018	AD vs. MCIc vs. MCInc vs. HC (200 AD, 120 MCIc, 160 MCInc, 230 HC)	ADNI	MRI	WBHN with MKBoost (C3)	10-fold cross-validation repeated 100 times	AD vs. HC: 94.65% Acc. (AUC 0.954). AD vs. MCI: 89.63% Acc. (AUC 0.907). MCI vs. HC: 85.79% Acc. (AUC 0.826). MCIc vs. MCInc: 72.08% Acc. (AUC 0.716)	Texture-based Connectivity: 12,561 Pearson correlations between pairs of ROIs. ROIs extracted across a 4-layer hierarchy: 90 cerebral ROIs (L4), 54 merged ROIs (L3), 14 limbic/systemic ROIs (L2), and 1 whole-brain ROI (L1) based on AAL. Texture parameters: Energy, Contrast, Inverse Difference Moment, Entropy, Difference Variance, and Difference Entropy.
Zhou *et al.* [610]	2018	AD vs. MCI vs. NC (177 AD, 245 MCI, 85 NC); Local (18 AD, 18 NC)	ADNI and Local Hospital	MRI (1.5T)	TrAdaboost (Transfer Learning)	Train/Test with	ADNI to Local Hospital (AD vs. NC): 93.75% Acc (100% Spec). AD to MCI (Transfer): 85.4% Acc	Anatomical Volume of Interest (AVOI): Gray matter volume in 90 AAL regions. Reduced to 7 common discriminative features: L/R Hippocampus, L/R Amygdala, R Parahippocampal gyrus, L Angular gyrus, and Inferior temporal gyrus.

Continued on next page

Table S2 – *continued from previous page*

Reference	Year	Comparison Groups	Database	Data Type	Best Classifier	Validation	Best Result	Features Used
Spasov *et al.* [41]	2019	AD vs. HC (192 AD, 184 HC); pMCI vs. sMCI (181 pMCI, 228 sMCI)	ADNI	MRI (MP-RAGE), tabular clinical data	Parameter-efficient CNN (Dual-learning multi-tasking approach)	10-fold cross-validation; Train/ validation/ test split: 680/ 72/32 subjects	AD vs. HC: 100% Acc., (100% Sen., 100% Spe., 1.0 AUC); pMCI vs. sMCI: 86% Acc., (87.5% Sen., 85% Spe., 0.925 AUC)	Multi-modal inputs: Combines sMRI images and Jacobian Determinant (JD) maps (quantifying local volumetric warp changes). Includes demographic data (age, gender, ethnicity, education), neuropsychological assessment scores (CDRSB, ADAS11, ADAS13, RAVLT), and APOe4 genotyping (genetic status).
Ayadi *et al.* [364]	2019	DS-66 (18 healthy, 48 pathological); DS-160 (20 healthy, 140 pathological); DS-255 (35 healthy, 220 pathological)	Harvard Medical School (DS-66, DS-160, DS-255)	MRI (T2, axial)	SVM with Radial Basis Function (RBF) kernel	10 repetitions of k-fold stratified CV (6-fold for DS-66, 5-fold for DS-160/255)	DS-66/DS-160: 100% Acc., (100% Sen., 100% Spe.); DS-255: 99.61% Acc., (100% Sen., 97.14% Spe.)	Visual Words: Informative histogram representation of clustered descriptors from SURF (Speed Up Robust Features) and Discrete Wavelet Transform (DWT) coefficients. Uses an experimentally determined dictionary size of 300 visual words.

Continued on next page

Table S2 – *continued from previous page*

Reference	Year	Comparison Groups	Database	Data Type	Best Classifier	Validation	Best Result	Features Used
Böhle *et al.* [378]	2019	AD vs. HC (193 AD patients, 151 healthy controls)	ADNI1	MRI (MP-RAGE 1.5T)	CNN with Layer-wise Relevance Propagation (LRP)	Patient-level split: 797 images (train), 100 images (validation), 172 images (test)	AD vs. HC: 87.96% Acc.	Voxel-wise relevance: Individual relevance "fingerprints" showing positive contributions to AD classification. High-importance features concentrate in the medial temporal lobe, including the Hippocampus, Amygdala, Parahippocampal gyrus, and Entorhinal cortex, as well as frontal and parietal areas.
Basheera and Sai Ram [370]	2019	AD vs. MCI vs. CN (635 AD, 548 MCI, 637 CN; 1820 total MRI volumes)	ADNI	MRI (T2, axial)	CNN using segmented Gray Matter (GM)	80:20 Training/ Test split (e.g., multiclass: 14,414 slices train, 3603 slices test)	AD vs. CN: 100% Acc., (100% Sen., 100% Spe., 100% AUC); AD vs. MCI: 96.2% Acc., (100% Sen., 93% Spe., 98.72% AUC); CN vs. MCI: 98.0% Acc., (100% Sen., 96% Spe., 99.87% AUC); Multiclass: 90.47% Acc., (86.66% Sen.)	Segmented Gray Matter (GM): Voxel-level intensity and texture changes in the gray matter tissue. Features focus on tissue atrophy visible in 18,017 segmented voxels/axial slices extracted from MRI volumes.

Continued on next page

Table S2 – *continued from previous page*

**Reference**	**Year**	**Comparison Groups**	**Database**	**Data Type**	**Best Classifier**	**Validation**	**Best Result**	**Features Used**
Liu *et al.* [490]	2019	AD vs. pMCI vs. sMCI vs. NC (ADNI-1: 181 AD, 226 NC, 165 pMCI, 225 sMCI; ADNI-2: 143 AD, 185 NC, 37 pMCI, 234 sMCI; MIRIAD: 46 AD, 23 NC; AIBL: 72 AD, 447 NC)	ADNI-1, ADNI-2, MIRIAD, and AIBL	MRI, demographic data	Deep multi-task multi-channel convolutional neural network	Independent validation: Model trained on ADNI-1 and tested on ADNI-2, MIRIAD, and AIBL datasets	AD vs. NC (MIRIAD): 93.7% Acc., (94.6% Sen., 93.2% Spe., 0.986 AUC); Multi-class (ADNI-2): 51.8% Overall Acc.	Landmark-based patches: Combination of 50 local 3D patches (24x24x24) centred at discriminative landmarks in the bilateral hippocampus, parahippocampus, and fusiform. Includes demographic factors (age, gender, and education) explicitly embedded in fully connected layers.
Acharya *et al.* [341]	2019	AD vs. Normal (Clinical: 55 AD, 110 Normal; Benchmark: 30 AD, 30 Normal)	Clinical (Univ. Malaya Medical Centre) and Benchmark (Harvard Brain Atlas)	MRI (T2, axial)	ST + KNN (Shearlet Transform with KNN classifier)	3-fold stratified Cross Validation (CV)	Benchmark: 98.48% Acc., (96.97% Sen., 100% Spe.).; Clinical: 94.54% Acc., (96.30% Sen., 93.64% Spe.)	Energy/Entropy Features: Vital multi-resolution features derived from ST decomposition. Primary features include S1O1_Vertical_Fuzzy Entropy, S2O5_Vertical_Yager Entropy, S1O9_Horizontal_Vadja Entropy, and Horizontal Energy features.

Continued on next page

Table S2 – *continued from previous page*

Reference	Year	Comparison Groups	Database	Data Type	Best Classifier	Validation	Best Result	Features Used
Yue *et al.* [602]	2019	AD vs. MCI vs. NC (335 AD, 542 MCI, 785 NC); NC vs. EMCI vs. LMCI vs. AD (1106 NC, 1583 EMCI, 1304 LMCI, 366 AD)	ADNI-1 and ADNI-2	MRI	VHFE (Voxel-based Hierarchical Feature Extraction) with Kendall's rank correlation and CNN	5-fold CV (10 repeated tests); 20% independent test set	AD vs. NC (ADNI-1): 99.70% Acc. (100.0% sens., AUC 0.97); AD vs. MCI (ADNI-1): 97.80% Acc. (98.4% sens., AUC 0.80); NC vs. MCI (ADNI-1): 98.90% Acc. (98.5% sens., AUC 0.90); AD vs. EMCI (ADNI-2): 100% Acc. (100.0% sens., 100.0% spec); AD vs. LMCI (ADNI-2): 99.7% Acc. (99.7% sens., 99.7% spec)	Voxel Intensities: Informative intensity values from Gray Matter (GM) volumes. Regional Selection: 50 most informative ("irrelevant" inter-group correlation) voxels selected from each of the 90 anatomical ROIs defined by the AAL template (excluding cerebellum).
Ji *et al.* [449]	2019	AD vs. MCI vs. NC (179 AD, 254 MCI, 182 NC)	ADNI	MRI	eEnsemble (Ensemble of end-to-end trained base classifiers)	3:1:1 split (60% train, 20% validation, 20% test)	AD vs. MCI: 97.65% Acc. (96.00% sens., 100.00% spec., AUC 1.00); MCI vs. NC: 88.37% Acc. (80.56% sens., 94.00% spec., AUC 0.96); AD vs. NC: 98.59% Acc. (97.22% sens., 100.00% spec.)	Brain Slices: 2D Image slices (224×224 pixels) in TIF format. Tissue Selection: 20 consecutive slices per subject identified as having the most significant brain structure from both Gray Matter (GM) and White Matter (WM) volumes.

Continued on next page

Table S2 – *continued from previous page*

Reference	Year	Comparison Groups	Database	Data Type	Best Classifier	Validation	Best Result	Features Used
Ezzati *et al.* [403]	2019	AD vs. MCI vs. CN (249 AD, 656 MCI, 424 CN)	ADNI	MRI, clinical data	Ensemble Linear Discriminant (ELD)	10-fold CV for training; Independent test set (MCI group)	CN vs. AD: 92.8% Acc. (95.8% sens., 88.3% spec., AUC 0.96); MCI to AD progression (48m): 77.0% Acc.(59.6% sens., 86.1% spec.)	Demographics: Age, sex, and education. Genetics: APOE4 carrier status (at least one $\varepsilon 4$ allele). MRI Volumetrics: Normalized volumetric measures (ROIv/TICV) of 47 cortical and subcortical regions parcellated by FreeSurfer, with specific focus on hippocampal volume.
Wang *et al.* [588]	2019	AD vs. MCI vs. Normal (221 AD, 297 MCI, 315 Normal)	ADNI	MRI (3D T1-weighted)	Probability-based ensemble 3D-DenseNet	10-fold cross-validation repeated 10 times (10 equal-sized subsamples, 9 subsamples are used as training data)	AD vs. Normal: 98.83% ACC (98.7% sens., 98.7% spec.). MCI vs. Normal: 98.42% ACC (98.34% sens., 98.37% spec.). AD vs. MCI: 93.61% ACC (92.45% sens., 94.59% prec.). AD vs. MCI vs. Normal: 97.52% ACC.	3D structural feature volumes (91x109x91) automatically extracted by 3D convolution kernels (3x3x3). The ensemble method utilizes the class probabilistic scores from multiple networks to determine the final label.

Continued on next page

Table S2 – *continued from previous page*

Reference	Year	Comparison Groups	Database	Data Type	Best Classifier	Validation	Best Result	Features Used
Basaia *et al.* [369]	2019	AD vs. c-MCI vs. s-MCI vs. HC (418 AD, 280 c-MCI, 533 s-MCI, 407 HC)	ADNI + Milan dataset	MRI (3D T1-weighted)	3D CNN (specifically "All Convolutional Network")	10-fold cross-validation (augmented training set); 10% independent test set	AD vs. HC (ADNI): 99.2% ACC (98.9% sens., 99.5% spec.). c-MCI vs. HC (ADNI+Milan): 87.7% ACC (87.3% sens., 88.1% spec.). s-MCI vs. HC (ADNI+Milan): 76.4% ACC (75.1% sens., 77.8% spec.). AD vs. c-MCI (ADNI+Milan): 75.8% ACC (74.8% sens., 77.1% spec.). AD vs. s-MCI (ADNI+Milan): 86.3% ACC (84.0% sens., 88.7% spec.). c-MCI vs. s-MCI (ADNI): 75.1% ACC (74.8% sens., 75.3% spec.).	3D Gray Matter (GM) tissue probability maps in MNI space, normalized and modulated. The network automatically extracts an optimal representation from these maps without manual feature engineering.
Kar *et al.* [458]	2019	AD vs. Healthy Control (9 AD, 11 HC)	ADNI	DTI, MRI/CT	Neuro-Fuzzy classification system	Training (14 subjects), Validation (3), Testing (3)	AD vs. HC: 100% ACC (100% sens., 100% spec.).	DTI/MRI Measures: Apparent Diffusion Coefficient (ADC) (quantifies preferred water diffusion restriction); Fractional Anisotropy (FA) (indicates degree of anisotropy in white matter tracts, values 0 to 1); Gray Matter (GM) volume (measured in brain voxels).

Continued on next page

Table S2 – *continued from previous page*

Reference	Year	Comparison Groups	Database	Data Type	Best Classifier	Validation	Best Result	Features Used
Vaithinathan *et al.* [578]	2019	AD vs. NC (189 AD, 227 NC); cMCI vs. MCInc (165 cMCI, 231 MCInc); AD vs. MCI (189 AD, 396 MCI); NC vs. MCI (227 NC, 396 MCI)	ADNI	MRI	k-nearest neighbors (kNN) with Fisher selection	Training/ testing split (AD vs. NC: 93/93 train, 96/134 test) with bootstrapped feature selection and k-fold CV	AD vs. NC: 87.39% Acc., (89.58% Sen., 85.82% Spe).; AD vs. MCI: 63.41% Acc., (57.29% Sen., 65.35% Spe.); NC vs. MCI: 64.74% Acc.,(45.61% Sen., 72.44% Spe.); cMCI vs. MCInc: 66.38% Acc.,(60.24% Sen., 69.80% Spe.)	2D textures from 3D MRI: Mathematically quantified variations extracted per block on slices 11–50 of all axes (N_b=9,15,21). Key features include Central moments, Homogeneity, Contrast, Inverse difference moment, and Entropy. ROIs involve focal atrophy patterns in the Hippocampus (39% contribution), Thalamus (27%), Amygdala (14%), Middle temporal gyrus (11%), and Insula (5%).
Shmulev *et al.* [553]	2018	MCI converters vs. MCI stable (327 cMCI, 532 sMCI)	ADNI	MRI, clinical data	XGBoost (Clinical + Embedding data)	5-fold group cross-validation	cMCI vs. sMCI (Conversion): Acc 76%, AUC 0.86, sen 88%, spec 70%.	Clinical: recruitment data, demographics (age, gender, education), physical exams, cognitive assessments (MMSE, etc.), genetic (APOE), and biospecimen markers (CSF, blood, urine). Neuroimaging: 64-dimensional vector embedding learned via ResNet3D with Histogram loss.

Continued on next page

Table S2 – *continued from previous page*

Reference	Year	Comparison Groups	Database	Data Type	Best Classifier	Validation	Best Result	Features Used
Qiu *et al.* [530]	2018	NC vs. MCI (303 NC, 83 MCI)	NACC	MRI (T1-weighted or FLAIR, axial)	Multi-modal fusion model (combined three base models using majority voting)	2:1:1 train/val/test split; 5 random splits	NC vs. MCI: Acc 90.9%, AUC 0.83–0.84 (MRI base models), Recall (Sens) 96.3%, Precision 92.6%.	MRI: Three axial slices (signature + 2 adjacent) covering lateral ventricles, inferior temporal, and middle temporal cortices. MMSE: Orientation (time), orientation (place), and total MMSE score. LM Test: delayed recall units, elapsed time, and total story units recalled.
Toshkhujaev *et al.* [571]	2020	AD vs. HC (81 AD, 171 HC); EMCI vs. LMCI (39 EMCI, 35 LMCI)	GARD (NRCD), NACC, ARWIBO, ADNI	MRI	MALPEM Segmentation + PCA + RBF-SVM	10-fold stratified K-fold (SKF) cross-validation	AD vs. HC: 97.37% Acc. (98.18% sens., 95.24% spec., AUC 0.975) (NRCD, using Cortical thickness); EMCI vs. LMCI: 95.45% Acc. (92.75% sens., 100% spec., AUC 0.964) (NRCD, using Subcortical volume)	138 Anatomical Morphometry features: 40 subcortical volumes (e.g., hippocampus, amygdala) and 98 cortical thickness measures. Features were reduced via PCA, with the first principal component accounting for 96% of the variance.

Continued on next page

Table S2 – *continued from previous page*

Reference	Year	Comparison Groups	Database	Data Type	Best Classifier	Validation	Best Result	Features Used
Gnanasegar *et al.* [419]	2020	Demented vs. Non-Demented (146 Demented, 146 Non-demented, 37 Converted)	OASIS	MRI (numerical preprocessed features)	Boruta Feature Selection + LSTM	80/10/10 train-validate-test split	Demented vs. Non-Demented: 94% accuracy on independent test set	Clinical and derived imaging measures: MMSE score, CDR (Clinical Dementia Rating), nWBV (Normalized Whole-brain Volume), MR Delay, SES, eTIV, ASF, Age, Gender, and Education. CDR and MMSE were ranked by Boruta as the top informative features.
Lian *et al.* [483]	2020 (Print)	AD vs. NC (199 AD, 229 NC); pMCI vs. sMCI (167 pMCI, 226 sMCI)	ADNI-1 and ADNI-2	MRI (1.5T and 3.0T)	wH-FCN (Hierarchical Fully Convolutional Network)	Independent training (ADNI-1) and testing (ADNI-2); Subject-level diagnosis	AD vs. NC: 90.3% Acc. (AUC 0.951); pMCI vs. sMCI: 80.9% Acc. (AUC 0.781); (Both using wH-FCN with automated discriminative localization)	Multi-scale feature representations: 120 patch locations (25x25x25 size) automatically localized. Yields hierarchical features at the patch-level (1x1x1x64), region-level, and subject-level. Atrophy localized at the ventricle, hippocampus, and fusiform gyrus.
Tufail *et al.* [573]	2020	AD vs. CN (90 AD, 90 CN); AD vs. CN (30 AD, 84 CN); AD vs. CN (100 AD, 100 CN)	OASIS-1; Hon *et al.*	MRI (cross-sectional)	Xception (Dataset-1 & 2); Inception v3 (Dataset-3)	2, 5, 6, and 9-fold cross-validation	AD vs. CN: 99.45% Acc (Inception v3, Dataset-3, 9-fold); AD vs. CN: 82.79% Acc (Custom CNN, Dataset-2, 5-fold); AD vs. CN: 64.87% Acc, 64.88% Sens, 64.86% Spec (Xception, Dataset-1, 9-fold)	96 Informative slices per subject visually selected (first/last 40 ignored). Features represent automated feature learning from local brain images identifying anatomical changes such as focal lesions and gray matter loss in the lobes.

Continued on next page

Table S2 – *continued from previous page*

Reference	Year	Comparison Groups	Database	Data Type	Best Classifier	Validation	Best Result	Features Used
Battineni *et al.* [371]	2020	Demented vs. Non-demented (146 D, 190 ND, 37 Converted)	OASIS longitudinal MRI data	MRI (longitudinal)	Logistic Regression (LR)	10-fold cross-validation	D vs. ND: 98.3% Acc, 98.6% sens, 97.4% Recall, 0.997 AUC; D vs. ND: 97.6% Acc, 98.2% Prec, 98.3% sens, 0.996 AUC (KNN)	14 Input features: Age, Gender, Education (EDUC), Social-economic status (SES), MMSE, CDR, estimated total intracranial volume (e-TIV), normalized whole brain volume (n-WBV), and atlas scaling factor (ASF). Reduced via PCA to highly correlated components.
Kumari *et al.* [472]	2020	AD vs. cMCI vs. ncMCI vs. CN (200 total images; 100 Train, 100 Test)	OASIS-3	MRI	CNN	50/50 train/test split	Multiclass: 90.25% Acc, 85.53% sens	13 Textural features extracted via GLCM: contrast, correlation, energy, entropy, homogeneity, standard deviation, skewness, kurtosis, variance, Mean, RMS, IDM, and Smoothness.
Tang *et al.* [566]	2021	AD vs. LMCI vs. EMCI vs. CN (80 AD, 110 LMCI, 230 EMCI, 140 CN)	ADNI	MRI (structural)	Random Forest (RF)	10-fold cross-validation	CN vs. AD: 96.14% Acc, 88.14% Sens, 92.81% Spec, 0.92 AUC; EMCI vs. AD: 90.15% Acc, 93.51% Sens, 92.43% Spec, 0.85 AUC; LMCI vs. AD: 84.54% Acc, 0.89 AUC; CN vs. EMCI: 77.45% Acc, 0.59 AUC	272 original MRI attributes reduced via statistical dimensionality reduction to 5 key MRI indexes: Subcutaneous volume (SV), Surface area (SA), Cortical volume (CV), Thickness of cortical area (TA), and Hippocampal subfield (HS). Brain atrophy is measured in mm, mm^2, and mm^3.

Continued on next page

Table S2 – *continued from previous page*

Reference	Year	Comparison Groups	Database	Data Type	Best Classifier	Validation	Best Result	Features Used
Salunkhe *et al.* [542]	2021	AD vs. CN (119 AD, 115 CN)	ADNI	MRI (3T MPRAGE)	Ensemble Bagged Trees	70/30 train/ test split; 5-fold cross-validation during training	AD vs. CN: 90.2% Acc, 84.9% Recall, 88.7% Spec, 0.92 AUC (Textural + Structural); AD vs. CN: 87.2% Acc (SVM, Structural only)	20 GLCM textural features (top 8 significant: Autocorrelation, Energy, Entropy, Homogeneity, Max Prob, Correlation, Info Measures of Correlation 1 & 2); Structural features: Hippocampus volume (Average, CA1-CA4, Subiculum, etc.) and Patient Age.
Pan *et al.* [521]	2020	AD vs. HC (137 AD, 162 HC); MCIc vs. HC (76 MCIc, 162 HC); MCIc vs. MCInc (76 MCIc, 134 MCInc)	ADNI	MRI	CNN-EL (Classifier ensemble based on three-axis slices)	Stratified 5-fold cross-validation repeated 10 times	AD vs. HC: 0.84 Acc, 0.92 AUC, 0.68 MCC; MCIc vs. HC: 0.79 Acc, 0.83 AUC, 0.49 MCC; MCIc vs. MCInc: 0.62 Acc, 0.59 AUC, 0.10 MCC	123 informative slices (40 sagittal, 50 coronal, 33 transverse). Intersection points from most discriminable slices map to Brainnetome Atlas regions, primarily identifying the Medial Temporal Lobe, Entorhinal Cortex, and Caudal Hippocampus.

Continued on next page

Table S2 – *continued from previous page*

Reference	Year	Comparison Groups	Database	Data Type	Best Classifier	Validation	Best Result	Features Used
Bloch *et al.* [377]	2021	sMCI (400) vs. pMCI (319)	ADNI, AIBL (External)	MRI (baseline visit)	XGBoost (with Data Shapley)	Binary classification (65% train, 15% validation, 20% test)	sMCI vs. pMCI (ADNI): 68.89% Acc, 0.70 F1; sMCI vs. pMCI (AIBL External): 75.36% Acc, 0.77 F1	33 Selected Features: (1) MRI Volumetric: 103 initial FreeSurfer volumes parcellated via DKT atlas, normalized by eTIV. Top: Left Hippocampus, Left Entorhinal, Right Amygdala; (2) Cognitive: MMSE, Logical Memory (Immediate and Delayed); (3) Demographic: Age, gender; (4) Genetic: ApoEε4 allele count.
Kim *et al.* [464]	2021	AD (228) vs. MCI (312) vs. CN (266)	ADNI	MRI (longitudinal)	Temporal GNN	Train/Test/Val (60/20/20)	CN/MCI/AD Prognosis (24-month): 53.5 ± 4.5% Accuracy	Graph Nodes (68 ROIs): Based on Desikan-Killiany atlas. Attributes per node (4): (1) Volume; (2) Area; (3) Average vertex-based thickness; (4) Std. Dev of thickness. Covariates: Age, gender, education, and MRI field strength. Top Nodes: Right Superior Frontal, Right Precentral, Left Superior Frontal, and Right Insula.

Continued on next page

Table S2 – *continued from previous page*

Reference	Year	Comparison Groups	Database	Data Type	Best Classifier	Validation	Best Result	Features Used
Gauriau *et al.* [415]	2021	Likely Normal vs. Likely Abnormal (Dataset A: 2839; B: 6442; C: 1489 subjects)	Heterogeneous Institutional Datasets	MRI (FLAIR)	CNN (Model A+B)	Binary classification (70/10/20 split for A, 90/3/7 for B)	Normal vs. Abnormal (Dataset C): 0.80 AUC, 0.78 F1, 77% Sen, 65% Spe (Model A+B)	MRI (Volumetric): 3D axial FLAIR sequence volumes downsampled to 256x256x16 voxels. Features include raw pixel data patterns representing infarcts, hemorrhages, neoplasms, infections, and small vessel disease.
Kapoor *et al.* [457]	2021	SMC (3821) vs. LMCI (4644) vs. CN (1568) vs. AD (2319) vs. EMCI (389)	ADNI-TADPOLE	Longitudinal EHR (MRI, genetic, cognitive)	Random Forest	5-stage stage classification (80/20 train/test split)	5-Stage: 99.9% Accuracy; AD per class: 99.88% Acc, 1.0 Prec, 0.98 sens.; The rest of stages per class: 100% Acc., 1.0 Prec., 1.0 sens.	36 Selected Features: (1) Demographic: Age, Gender, Education, Marital Status; (2) Genetic: APOE4; (3) Clinical/MRI: WholeBrain, Ventricles, Fusiform, Midtemp, Entorhinal, ICV, and Hippocampus volumes; (4) Cognitive: MMSE_bl, CDRSB_bl, ADAS11_bl, ADAS13_bl, MOCA, and RAVLT.

Continued on next page

Table S2 – *continued from previous page*

Reference	Year	Comparison Groups	Database	Data Type	Best Classifier	Validation	Best Result	Features Used
Pohl *et al.* [525]	2022	AD (507) vs. HC (462)	ADNI (TADPOLE)	MRI (3D MP-RAGE)	3D-CNN	Binary classification (Subject-level: 833 train, 67 val, 69 test)	AD vs. HC: 92.11% Acc (at 0.42 threshold), 0.88 F1, 96% Recall (HC), 86% Recall (AD)	MRI (3D Volumetric): 3D voxels (182x225x163 input) representing volumetric patterns. Top 25 ROI Features: Identified via LRP density, including Hippocampus, Amygdala, Entorhinal area, Temporal gyri (Middle, Inferior, Transverse), 3rd and 4th Ventricles, and Para-hippocampal gyrus.
Jain *et al.* [445]	2021	No Dementia (2560) vs. MCI (1790) vs. Moderate (717) vs. Serious Dementia (52)	Kaggle	MRI	VGG-19	4-class multi-label classification (stratified split: 8,000 train, 2,000 test)	4-Class: 86.42% Acc. (AUC 0.95); MCI Prediction: 77% Acc. (Best: Progressive resizing with GAN-augmented)	MRI (Pixel-based): 128x128 axial pixel intensity values; feature maps extracted via strided-convolutions and pooling. Features visualized as Grad-CAM heatmaps highlighting Learned spatial conformities of "tumor" or affected areas.

Continued on next page

Table S2 – *continued from previous page*

Reference	Year	Comparison Groups	Database	Data Type	Best Classifier	Validation	Best Result	Features Used
Shad *et al.* [547]	2021	Non-demented vs. Very Mild vs. Mild vs. Moderate Demented (6400 scans total)	Kaggle (hybrid dataset)	MRI	VGG-16	4-class multi-class classification (80/20 train/test split)	4-Class: 86.82% Acc., 75.58% Prec., AUC 0.922	MRI (Physics/Pixel-based): T1 relaxation time patterns (longitudinal relaxation of NMV). Pixel features extracted through 16 weighted layers; feature reuse via dense connectivity. Visual evidence identified as key pixels/regions via LIME-based explainer.

Continued on next page

Table S2 – *continued from previous page*

Reference	Year	Comparison Groups	Database	Data Type	Best Classifier	Validation	Best Result	Features Used
Mehmood *et al.* [501]	2021	AD vs. NC (75 AD, 85 NC); NC vs. EMCI (85 NC, 70 EMCI); NC vs. LMCI (85 NC, 70 LMCI); EMCI vs. LMCI (70 EMCI, 70 LMCI)	ADNI	MRI	Redesigned Deep CNN with transfer learning	80% Training (80% train / 20% validation) / 20% Testing split	AD vs. NC: 98.73% Acc, 98.19% Sen, 99.09% Spec (Group B, Aug); NC vs. EMCI: 87.06% Acc, 86.61% Sen, 86.63% Spec (Group B, Aug); NC vs. LMCI: 89.15% Acc, 89.24% Sen, 88.86% Spec (Group B, Aug); EMCI vs. LMCI: 83.72% Acc, 82.09% Sen, 85.13% Spec (Group B, No Aug); EMCI vs. AD: 84.15% Acc, 83.76% Sen, 83.12% Spec (Group B, Aug); LMCI vs. AD: 82.31% Acc, 82.18% Sen, 82.03% Spec (Group B, No Aug). Group B generally achieved higher performance, suggesting that freezing more layers in this specific transfer learning task was more effective	Automated Features: Hierarchical low-to-high level features automatically extracted from Gray Matter (GM) scans via deep convolutional layers. The model focuses on discriminative features in GM density that correlate with cognitive performance. Group A: The first three convolutional blocks (blocks 1, 2, and 3) of the VGG network were frozen. The first four convolutional blocks (blocks 1, 2, 3, and 4) were frozen.

Continued on next page

Table S2 – *continued from previous page*

Reference	Year	Comparison Groups	Database	Data Type	Best Classifier	Validation	Best Result	Features Used
Mofrad *et al.* [506]	2021	HC vs. converted to MCI (133 HC, 133 cMCI); stable MCI vs. converted to AD (279 sMCI, 279 cAD)	ADNI and AIBL	MRI (longitudinal)	Ensemble (Soft voting strategy)	15-fold CV (training); independent test set from AIBL	Task 1 (HC vs. rHC): 73% Acc, 83% sens, 69% prec (Best: Combination lin vent, nonlin hipp). Task 2 (sMCI vs. rMCI): 78% Acc, 85% Pre, 79% Rec, 76% F1 (Best: Combination, non-linear LME).	Longitudinal ROI Trajectories: Volumetric measures of bilateral 3D ROIs, specifically the Hippocampi and Lateral Ventricles (used as a proxy for total brain atrophy). Derived Parameters: (1) Intercept and slope of random effects from Linear Mixed Effects (LME) models; (2) Deviation of volume at first and last scans; (3) Atrophy slope (volume difference divided by years between scans). Also includes subjects' Sex, average Age, and age at last scan.
Das *et al.* [391]	2021	Demented vs. Healthy (Total 1437 subjects); CDR groups: Mild (500), Moderate (274), Severe (54), Healthy (293) for train-test	ADNI	MRI	SVM	100-fold CV (80% train/ 20% test); separate validation set	CDR Mild vs. Healthy: 86.26% Acc, 0.92 AUC; CDR Moderate vs. Healthy: 89.53% Acc, 0.95 AUC; CDR Severe vs. Healthy: 96.86% Acc, 0.98 AUC; ADNI MCI vs. HC: 84.87% Acc, 0.93 AUC; ADNI AD vs. HC: 91.86% Acc, 0.97 AUC.	Corpus Callosum (CC) Morphometrics (28 total): (1) Area-based: CC area, convex hull area, bending coefficient, and solidity; (2) Length-based: Perimeter, major/minor axis length, and horizontal/vertical spans; (3) Geometric: Eccentricity, orientation, and tangent mean/SD; (4) Intensity: Mean, total, and threshold intensities.

Continued on next page

Table S2 – *continued from previous page*

Reference	Year	Comparison Groups	Database	Data Type	Best Classifier	Validation	Best Result	Features Used
Battineni *et al.* [372]	2021	Demented vs. Non-demented (78 D, 72 ND)	OASIS longitudinal MRI	MRI (longitudinal)	Gradient Boosting	80:20 Train/ Test split; 10-fold CV	D vs. ND: 97.58% Acc, 0.98 Pre, 0.96 Rec, 0.97 F1, 0.981 AUROC (Gradient Boosting).	14 Demographic & MRI Features: Gender, EDUC (education years), SES (socioeconomic status), MMSE, CDR, e-TIV (estimated total intracranial volume), n-WBV (normalized whole brain volume), ASF (atlas scaling factor), Age, and MR delay.
Li and Yang [482]	2021	AD vs. CN (260 AD, 300 CN)	ADNI	MRI	3D-ResNet (with Transfer Learning)	500 images training / 60 images testing; stratified 5-fold CV	AD vs. CN: 95% Acc, 94.3% Sen, 96% Spec, 0.995 AUC (3D-ResNet with Transfer Learning).	Voxel-wise Features: Intensity values from individual voxels. Features are selected using ANOVA F-score (top 10% relevant voxels). Discriminative Regions: The model identifies the Cerebellum, Cerebral Cortex, and White Matter as the most significant areas contributing to classification.

Continued on next page

Table S2 – *continued from previous page*

Reference	Year	Comparison Groups	Database	Data Type	Best Classifier	Validation	Best Result	Features Used
Hazarika *et al.* [429]	2021	AD vs. MCI vs. CN (70 AD, 70 MCI, 70 CN)	ADNI	MRI (MP-RAGE)	Improved DenseNet-121, MobileNet-V1	Train/Val/Test split	CN vs. MCI (60–69y): 93% Acc, 93% Prec, 94% F1 (MobileNet-V1). MCI vs. AD (60–69y): 92% Acc, 90% Prec, 93% F1 (Improved DenseNet-121). CN vs. AD (60–69y): 92% Acc, 94% Prec, 93% F1 (Improved DenseNet-121). 3-class (60–69y): 90% Acc. (Improved DenseNet-121)	T1 MRI Slices: Deep features (edges, corners) from Informative slices selected by high entropy values. Targeted analysis of Hippocampus changes and Grey Matter (GM) atrophy across age groups (60–90 years).
Khan *et al.* [460]	2022	AD vs. MCI vs. CN (193 AD, 396 MCI, 229 CN)	ADNI-1	Cognitive and Demographic data	Hybrid Cognitive Model (HCM-2)	Train/Test (80/20), 10 iterations of 5-fold repeated stratified CV	3-class (CN/MCI/AD): 95.12% Acc, 95.12% Sens, 97.50% Spec, 0.990 AUC. (Validation: 10-fold CV; 80/20 train-test split).	Cognitive & Demographics: Reduced set of 5 dominant markers: MMSE, CDRSB, RAVLT-I, DIGITSCOR, and LDETOTAL. Demographics: Age, gender, years of education, and marital status.
Sharma *et al.* [550]	2022	4-class: Non-Demented, Very Mild, Mild, and Moderate dementia	ADNI; Kaggle	MRI (structural)	Neural Network with VGG16	Train/Test (80/20)	4-class (Dataset 1): 90.4% Acc, 0.905 Prec, 0.904 Recall, 0.969 AUC. 3-class (Dataset 2): 71.1% Acc, 0.850 AUC.	2D T1 MRI Images: 224x224 pixel scans. Automated deep features extracted via 16-layer VGG16 architecture (convolutional, pooling, and fully connected layer activations).

Continued on next page

Table S2 – *continued from previous page*

Reference	Year	Comparison Groups	Database	Data Type	Best Classifier	Validation	Best Result	Features Used
Nguyen *et al.* [512]	2022	AD vs. CN (231 AD, 231 CN)	ADNI	MRI, demographics, cognitive scores	ENS-ALL (Stacked ensemble)	5-fold cross-validation	AD vs. CN: 0.962 Mean AUC. (Validation: 5-fold CV; per-subject split).	Multi-modal Voxel-wise Features: 3D T1 MRI volumes (182x218x182). ROI crops of Hippocampus (40x40x40) and Hippocampus-extended (90x90x90). Combined with Age, Gender, MMSE, and CDR scores.
Liu *et al.* [491]	2022	AD vs. MCI vs. CN (748 AD, 1089 MCI, 782 CN)	ADNI; NACC	MRI	3D Deep CNN	Train/Val/Test, for additional external validation	AD vs. rest: 0.8921 AUC. CN vs. rest: 0.8759 AUC. MCI vs. rest: 0.6259 AUC. (Data: ADNI held-out test set).	T1 MRI Volumes: DL model extracted voxel features focusing on the 4th ventricle, parahippocampal gyrus, and lateral ventricles. ROI model used 138 features including volumes and cortical thickness of the left/right hippocampus and entorhinal cortex.
Elgammal *et al.* [402]	2022	AD vs. MCI vs. CN (250 AD, 250 MCI, 250 CN)	Kaggle; ADNI	MRI (1.5T)	Multifractal analysis with KNN	3-class (ADNI) Train/ Test (71/29); (Kaggle) Train/Test (80/20)	Normal vs. Pathological: 100% Acc. V.Mild vs. Mild: 100% Acc. V.Mild vs. Moderate: 100% Acc. ADNI 3-class: 99.3% Acc. Kaggle 4-class: 99.4% Acc.	10 Multifractal Features: Information dimension (D1), correlation dimension (D2), local dimension at max singularity (alpha0), min/max local dimensions (alpha_min/max), spectrum end values (f(alpha_min/max)), spectrum width (W), symmetrical shift, and apparent brain area.

Continued on next page

Table S2 – *continued from previous page*

Reference	Year	Comparison Groups	Database	Data Type	Best Classifier	Validation	Best Result	Features Used
Sharma *et al.* [551]	2022	4-class: Non Demented (3200), V.M.D (1966), M.D (896), Mod. D (64)	Kaggle	MRI (grayscale)	Hybrid DenseNet201-Gaussian NB	Train/Test (80/20)	4-class (ND/VMD/MD/ModD): 91.75% Acc, 96.5% Spec, 90.25 F1-score. (Validation: 80/20 train-test split).	2D MRI Grayscale Pixels: (208x176x3) pixel image inputs. Automated feature maps from DenseNet-121 (1024 filters) and DenseNet-201 (1920 filters) extracted through transfer learning across five convolutional blocks.
Yu *et al.* [600]	2022	AD vs. NC (422 AD, 826 NC)	ADNI	MRI	MAXNet	Binary (AD vs. NC); Validation: Split (1779 train / 427 validation / 575 test)	AD vs. NC: 95.4% Accuracy; 0.980 AUC (Subset 3)	Multi-scale Latent Representations: Automatically learned class-discriminative features from intermediate and last layers. Anatomical markers: Identified atrophy in the cerebral cortex and hippocampus, enlargement of lateral ventricles (frontal/temporal horns), and enlarged sulcal spaces.

Continued on next page

Table S2 – *continued from previous page*

Reference	Year	Comparison Groups	Database	Data Type	Best Classifier	Validation	Best Result	Features Used
Lombardi *et al.* [493]	2022	AD vs. MCI vs. NC (965 AD, 2912 MCI, 2408 NC samples)	ADNI	Neuropsychological test scores	Random Forest	Multi-class (NC, MCI, AD); Validation: Leave-one-subject-out CV; 100 repetitions with random undersampling	One-vs-Rest (Avg Metrics): AD (0.97 AUC, 72% Sens, 97% Spec); NC (0.88 AUC, 73% Sens, 84% Spec); MCI (0.82 AUC, 75% Sens, 75% Spec). Global: 75% Acc., AUC 0.89	10 Cognitive Markers: ADAS11, ADAS13, MMSE, MoCA, FAQ, RAVLT (immediate, learning, % forgetting), ECogPt total, and ECogSP total. Feature Sensitivity: FAQ and EcogSPTot are primary markers for NC; ADAS13 is critical for detecting the AD neurodegeneration spectrum.
Xu *et al.* [596]	2022	CN vs. MCI vs. AD (564 CN, 363 MCI, 147 AD)	ADNI 3	Cognitive scores (CS)	RF	Nested CV with 10 repetitions	Multi-class Overall: 0.878 F-measure (Accuracy). Per-class F-measure: CN (0.937); MCI (0.820); AD (0.797).	8 Initial Cognitive Features: CDRSB, MMSE, EcogSPTotal, RAVLT_perc_forgetting, LDELTOTAL, FAQ, ADAS11, and MOCA. Best Subset (CMER): Clinical dementia rating sum of boxes (CDRSB), MMSE, Everyday cognition total (EcogSPTotal), and RAVLT percent forgetting.

Continued on next page

Table S2 – *continued from previous page*

Reference	Year	Comparison Groups	Database	Data Type	Best Classifier	Validation	Best Result	Features Used
Zhang *et al.* [607]	2022	AD vs. NC (200 AD, 231 NC); pMCI vs. sMCI (172 pMCI, 232 sMCI)	ADNI-1, -2, -3	MRI	3D-ResAttNet34	Binary (AD vs. NC and pMCI vs. sMCI); Validation: 5-fold CV on ADNI-1; Independent test on ADNI-2/3	AD vs. NC: 91.3% Accuracy; 0.984 AUC; 91.0% Sens; 91.9% Spec. pMCI vs. sMCI: 82.1% Accuracy; 0.920 AUC; 81.2% Sens; 80.9% Spec.	Whole-brain 3D Voxel Features: Automatically extracted high-dimensional non-linear features from sMRI scans. Decision-driving ROIs: Explainable Grad-CAM highlights significant variations in the hippocampus, lateral ventricle, and various cortical regions.
Ruengchai-jatuporn *et al.* [538]	2022	MCI vs. Healthy aging control (267 MCI, 651 Healthy)	King Chulalongkorn Memorial Hospital (Thailand)	Digital drawing tasks (CDT, Cube, Trail)	Multi-input Conv-Att with soft label	Binary (MCI vs. healthy); Validation: Stratified random split (70% train / 15% val / 15% test)	MCI vs. Healthy: 0.8116 Accuracy; 0.8375 AUC; 0.6539 F1-score. Baseline (CDT only): 0.7478 Accuracy; 0.7429 AUC.	Spatio-temporal Drawing Features: (1) Clock Drawing (CDT): hands indicating 12:55 instead of 11:10. (2) Cube-copying: unusual paths and Necker cube structure. (3) Trail-making (TMT): paths that should not be drawn (e.g., segments B-4 or C-D).

Continued on next page

Table S2 – *continued from previous page*

Reference	Year	Comparison Groups	Database	Data Type	Best Classifier	Validation	Best Result	Features Used
EL-Geneedy *et al.* [399]	2022	NC vs. VmD vs. MD vs. MoD (3200 images ND, 2240 images VMD, 896 images MD, 64 images MoD)	OASIS-3	MRI	Proposed SCNN	80% Train / 20% Test split; 16% of training as validation	Geral (Multiclass): test Acc. 99,68%, Sens. 100%, Spec. 100%, AUC 100%.	Learned Deep Features: Automatically learned higher-level meaningful local and global features extracted directly from 2D brain representations. Focuses on highlighting structural similarity while maximizing inter-class variance.
Pui Ching *et al.* [592]	2024	AD vs. MCI vs. NC (924 AD, 1260 MCI, 1240 NC images)	ADNI	MRI (axial-plane)	Transfer Learning-based EfficientNet-B0	80% Train / 20% Validation split; unseen images for testing	Multiclass (AD vs. MCI vs. NC): Train Acc. 98,93%, Validation Acc. 87,17%	Axial-plane Image Features: Comprehensive feature representations extracted via 2D depth-wise convolutional units and inverted mobile bottleneck convolutions (MBConv). Focuses on anatomical tissue shrinkage in the hippocampus.
Raza *et al.* [533]	2023	AD vs. NC vs. LMCI vs. MCI (1254 AD, 1254 NC, 1254 LMCI, 1254 MCI images)	ADNI	MRI	DenseNet-169 with Transfer Learning	80% Train / 20% Test split (with separate validation set)	AD vs. NC/MCI/LMCI: Acc. 99,68%, Spec. 99,69%, Sens. 99,65%. MCI vs. Others: Acc. 99,52%. LMCI vs. Others: Acc. 98,16%. NC vs. Others: Acc. 98,33%. Multiclass: Acc. 97,84%.	2D Gray Matter (GM) Features: Deep features learned from axial, coronal, and sagittal planes focusing on cortical thickness, GM density, ventricle expansion, and regional shrivel. Includes macro-features extracted via 7x7 kernels and comprehensive knowledge maps from Dense Blocks.

Continued on next page

Table S2 – *continued from previous page*

Reference	Year	Comparison Groups	Database	Data Type	Best Classifier	Validation	Best Result	Features Used
Gill *et al.* [417]	2024	AD vs. CN vs. pMCI vs. sMCI (45 AD, 89 CN, 110 pMCI, 100 sMCI)	ADNI	MRI (NIFTI format)	Random Forest	80/20 Train/ Test split	Multiclass: Acc: 66.1%	Texture analysis: First-Order Statistical (FOS) features (variance, skewness, kurtosis) and Gray-Level Co-occurrence Matrix (GLCM) features (contrast, correlation, homogeneity, dissimilarity, energy, ASM, entropy) calculated with a distance of 5 and angles of 0°, 45°, 90°, and 135°.
Saleh *et al.* [541]	2023	ND (Non-Demented) vs. MID (Mild) vs. VMD (Very Mild) (2560 ND, 717 MID, 1792 VMD in training)	Kaggle (Alzheimer's 4-class)	MRI (2D cross-sections, grayscale)	DenseNet201 with Data Augmentation	Train/Val/ Test split (80% train, 20% test; 20% of train for val)	Multiclass: 96.05% Acc., 0.99 AUC	Deep visual features: Low-level features (edges, textures, forms) and high-level representations (object patterns, structures) automatically extracted by pre-trained DenseNet convolutional layers.
Ramalho *et al.* [531]	2024	NC vs. MCI (250 NC, 68 MCI)	ADNI 1	MRI (axial, coronal, sagittal)	Custom CNN (Coronal slice)	70/15/15 Train/Val/ Test split	NC vs. MCI: Acc: 99%, F1: >91%, Prec: >91%, Rec: >91%	Morphological patterns: High-level hierarchical features learned via convolutional and pooling layers; Coronal slices provide specific details on frontal atrophy and hippocampal changes.

Continued on next page

Table S2 – *continued from previous page*

Reference	Year	Comparison Groups	Database	Data Type	Best Classifier	Validation	Best Result	Features Used
Silva *et al.* [555]	2023	CN vs. MCI vs. AD (24 CN, 44 MCI, 21 AD)	ADNI	MRI	Bagged Trees (AD vs. CN); Fine KNN (CN vs. MCI); QSVM (AD vs. MCI)	Leave-one-out cross-validation	AD vs. CN: Acc. 93,3% (Bagged Trees). CN vs. MCI: Acc. 88,2% (FGSVM). AD vs. MCI: Acc. 87,7% (QSVM). Multiclass (All vs. All): Acc. 75,3% (Subspace KNN).	2187 Textural Features (GLCM-based): Extracted from DWT subbands (LL, HL, LH, HH) including contrast, correlation, energy, homogeneity, entropy, variance (line/column), and standard deviation (line/ column). Features are extracted across axial, coronal, and sagittal planes.
Tajammal *et al.* [564]	2023	AD vs. CN vs. EMCI vs. MCI vs. LMCI vs. SMC (24 AD, 24 CN, 24 EMCI, 24 MCI, 22 LMCI, 24 SMC)	ADNI	fMRI	Max-voting Ensembling	Training and testing phases (subject-independent sampling)	AD vs. CN: 99.6% Acc, MCI vs. AD: 99.4% Acc, CN vs. MCI: 99.8% Acc, Multi-class: 98.8% Acc	Spatio-temporal signals: Functional connectivity features derived from blood oxygenation level signals (rs-fMRI) across 3D brain volumes; captured via DL models (AlexNet, VGG-16, ResNet-18).
Alatrany *et al.* [352]	2024	NC vs. MCI vs. AD (NACC: 18,171 NC, 10,043 MCI, 15,687 AD)	NACC (External: ADNI)	Clinical/ Neuropsychological Records	SVM	5-fold CV (Training) + separate hold-out Test (80/ 20)	External ADNI-NC vs. AD: 99% Acc, 98.9% F1. NC vs. MCI: 99.6% Acc. MCI vs. AD: 88.9% Acc. Multi: 90.5% Acc, 90.7% F1	Clinical indicators: CDR (Memory, Judgment, Orientation, Communication), FAQ (Bills, Taxes, Travel, Shopping, Meal Prep, etc.), NPI-Q (behavioral domains like agitation/anxiety), and Geriatric Depression Scale (GDS).

Continued on next page

Table S2 – *continued from previous page*

Reference	Year	Comparison Groups	Database	Data Type	Best Classifier	Validation	Best Result	Features Used
El-Assy *et al.* [400]	2024	AD vs. CN vs. MCI vs. EMCI vs. LMCI (171 AD, 580 CN, 233 MCI, 240 EMCI, 72 LMCI)	ADNI	MRI	Proposed Concatenated CNN	80/20 split (Training vs. Test); separate Validation set	3-way (AD/CN/MCI): 99.43% Acc. 4-way: 99.57% Acc. 5-way: 99.30% Acc, 99.13% MCC	Hierarchical structural features: Learned via multi-scale filters (3x3 kernels for CNN1; 5x5 kernels for CNN2); Grad-CAM visual heatmaps identify specific discriminating brain regions for different classes.
Sorour *et al.* [559]	2024	Demented vs. Non-Demented (3200 Demented, 3200 Non-Demented)	Kaggle (ADNI)	MRI	CNNs-LSTM-with-Aug	80/20 Train-Test split (5120 train, 1280 test)	Demented vs. Non-Demented: 99.92% Accuracy, 100% Precision, 99.50% Recall, 100% Specificity, and 99.70% F1-score.	Spatial & Temporal Deep Features: Convolutional filters extract local spatial hierarchies (edges/ textures); LSTM layers extract temporal dependencies in sequential data. pre-processing features include pixel-level normalization (scaled 0 to 1) and color modification for consistency.

Continued on next page

Table S2 – *continued from previous page*

Reference	Year	Comparison Groups	Database	Data Type	Best Classifier	Validation	Best Result	Features Used
Slimi *et al.* [558]	2024	4 Classes: Non-Demented, Very Mild, Mild, Moderate Demented (>5000 images total)	Kaggle (sourced from ADNI)	MRI	Hybrid DenseNet121 & Xception	80/20 Train-Test split; 5-fold cross-validation	4-Class (Multi-class): 99.85% Accuracy, 99.85% Precision, 99.88% Recall, 99.85% F1-score, 99.9% AUC.	Transfer Learning Fusion Features: Concatenated feature maps from DenseNet121 (dense connectivity for feature reuse and gradient flow) and Xception (depthwise separable convolutions for efficient parameter reduction). Output features are harmonized via a convolutional layer (3 filters, kernel 3x3) and concatenated into a fused tensor.
Ganesan *et al.* [413]	2024	ADNI: (335 AD, 542 MCI, 785 NC). AIBL: (79 AD, 110 MCI, 307 NC)	ADNI and AIBL	MRI	DBN (Deep Belief Network)	Unsupervised pre-training (RBM layers) followed by supervised fine-tuning (Linear regression).	Multi-class (AIBL): 99.92% Accuracy, 99.82% Spec, 99.78% Sens. Multi-class (ADNI): 99.80% Accuracy, 99.88% Spec, 99.72% Sens. NC vs. MCI: 99.88% Accuracy. MCI vs. AD: 99.81% Accuracy. AD vs. NC: 99.71% Accuracy.	Hand-crafted Texture Vectors (6,542 for ADNI): Fusion of 1) Local Binary Pattern (LBP): Spatial local patterns/ grey-scale contrast using Ra=1, P=8; 2) Local Directional Pattern variance (LDPv): Encodes directional patterns and contrast information via directional response mean variance.

Continued on next page

Table S2 – *continued from previous page*

Reference	Year	Comparison Groups	Database	Data Type	Best Classifier	Validation	Best Result	Features Used
Pasnoori *et al.* [523]	2024	4 Classes: Non-Demented (3200), Very Mild (2240), Mild (897), Moderate (64)	Kaggle	MRI	SVM, Ensemble, and Neural Network (Perfect scores)	10-fold cross-validation (60% train, 20% validation, 20% test)	4-Class (Multi-class): 100% Accuracy, 100% Recall, 100% Specificity, 100% Precision. Binary (Merged Stages): 100% Accuracy.	17 Hand-crafted Histogram Features: Grey and white matter volume, GWR (Grey-to-White matter Ratio), Shrinkage (atrophy indicator), statistical moments (Mean/SD), thresholds (Th1, Th2), slopes ($\alpha1$, $\alpha2$), and 3 Euclidean distance parameters (d1, d2, d3) between histogram maxima/minima.
Nair Bini Balakrishnan *et al.* [368]	2025	AD vs. CN (590 AD, 229 CN); Mild vs. Moderate vs. Non vs. Very Mild Demented (1600 per class, 6400 total)	ADNI, Kaggle (AD Database)	MRI (structural)	Proposed DRL-MFORNN	80/20 and 70/30 train/test splits	AD vs. CN (ADNI 80/20): 98.4% Acc., 98.1% Prec., 98% Recall, 0.92 AUC; AD vs. Normal (Kaggle 80/20): 97.7% Acc., 97.3% Prec., 97.3% Recall, 0.98 AUC.	Optimized subsets: Most informative and highly correlative features selected from preprocessed MRI pixels via Moth Flame Optimization. Temporal & Hierarchical: Captures temporal dependencies and hierarchical representations within each image sample to differentiate normal vs. AD patterns.

Continued on next page

Table S2 – *continued from previous page*

Reference	Year	Comparison Groups	Database	Data Type	Best Classifier	Validation	Best Result	Features Used
Shakhnoza Muksimova *et al.* [510]	2025	NC vs. MCI vs. AD (275 NC, 413 MCI, 280 AD)	ADNI	MRI (structural – white matter, grey matter)	Proposed CNN with Attention & MSB	70/15/15 train/test split/validation	Multiclass: 98.40% Acc., 98.10% Sens., 99.21% Spec.; Normal: 99.35% Acc.; MCI: 97.82% Acc.; AD: 98.79% Acc.	Multi-scale hierarchies: Captures fundamental "low-level" features (edges, textures) in initial layers and "high-level" abstract features (objects, relationships, shapes) in deeper layers. Attention-weighted features: Global connection-wise attention scores for spatial and channel-level dependencies. Temporal Dynamics: Abstract high-level temporal features captured by LSTM networks instead of traditional FC layers.

Continued on next page

Table S2 – *continued from previous page*

Reference	Year	Comparison Groups	Database	Data Type	Best Classifier	Validation	Best Result	Features Used
Mohamed Amine Mahjoubi *et al.* [499]	2025	Mild vs. Moderate vs. Non vs. Very Mild Demented (3200 per class, 12,800 total)	ADNI	MRI	ResNet50 with Adam Optimizer	75/15/10 train/validation/test split	Adam: 97.66% Accuracy, 7.10% test loss; Multiclass AUCs: 1.00 (Moderate), 0.99 (Non), 0.98 (Mild), 0.97 (Very Mild).	Transfer Learning Features: Automated hierarchical feature extraction leveraging weights from ImageNet-pre-trained ResNet-50. Residual Mapping: Learns deep residual features via "skip connections" to preserve identity mapping and feature integrity across 50 layers. Categorical: Features optimized to minimize categorical cross-entropy loss across four dementia severity classes.

Continued on next page

Table S2 – *continued from previous page*

Reference	Year	Comparison Groups	Database	Data Type	Best Classifier	Validation	Best Result	Features Used
Muhammad Zahid Hussain *et al.* [440]	2025	Mild vs. Moderate vs. Non vs. Very Mild Demented (After augmentation: 2565 Mild, 2570 Moderate, 2520 Non, 2599 Very Mild)	Kaggle (Alzheimer dataset), OASIS	MRI	AlexNet with ADAM solver	Training and testing (80/20)	Kaggle Multiclass: 99.4% Accuracy, 99.4% precision/recall/F1; OASIS Multiclass: 98.2% Accuracy, 98.2% precision/recall/F1.	Convolutional Local Features: Automated extraction of low-level features such as brain edges and textures from MRI scans. Deep Pre-trained Features: 62.3 million parameters (AlexNet) or multi-layer hierarchies (GoogLeNet/ MobileNetV2) learned from natural images and fine-tuned for neuroimaging. Salient Regions: Focus on regions visualized by Grad-CAM, typically aligning with clinical AD criteria.
Yingtong Gan *et al.* [412]	2025	AD vs. CN (440 AD, 287 CN – ADNI Test Set)	ADNI, OASIS, MIRIAD	MRI (1.5T and 3T)	Proposed DANet-B2	4:1 train/ validation split (per-patient basis)	AD vs. CN (ADNI): 0.933 AUC, 94.5% Accuracy; AD vs. CN (OASIS): 0.865 AUC, 81.4% Accuracy; AD vs. CN (MIRIAD): 0.820 AUC, 77.6% Accuracy.	Dense Feature Reuse: Multi-scale features from every preceding layer concatenated to prevent loss of intermediate details. Volumetric Context: 3D convolutional patch embeddings (7x7x7) for depth information. Pathological Focus: Linear attention-weighted features focusing on hippocampal shrinkage, cortical sulci widening, and gradual ventricular enlargement.

Continued on next page

Table S2 – *continued from previous page*

Reference	Year	Comparison Groups	Database	Data Type	Best Classifier	Validation	Best Result	Features Used
F. Binzagr and A. Abulfaraj [376]	2025	AD vs. CN vs. EMCI vs. LMCI vs. MCI (500 AD, 500 CN, 480 EMCI, 432 LMCI, 500 MCI)	ADNI, OASIS	MRI	Proposed In-GSA with ELM Classifier	Benchmark evaluation on two datasets (ADNI and OASIS)	ADNI (5-class): 96.67% Acc., 97.76% avg. Prec., 96.63% avg. Recall, 97.90% avg. AUC; OASIS (4-class): 97.99% Acc., 98.00% avg. Prec., 98.66% avg. AUC.	Abstract Global Features: High-level activation maps (Z) extracted from InceptionV3. Generalized Attention: Concurrent global spatial dependencies (relationships between spatial locations) and channel-wise dependencies (importance of specific channels). Category-Specific: Nuanced, fine-detailed AD information refined through 4 GSA heads to suppress noise.
Clark *et al.* [388]	2016	MCI-con vs. MCI-non vs. CN (24 converters, 83 non-converters, 51 CN)	ImaGene study (UCLA ADRC)	MRI, verbal fluency word lists	Ensemble with "novel" fluency scores	Leave-one-out cross-validation	Novel Fluency Scores: AUC 0.872 (84.1% Accuracy, 70.8% sens., 88.0% spec.)	Novel Fluency Scores: Semantic/orthographic/phonologic coherence, lexical frequency (Google n-grams), algebraic connectivity, radius, transitivity, and syllable counts. MRI Measures: Hippocampal volume, total ventricular volume, and 30 ICA components derived from bihemispheric cortical thickness maps.

Continued on next page

Table S2 – *continued from previous page*

Reference	Year	Comparison Groups	Database	Data Type	Best Classifier	Validation	Best Result	Features Used
Jha *et al.* [448]	2017	AD vs. HC (28 AD, 98 HC)	OASIS	MRI	FNN with DTCWT and PCA	10-fold repeated 50 times	AD vs. HC: Acc 90.06% (Sens 92.00%, Spec 87.78%, Precision 89.6%)	Total features: 14 Principal Components (PCs) preserving 90.44% variance. Raw Source Features: coefficients extracted using 5-level Dual-tree Complex Wavelet Transform (DTCWT) from 32 axial slices (totaling 24,576 raw features per subject). Demographics: Gender, age, education, socioeconomic status (SES).
Lama *et al.* [473]	2017	AD vs. MCI vs. HC (70 AD, 74 MCI, 70 HC)	ADNI	MRI	RELM with PCA feature selection	10-fold split into 90/10 train/test	NC vs. AD: Acc 80.32% (Sens 83.37%, Spec 78.82% via SVM); NC vs. MCI vs. AD: Acc 61.58% (Sens 54.00%, Spec 62.25% via RELM)	Selected Features: 10 Principal Components (PCs) derived from volumetric data. Biomarkers (fM5): Grey matter tissue volumes extracted from block brain regions automatically parcellated by FreeSurfer. Potential indices: Cortical thickness, surface area, folding indices, and mean curvature.

Continued on next page

Table S2 – *continued from previous page*

Reference	Year	Comparison Groups	Database	Data Type	Best Classifier	Validation	Best Result	Features Used
Hojjati *et al.* [433]	2017	MCI-C vs. MCI-NC (18 MCI-C, 62 MCI-NC)	ADNI	rs-fMRI	SVM with linear kernel	9-fold cross-validation	MCI-C vs. MCI-NC: 91.4% Acc., 83.24% Sen., 90.1% Spe., 0.95 AUC.	913 Graph Features: 10 local measures (e.g., betweenness centrality, clustering coefficient) across 90 AAL regions + 13 global measures (e.g., transitivity, small-worldness). Best subset (10 features) includes measures in Angular L, ParaHippocampal R, and Rolandic Oper L.
Beheshti *et al.* [374]	2017	AD vs. HC vs. sMCI vs. pMCI (83 AD, 61 HC, 42 sMCI, 45 pMCI)	J-ADNI	MRI	Proposed Histogram-based SVM	300 random runs (90% train, 10% test)	AD vs. HC: 84.17% Acc. MCI vs. HC: 70.38% Acc. pMCI vs. sMCI: 61.05% Acc. AD vs. sMCI: 67.59% Acc. AD vs. pMCI: 62.84% Acc..	Histogram Feature Vectors (HFVs): Statistical pattern representations (1024 bins) derived from patient-specific anatomical brain connectivity networks based on individual gray matter morphometry.

Continued on next page

Table S2 – *continued from previous page*

Reference	Year	Comparison Groups	Database	Data Type	Best Classifier	Validation	Best Result	Features Used
Li *et al.* [480]	2018	AD vs. NC (199 AD, 229 NC); MCI vs. NC (403 MCI, 229 NC)	ADNI	MRI	Multiple cluster DenseNets (Ensemble)	5-fold cross-validation	AD vs. NC: Acc 89.5%, AUC 92.4%, SEN 87.9%, SPE 90.8%; MCI vs. NC: Acc 73.8%, AUC 77.5%, SEN 86.6%, SPE 51.5%.	Local: 3D image patches (32×32×32) extracted at 2-voxel steps; Dimensionality: PCA reduced 32,768 voxels per patch to 2000. Relevant ROIs: Top regions identified via relevance mapping include Amygdala, Hippocampal, Entorhinal, Thalamus, Temporal pole, and Parahippocampal gyrus.
Ghazal *et al.* [416]	2018	AD vs. NC (70 AD, 70 NC); AD vs. MCI (70 AD, 70 MCI); MCI vs. NC (70 MCI, 70 NC)	CADDementia; ADNI	MRI	3D-DSA-CNN	Ten-fold cross-validation	AD vs. NC: Acc 99.3%, AUC 99.3%. AD vs. MCI: Acc 100%, AUC 100%; MCI vs. NC: Acc 94.2%, AUC 97.1%; AD+MCI vs. NC: Acc 95.7%, AUC 96.1%.	Hierarchical 3D feature maps: captures generic anatomical shape variations. Biomarkers: Specifically models cortex thickness and volume, brain size, ventricle size, and hippocampal shape. Task-specific: Upper layers extract fine-tuned class-discriminative features.

Continued on next page

Table S2 – *continued from previous page*

Reference	Year	Comparison Groups	Database	Data Type	Best Classifier	Validation	Best Result	Features Used
Hett *et al.* [432]	2018	AD vs. MCI vs. CN (186 AD, 388 MCI [223 sMCI, 165 pMCI], 226 CN)	ADNI	MRI	SVM using adaptive fusion of texture-based grading	Repeated stratified 10-fold CV (50 iterations)	CN vs. AD: 91.3% Acc, 94.6% AUC; CN vs. pMCI: 92.0% AUC; AD vs. sMCI: 82.6% AUC; sMCI vs. pMCI: 72.2% Acc, 76.1% AUC	Multi-directional texture grading maps focusing on structural alterations of the Hippocampus (HIPP). Features are bins from concatenated histograms of right and left HIPP grading maps (bins set via Sturge's formula). Texture was extracted using 5 optimal directions at full resolution. All values are age-corrected via linear regression based on the CN group.
Li *et al.* [481]	2019	AD vs. NC (12 AD, 14 NC in local Tongji dataset; 117 AD scans, 175 NC scans in source ADNI)	Tongji Hospital (local) and ADNI (shared source)	rs-fMRI	Linear Discriminant with modified Subspace Alignment (With Adaptation)	Leave-one-out cross-validation (LOOCV) for the target (Tongji) dataset	AD vs. NC (Tongji): 84.6% Acc., (92% Sen., 79% Spe., 80% AUC)	Functional Connectivity: 4005-dimensional feature vector of pairwise Pearson correlation coefficients between 90 ROIs (AAL atlas). Top discriminative connections involve regions like the Hippocampus_R to Frontal_Sup_L, Frontal_Mid_L, and Temporal_Inf_R to Heschl_R.

Continued on next page

Table S2 – *continued from previous page*

Reference	Year	Comparison Groups	Database	Data Type	Best Classifier	Validation	Best Result	Features Used
Ajagbe *et al.* [349]	2021	AD Stages: Mild, Moderate, Non-demented, Very Mild (Total 6400 images)	Kaggle	MRI	VGG-19	Train (4098), Test (1279), Validation (1023) images split	Multi-classification: 77.66% Acc, 81.55% AUC, 58.48% Pre, 36.67% Rec (VGG-19 best condition).	Automated Deep Features: Hierarchical feature representation mimicking the human visual cortex. Features are extracted through 13–16 convolutional layers, focusing on brain atrophy in the Cerebral Cortex, Ventricle Enlargement, and Hippocampal Atrophy.
Alorf *et al.* [357]	2022	AD vs. LMCI vs. MCI vs. EMCI vs. SMC vs. CN (33 AD, 38 LMCI, 6 MCI, 54 EMCI, 23 SMC, 50 CN subjects)	ADNI	rs-fMRI	BC-GCN	Multi-class (6 stages) & Binary; Validation: 10-fold CV (SSAE) and 5-fold CV (BC-GCN)	CN vs. SMC 94.93% Acc., CN vs. EMCI 91.45% Acc., CN vs. MCI 97.03% Acc., CN vs. LMCI 91.73% Acc., CN vs. AD 94.17% Acc. Multi-class Accuracy: 84.03%.	4005 Connectivity Features: Pearson correlation coefficients (upper triangular matrix) between 90 AAL ROIs. Key regions: Significant weights identified in the precentral gyrus, frontal gyrus (middle/superior), lingual gyrus, and supplementary motor area.

Continued on next page

Table S2 – *continued from previous page*

Reference	Year	Comparison Groups	Database	Data Type	Best Classifier	Validation	Best Result	Features Used
Chatterjee *et al.* [381]	2022	AD vs. Non-AD (78 Demented, 72 Nondemented)	OASIS	MRI	Proposed Voting Ensemble Model (Hard voting combining LR, NB, KNN, and SVM)	5-fold cross-validation; 70/30 train/test split	96.43% Accuracy; 0.973 AUC	12 key features including: Biographical data (Age, Gender/Sex), clinical scores (MMSE, Socioeconomic Status (SES), Education (EDUC), CDR), and MRI-derived measures (Estimated Total Intracranial Volume (eTIV), Normalized Whole Brain Volume (nWBV), and Atlas Scaling Factor (ASF))
Huang and Qiu *et al.* [439]	2024	ADNI: NC vs. AD (546 NC, 592 AD); NC vs. MCI (546 NC, 910 MCI); MCI vs. AD (910 MCI, 592 AD). OASIS-3: NC vs. AD (712 NC, 274 AD); NC vs. MCI (712 NC, 102 MCI); MCI vs. AD (102 MCI, 274 AD)	ADNI and OASIS-3	MRI	Monte Carlo Vision Transformer Ensemble (MC-ViT)	Hold-out: 37.5% training, 12.5% validation, and 50% test (evaluation) set; repeated 5 times	ADNI (NC vs. AD): 90.14% Balanced Accuracy; AUC 0.9557. OASIS-3 (NC vs. AD): 80.28% Balanced Accuracy; AUC 0.8632	3D T1-weighted sMRI patches: 64 total patches initially (128x119x155 brain cropped size), uniformly divided into 50x50x50 cubic patches. Latent Hierarchical Features: 400 features per patch flattened into a vector; 32 patches sampled via Monte Carlo distribution based on patch accuracy. Anatomical ROIs: Focus on discriminative regions including the Hippocampus, gray matter, and sulcus gyrus edges.
**Studies with PET**								

Continued on next page

Table S2 – *continued from previous page*

Reference	Year	Comparison Groups	Database	Data Type	Best Classifier	Validation	Best Result	Features Used
Matho-taarachchi *et al.* [5]	2017	pMCI vs. sMCI (43 pMCI, 230 sMCI)	ADNI	Amyloid PET, MRI	RUSRF (Random Under Sampling Random Forest)	10-fold nested cross-validation (Independent test set)	pMCI vs. sMCI: 84% Acc., 70.8% Sen., 86.5% Spe., 0.91 AUC (Independent test set).	Multi-modal Features: Regional PET SUVR intensities from 9 brain regions (Temporal Occipital Junction, Mid Temporal Cortex, Mid Frontal Cortex, Precuneus, PCC, Orbitofrontal Cortex, Putamen, Nucleus Accumbens, Angular Gyrus), Age, Gender, and APOE4 genotype.
Choi *et al.* [387]	2018	AD vs. MCI vs. NC (139 AD, 171 MCI, 182 NC); MCI converters (79) vs. nonconverters (92)	ADNI-2	FDG-PET, AV-45 PET	Deep CNN	10-fold cross-validation	AD vs. NC: 96.0% Acc. (0.98 AUC); MCI conv. vs. non-conv: 84.2% Acc. (AUC 0.89)	Voxel-based: Raw 3D PET volumes (all voxels). VOI-based: Mean FDG uptake in angular, temporal, and posterior cingulate cortices. Mean AV-45 uptake in frontal, anterior/posterior cingulate, lateral parietal, and lateral temporal regions. Reference regions: pons/ cerebellar vermis (FDG) and whole cerebellum (AV-45).

Continued on next page

Table S2 – *continued from previous page*

Reference	Year	Comparison Groups	Database	Data Type	Best Classifier	Validation	Best Result	Features Used
M. Liu *et al.* [489]	2018	AD vs. NC (93 AD, 100 NC); MCI vs. NC (146 MCI, 100 NC)	ADNI	FDG-PET	Weighted Fusion of multi-view CNN-BGRU models	10-fold cross-validation	AD vs. NC: 91.2% ACC (91.4% sens., 91.0% spec., 95.3% AUC). MCI vs. NC: 78.9% ACC (78.1% sens., 80.0% spec., 83.9% AUC).	Intra-slice and Inter-slice Context Features: Sequential 2D image slices (decomposed from 3D FDG-PET in axial, sagittal, and coronal views). Each group contains 15 slices. Features represent regional glucose metabolic activity focused in AD-vulnerable areas like the hippocampus, posterior temporal lobe, and parietal lobe.
van Veen *et al.* [580]	2018	HC vs. PD vs. AD (CUN: 19 HC, 68 PD; UMCG: 19 HC, 21 AD; UGOSM: 44 HC, 55 AD)	CUN, UMCG, and UGOSM clinical datasets	FDG-PET	LGMLVQ	10 randomized realizations of 10-fold cross-validation	HC vs. AD (UGOSM): 0.99 AUC (SVM best). HC vs. PD (UGOSM): 0.84 AUC (GMLVQ). PD vs. AD (UGOSM): 0.95 AUC (LGMLVQ); Center Identification (HC vs. PD data): CUN vs. UGOSM (0.99 AUC), UGOSM vs. UMCG (0.99 AUC), UMCG vs. CUN (1.00 AUC).	Subject Scores: 36-dimensional projected metabolic patterns (PDRP for Parkinson's and ADRP for Alzheimer's). Obtained via Log-transform, centering, and inner product projection of MNI-normalized images onto a Group Invariant Subprofile (GIS) matrix.

Continued on next page

Table S2 – *continued from previous page*

Reference	Year	Comparison Groups	Database	Data Type	Best Classifier	Validation	Best Result	Features Used
De Santi *et al.* [392]	2022	AD vs. MCI vs. CN (486 AD, 1148 MCI, 918 CN scans)	ADNI	FDG-PET	3D CNN	Multi-class (CN, MCI, AD); Validation: 5-fold CV (Subject-level split: 80% train+val / 20% test)	CN vs. All: 74% Acc., AUC 0.81; MCI vs. All: 59% Acc., AUC 0.63; AD vs. All: 78% Acc., AUC 0.77.	Regional Metabolism: Average voxel intensities in brain lobes. Highly ranked ROIs: Frontal-temporal space (most important for SM), Occipital lobe (most important for LRP), Sub-lobar, Temporal lobe, Limbic lobe, and Thalamus.
García-Gutierrez *et al.* [414]	2022	AD vs. bvFTD vs. HC (171 AD, 72 bvFTD, 87 HC)	Hospital Clinico San Carlos (Spain)	FDG-PET, cognitive test data	Meta-model with Evolutionary Grammars (EG)	Multi-class and Binary; Validation: 5-fold CV (stratified); Independent validation set	AD+FTD vs. HC (Best condition: PET+Cog): 0.926 Acc; 0.949 F1; 0.966 Prec; 0.933 Sens; 0.906 Spec. One-vs-Rest (AD): 0.854 Acc (Grammar); 0.829 Acc (Bayesian).	Heterogeneous Features: (1) Demographics: age, gender, education. (2) Cognitive: ACE-III (memory, executive, etc.), TMT-A/B, FCSRT, ROCF. (3) Metabolism: Hypometabolic voxels in Brodmann (ROIs 35, 37, 47) and AAL (f1m_l, pcl_r, sma_l, put_r, cau_l) atlases.
**Multi-modal studies**								
Yu *et al.* [599]	2016	AD vs. MCI vs. NC (50 AD, 97 MCI, 52 NC)	ADNI	MRI, FDG-PET, CSF	Graph-guided Multi-Task Learning (GGML)	10-fold cross-validation repeated 30 times	AD vs. NC: 92.6% Acc. (combined); MCI vs. NC: 80.0% Acc. (combined)	189 Features: 93 gray matter volumes (MRI), 93 average intensities for ROI (PET), and 3 CSF features (Aβ42, t-tau, p-tau). Multi-task targets: Class label, MMSE score, and ADAS-Cog score.

Continued on next page

Table S2 – *continued from previous page*

Reference	Year	Comparison Groups	Database	Data Type	Best Classifier	Validation	Best Result	Features Used
Zu *et al.* [612]	2016	AD vs. MCI vs. NC (51 AD, 99 MCI, 52 NC)	ADNI	MRI, FDG-PET	Label-aligned MTFS + MK-SVM	10-fold cross-validation repeated 10 times	AD vs. NC: 95.95% Acc. (95.10% sens., 96.54% spec., 0.97 AUC); MCI vs. CN: 80.26% Acc. (84.95% sens., 70.77% spec., 0.81 AUC)	186 Features: Grey matter tissue volume from 93 manually labeled ROIs (MRI) and average intensity from the corresponding 93 matching ROIs in FDG-PET images aligned via rigid transformation.
Suk *et al.* [561]	2016	AD vs. MCI vs. CN (51 AD, 99 MCI [43 pMCI, 56 sMCI], 52 CN)	ADNI	MRI, FDG-PET, CSF	Deep Weighted Subclass-based Sparse Multi-Task Learning (DW-S2MTL)	10-fold cross-validation	AD vs. CN: 95.09% Acc. (Combined, DW-S2MTL); MCI vs. CN: 80.11% Acc. (MRI + PET, DW-S2MTL); pMCI vs. sMCI: 74.15% Acc. (MRI + PET, DW-S2MTL)	189 Multi-modal features: 93 ROI gray matter volumes (MRI); 93 mean ROI intensities (PET); and 3 CSF biomarkers (Aβ42, t-tau, p-tau). Key ROIs: hippocampal formation, amygdala, and precuneus.
Schouten *et al.* [544]	2016	AD vs. HC (77 AD, 173 HC); includes Mild AD (39) and Moderate AD (38)	PRODEM (Medical University of Graz) and Austrian Stroke Prevention Family Study	MRI, DWI, rs-fMRI	Stepwise Multi-modal combination	10-fold nested cross-validation (repeated 50 times)	AD vs. HC: AUC 0.952 (82.6% sens., 92.7% spec., 89.6% accuracy); Mild AD vs. CN: AUC 0.934 (72.1% sens., 93.5% spec., 89.6% Acc.); Moderate AD vs. CN: 0.971 AUC (81.3% sens., 95.6% spec., 93.0% Acc.)	Grey matter density (GMD): Averaged values from 110 Harvard-Oxford probabilistic ROIs. White matter density (WMD): Averaged from 20 JHU tractography ROIs. DTI: Mean FA and MD values for the 20 JHU regions. Connectivity: 2415 sparse L1-regularized partial correlations between 70 ICA components.

Continued on next page

Table S2 – *continued from previous page*

Reference	Year	Comparison Groups	Database	Data Type	Best Classifier	Validation	Best Result	Features Used
Möller *et al.* [505]	2016	AD vs. HC vs. bvFTD (84 AD, 94 HC, 51 bvFTD)	VU University Medical Center Amsterdam and Erasmus University Medical Center Rotterdam	MRI	Linear SVM	Independent training (n=115) and prediction (n=114) sets; training used LOOCV	AD vs. HC: AUC 0.95 (88% Accuracy); bvFTD vs. HC: AUC 0.87 (85% Accuracy); AD vs. bvFTD: AUC 0.81 (82% Accuracy)	Voxel-wise Gray Matter (GM) density maps: Whole-brain maps corrected for individual brain size by rigid-body coregistration to the template. Comparison features included neuropsychological Z-scores for memory, language, attention, and executive function.
Tang *et al.* [565]	2016	AD vs. HC (29 AD, 23 HC; sub-cohort 22 AD, 15 HC for combined results)	Tongji Hospital (Wuhan, China)	MRI, DTI	SVM combining shape and DTI features	LOOCV	AD vs. CN: Right Hippocampus (Shape + DTI): 94.6% Accuracy (95.5% sens., 93.3% spec.) (PCA + ttest)	Morphometrics: Global volumes and vertex-based shape markers (log-determinant of Jacobian). DTI Indices: Mean Fractional Anisotropy (FA) and Mean Trace (3x MD) within the hippocampus and amygdala.
Ferreira *et al.* [409]	2017	AD vs. Controls (20 AD, 18 Controls)	Memory outpatient clinics (USP, Brazil)	MRI, FDG-PET, rCBF-SPECT	SVM (T1-MRI + FDG-PET with cerebellar normalization)	LOOCV	AD vs. Controls: AUC 0.82 (75% sens., 72.22% spec.)	Voxel-based: Feature vectors containing 219,727 brain voxels. Includes Grey Matter volume (MRI), regional cerebral metabolism (PET), and regional blood flow (SPECT).

Continued on next page

Table S2 – *continued from previous page*

Reference	Year	Comparison Groups	Database	Data Type	Best Classifier	Validation	Best Result	Features Used
Lei *et al.* [477]	2016	AD vs. NC (93 AD, 101 NC); MCI vs. NC (204 MCI, 101 NC); MCI-C vs. MCI-NC (76 MCI-C, 128 MCI-NC)	ADNI	MRI, FDG-PET	SVM with Hybrid Level Fusion (MRI+ PET+HF)	10-fold cross-validation	AD vs. NC: 96.93% Acc. (AUC 95.7); MCI vs. CN: 86.57% Acc. (AUC 82.03); MCI-C vs. MCI-NC: 82.75% Acc. (AUC 85.27)	MRI & PET: Grey Matter intensities (MRI) and PET intensities represented by dense SIFT descriptors. Encoded into BoVW/VLAD histograms (high-level representations) integrating multi-scale spatial information.
Zhu *et al.* [611]	2016	AD vs. NC (51 AD, 52 NC); MCI-C vs. MCI-NC (43 MCI-C, 56 MCI-NC); 3-class: AD vs. MCI vs. NC (51 AD, 99 MCI, 52 NC)	ADNI	MRI, FDG-PET	Proposed Subspace Regularized Sparse Multi-Task Learning + SVM (MRI+PET)	10-fold cross-validation	AD vs. NC: 95.5% Acc.; MCI vs. CN: 79.7% Acc.; MCI-C vs. MCI-NC: 71.2% Acc.	ROI-based: 93 ROIs labeled via Jacob template. For MRI, Grey Matter tissue volumes. For PET, average FDG uptake intensity in each of the 93 ROIs.

Continued on next page

Table S2 – *continued from previous page*

Reference	Year	Comparison Groups	Database	Data Type	Best Classifier	Validation	Best Result	Features Used
Tong *et al.* [570]	2017	AD vs. MCI vs. NC (37 AD, 75 MCI, 35 NC)	ADNI	MRI, FDG-PET, CSF, genetics	non-linear Graph Fusion (NGF) with RF	leave-25%-out	AD vs. NC: AUC 98.3%, Acc 91.8% (Sens 88.9%, Spec 94.7%); MCI vs. NC: AUC 81.2%, Acc 79.5% (Sens 85.1%, Spec 67.1%); 3-way: AUC 72.9%, Acc 60.2%	Total features: 239,391. MRI (83): Volumetric region-based features normalized by intracranial volume. PET (239,304): Voxel-wise signal intensities within the brain mask. CSF (3): Levels of Aβ42 (amyloid plaques marker), T-tau (axonal damage), and P-tau (neurofibrillary tangles). Genetics (1): Categorical APOE genotype (ϵ2/ϵ3,ϵ2/ϵ4,ϵ3/ϵ3,ϵ3/ϵ4,ϵ4/ϵ4).
Zheng *et al.* [608]	2017	AD vs. HC (51 AD, 52 HC)	ADNI	MRI, FDG-PET (as PI)	Ensemble LUPI (SVM+-RBM+)	10-fold	AD vs. HC: Acc 88.52% (Spec 92.20%, AUC 0.943)	MRI (93): Grey matter tissue volumes extracted from 93 anatomical Regions of Interest (ROI) partitioned via atlas warping. PET (93): Average signal intensities calculated per ROI for the same 93 regions (used as Privileged Information during training).

Continued on next page

Table S2 – *continued from previous page*

Reference	Year	Comparison Groups	Database	Data Type	Best Classifier	Validation	Best Result	Features Used
Ahmed *et al.* [348]	2017	AD vs. NC vs. MCI (45 AD, 52 NC, 58 MCI)	ADNI	MRI, DTI (MD maps)	MKL (MRI + MD + CSF)	10-fold cross-validation	AD vs. NC: 90.2% Acc., 82.92% Sen., 97.2% Spec.; MCI vs. NC: 79.42% Acc., 71.58% Sen., 86.05% Spec.; AD vs. MCI: 76.63% Acc., 65.62% Sen., 81.33% Spec.	Multi-modal Hippocampal Signatures: Local image-derived biomarkers from T1-weighted MRI (structural CHFs), DTI Mean Diffusivity (MD) signal variation CHFs, and hippocampal CSF volume computed via adaptive thresholding.
Chen *et al.* [382]	2017	AD vs. NC (27 AD, 26 NC)	Tianjin First Central Hospital	MRI, DTI, DKI	SVM-RFE with All-DKI indices	Nested cross-validation (Outer LOOCV)	AD vs. NC: 96.23% Acc., 100% Sen., 92.86% Spe., 0.992 AUC.	DKI and DTI Indices: 23 ROIs across the brain. Diffusivity indices (FA, MD, AxD, RD) and Kurtosis indices (AK, RK, MK). Best result achieved by combining all DKI-derived diffusivity and kurtosis measurements.
Ortiz *et al.* [517]	2016	AD vs. NC, stable MCI (MCIs) vs. AD, NC vs. MCI converters (MCIc) (70 AD, 68 NC, 64 MCIs, 39 MCIc)	ADNI	MRI (GM), FDG-PET	FEDBN-SVM L2 (DBN features fused by SVM)	10-fold stratified cross-validation	NC vs. AD: 90% Accuracy (AUC 0.95); NC vs. MCIc: 83.3% Accuracy (AUC 0.95); NC vs. MCIs: 80% Acc. (AUC 0.84); MCIs vs. AD: 84% Acc. (AUC 0.90); MCIc vs. AD: MCIs vs. MCIc: 78.0% Acc. (AUC 0.82)	Voxel-as-Features (AAL ROIs): GM and FDG-PET activations from 98 regions. Features used are activations from DBN hidden layer 2 (400 features/region), encoding different abstraction levels.

Continued on next page

Table S2 – *continued from previous page*

Reference	Year	Comparison Groups	Database	Data Type	Best Classifier	Validation	Best Result	Features Used
Hor & Moradi [435]	2016	CN vs. pMCI (178 NL, 96 pMCI); sMCI vs. AD (126 sMCI, 108 AD); sMCI vs. pMCI (126 sMCI, 96 pMCI)	ADNI	MRI, FDG/ AV45 PET	Scandent tree Multi-modal feature transform forest	5-fold cross-validation	sMCI vs. AD: AUC 0.892 (Acc. 0.795); CN vs. pMCI: AUC 0.837 (Acc. 78.8%); sMCI vs. pMCI: AUC 0.872 (81.5% Acc.)	MRI: Volume measurements of 6 ROIs (ventricles, hippocampus, whole-brain, entorhinal, fusiform, and mid-temporal) and intracranial volume (ICV) in mm3. PET: Uptake values for FluoroDeoxyGlucose (FDG) and AV45.
Cheng *et al.* [384]	2017	AD vs. NC (93 AD, 100 NC)	ADNI	MRI, FDG-PET	Multi-modality Cascaded CNN	10-fold cross-validation	AD vs. NC: 89.64% Acc., 87.10% Sens., 92.00% Spec., 94.45% AUC	Fused compact high-level features from sMRI (Gray Matter density maps) and FDG-PET scans; extracted as 3D patches of 49x39x38 voxels (downsampled from original 98x78x76 images).
Vu *et al.* [582]	2017	AD vs. NC (145 AD, 172 NC); MCI vs. NC	ADNI	MRI, PET	SAE + CNN (PET-MRI Fusion)	954/127/191 Train/Val/Test examples	AD vs. NC: 91.14% Accuracy; MCI vs. NC: 89.1% Accuracy	3D patches (5x5x5 voxels) extracted from co-registered T1-weighted sMRI and FDG-PET scans; full scan dimensions are 79x95x68 (510,340 voxels).

Continued on next page

Table S2 – *continued from previous page*

Reference	Year	Comparison Groups	Database	Data Type	Best Classifier	Validation	Best Result	Features Used
Wang *et al.* [586]	2018	AD vs. aMCI vs. NC (35 AD, 30 aMCI, 40 NC)	Beijing Xuanwu Hospital	fMRI, DTI	Multi-modal CNN	80/20 train-test split	Comprehensive 3-class (AD, aMCI, NC): 92.06% Accuracy (DTI + fMRI).	Brain Network Matrices: Functional Connectivity Network (FCN) images and DTI Structural Connectivity Network (DTISCN) images (90x90) used as input for spatial correlation learning in CNN.
Xiao *et al.* [594]	2017	AD vs. MCI vs. NC (54 AD, 58 MCI, 58 NC)	ADNI	MRI	Proposed SVM with RFE+ Covariance selection	10-fold cross-validation	AD vs. NC: 92.86% Acc., 98.28% Spe.; MCI vs. NC: 97.22% Acc., 100% Spe., 100% PPV; AD vs. MCI: 91.18% Acc., 100% Sen.; 3-way (Multi-class): 85.59% Acc..	292 Multifeature Fusion Features: (1) 8 Morphological (3D) features: Gray-matter volume from 8 clusters identified via VBM (e.g., Hippocampus, Frontal Lobe, Pons, Cerebellum). (2) 284 Texture (2D Variation) features: 220 GLCM features (11 statistics across 20 matrices covering 5 distances and 4 directions) and 64 Gabor features (mean and variance of amplitude from 32 filters responsive to frequencies and directions).

Continued on next page

Table S2 – *continued from previous page*

Reference	Year	Comparison Groups	Database	Data Type	Best Classifier	Validation	Best Result	Features Used
Aderghal *et al.* [346]	2018	AD vs. MCI vs. NC (48 AD, 108 MCI, 58 NC)	ADNI	MRI, DTI (MD maps)	Fusion (Majority Vote) of CNNs, 2D+epsilon late fusion classification	Train/Test/ Validation	AD vs. NC: 92.5% Acc., 94.7% Sen., 90.4% Spe., 92.5% BAC. AD vs. MCI: 85.0% Acc., 93.7% Sen., 79.1% Spe., 86.4% BAC. MCI vs. NC: 80.0% Acc., 92.8% Sen., 73.0% Spe., 82.9% BAC.	Hippocampal ROI 2D+epsilon Images: 3-channel images (median slice + neighbors) extracted from ROI subvolumes (28x28x28) along Sagittal, Axial, and Coronal projections.
Ortiz *et al.* [518]	2018	AD vs. MCI vs. CN (70 AD, 111 MCI, 68 CN)	ADNI	FDG-PET, MRI (GM)	Multi-modal SR-SVC Combination	10-fold cross-validation	CN vs. AD: 92% Acc., 94% Sen., 89% Spe., 0.96 AUC. CN vs. MCI: 79% Acc., 85% Sen., 71% Spe., 0.82 AUC.	Sparse Features: Sparse representation coefficients extracted from functional (PET uptake) and structural (MRI GM probability) data using learned atoms (30 atoms) that capture class-specific variance.
Shi *et al.* [552]	2018	AD vs. MCI vs. NC (51 AD, 99 MCI [43 MCI-C, 56 MCI-NC], 52 NC)	ADNI	MRI, FDG-PET	MM-SDPN with SVM	10-fold CV (repeated 5 times)	AD vs. NC: 97.13% Acc, 0.972 AUC; MCI vs. NC: 87.24% Acc, 0.901 AUC; MCI-C vs. MCI-NC: 78.88% Acc, 0.801 AUC; Multiclass (4 groups): 57.00% Acc	186 ROI-based features representing the whole brain. This includes 93 Gray Matter volumes extracted from T1-MRI and 93 average metabolic intensities extracted from FDG-PET scans aligned to the MRI space. ROI partitioning was based on a manually labeled 93-region template.

Continued on next page

Table S2 – *continued from previous page*

Reference	Year	Comparison Groups	Database	Data Type	Best Classifier	Validation	Best Result	Features Used
Vu *et al.* [583]	2018	AD vs. MCI vs. NC (193 AD, 215 MCI, 207 NC scans)	ADNI	MRI, FDG-PET	HiLCAE + 3D-VGG16	Random split (80/10/10 test), repeated 5 times	AD vs. NC: 98.8% Acc; MCI vs. NC: 95.0% Acc; AD vs. MCI: 93.0% Acc; Multiclass (AD/MCI/NC): 91.13% Acc	3D non-white matter (non-WM) PET scans capturing pure gray matter regions. Features are standardized to standardized volumes of 79 $\times$ 95 $\times$ 79 voxels. Unsupervised learning utilized randomly selected 3D patches of size 25x25x25 extracted from these processed scans.
Lu *et al.* [496]	2018	sNC vs. pNC vs. sMCI vs. pMCI vs. sAD (360 sNC, 18 pNC, 409 sMCI, 217 pMCI, 238 sAD)	ADNI	MRI, FDG-PET	MMDNN (Ensemble of 10 networks)	10-fold CV (90/10 Train/ Test)	sMCI vs. pMCI: 82.93% Acc; sNC vs. (pNC/ pMCI/sAD): 86.4% Acc, 86.5% Sens, 86.3% Spec; sAD Identification: 94.25% Acc; 1-year Conversion (pMCI): 90% Acc	Multiscale patch-wise features extracted at 3 hierarchical scales: 500, 1000, and 2000 voxels. This resulted in feature dimensions of 1488, 705, and 343 patches for each scale respectively. Features include patch volumes (from MRI) and mean metabolic intensities normalized by brainstem reference (from PET).

Continued on next page

Table S2 – *continued from previous page*

Reference	Year	Comparison Groups	Database	Data Type	Best Classifier	Validation	Best Result	Features Used
Lu *et al.* [497]	2018	NC vs. AD (304 NC, 226 AD); sMCI vs. pMCI (409 sMCI, 112 pMCI)	ADNI	FDG-PET, MRI	MDNN with instance-transfer learning	Ensemble of 10 networks; 10-fold cross-validation	NC vs. AD: 93.58% Acc. sMCI vs. pMCI: 81.55% Acc. sMCI vs. pMCI (+transf.): 82.51% Acc.	Multiscale patch-wise metabolism: Signal extracted from 85 FreeSurfer-defined anatomical ROIs. Features harvested at three spatial scales using patches of 500, 1000, and 2000 voxels, resulting in 1488, 705, and 343 features respectively. Intensities normalized to the mean brainstem value.
Kim *et al.* [463]	2018	AD vs. MCI vs. HC (51 AD, 99 MCI, 52 HC)	ADNI1	MRI (1.5T), FDG-PET, CSF	Multi-modal sparse hierarchical extreme learning machine (MSH-ELM (ELM_RBF))	10-fold cross-validation	AD vs. HC: 97.12% Acc. (BAC 96.10%). MCI vs. HC: 87.09% Acc. (BAC 83.46%)	Multi-modal features (189 total): MRI: Gray matter (GM) volumes from 93 ROIs. PET: Mean GM intensities from 93 ROIs (aligned to MRI). CSF: 3 biomarkers (Aβ42, t-tau, p-tau).

Continued on next page

Table S2 – *continued from previous page*

Reference	Year	Comparison Groups	Database	Data Type	Best Classifier	Validation	Best Result	Features Used
Cheng *et al.* [386]	2019	AD vs. MCI vs. NC (102 AD, 192 MCI, 112 NC); PMCI (86) vs. SMCI (106)	ADNI1	MRI, CSF	2S-rMLTFL (Two-stage) with linear SVM	10-fold cross-validation repeated 10 times	AD vs. NC: 95.2% Acc. (AUC 0.983). MCI vs. NC: 82.4% Acc. (AUC 0.865). PMCI vs. SMCI: 76.3% Acc. (AUC 0.810). AD vs. MCI: 76.7% Acc. (AUC 0.820). SMCI vs. NC: 74.3% Acc. (AUC 0.808)	Multi-modal Features: MRI: GM volumes of 93 ROIs based on the Jacob/Kabani template. CSF: Aβ42, t-tau, and p-tau. Top discriminative features: t-tau, p-tau, Amygdala, Hippocampal formation, Entorhinal cortex, Uncus, Perirhinal cortex, and Middle Frontal Gyrus.

Continued on next page

Table S2 – *continued from previous page*

Reference	Year	Comparison Groups	Database	Data Type	Best Classifier	Validation	Best Result	Features Used
Varatharajah *et al.* [42]	2019	MCI-P (Progressive) vs. MCI-NP (Non-progressive) (39 MCI-P, 96 MCI-NP)	ADNI	MRI, PET, CSF, genetic, cognitive, demographic	Support Vector Machine (SVM) with Linear Kernel	5-fold cross-validation (outer loop); 10-fold CV (inner loop for optimization) (80% train, 20% test)	Short-term MCI-to-AD progression: 0.93 AUC (93% sens., 77% spec., 81% ACC).	94 potential factors: CSF (Aβ42, T-tau, P-tau); sMRI (Standard ROI volumes and thickness scaled by total intracranial volume, and hippocampal subfield volumes); PET (FDG-PET SUVR in temporal, angular, and posterior cingulate gyri; F-florbetapir regional SUVR in temporal, parietal, and cingulate cortex, plus global measure); Cognition (MMSE scores); Cognitive Resilience (ANART error count, years of education); Genetics (APOE ε4 status and expression data for BIN1, CLU, ABCA7, CR1, PICALM, MS4A6A, CD33, CD2AP); Demographics (Age, gender).

Continued on next page

Table S2 – *continued from previous page*

Reference	Year	Comparison Groups	Database	Data Type	Best Classifier	Validation	Best Result	Features Used
Forouzan-nezhad *et al.* [410]	2018	AD vs. LMCI vs. EMCI vs. CN (52 AD, 110 LMCI, 110 EMCI, 112 CN)	ADNI	MRI, AV-45 PET, clinical scores	Deep Neural Network (DNN)	10-fold cross-validation	CN vs. AD (+ NTS): 0.98 AUC. CN vs. LMCI (+ NTS): 0.91 AUC. CN vs. EMCI (+ NTS): 0.90 AUC	Multi-modal Feature Set: MRI features (ROI volumes and cortical thickness); AV45 PET features (Regional and global SUVR); NTS (Neuropsychological Scores) including RAVLT (Immediate and delayed recall), MoCA, and ECogT.
Gupta *et al.* [424]	2019	AD vs. MCIs vs. MCIc vs. HC (38 AD, 36 MCIs, 46 MCIc, 38 HC)	ADNI	MRI, FDG-PET, CSF, APOE genotype	RBF kernel-based Multi-class SVM	10-fold stratified cross-validation (repeated 100 times)	AD vs. HC: 98.42% ACC (100% sens., 96.47% spec., 98.33 AUC). MCIs vs. MCIc: 94.86% ACC (100% sens., 88.71% spec., 93.59 AUC). AD vs. MCIs: 96.65% ACC (100% sens., 91.67% spec., 96.83 AUC). AD vs. MCIc: 92.26% ACC (91.67% sens., 92.86% spec., 94.64 AUC). HC vs. MCIc: 94.13% ACC (94.75% sens., 100% spec., 96.43 AUC). HC vs. MCIs: 95.65% ACC (100% sens., 88.89% spec., 95.24 AUC).	497 Integrated Features: 246 sMRI ROIs (Gray Matter volumes); 246 FDG-PET ROIs (average intensities) extracted using the Brainnetome atlas. 3 CSF protein levels (t-tau, p-tau181p, and Aβ42). 2 APOE Genotype features (allele pairs).

Continued on next page

Table S2 – *continued from previous page*

Reference	Year	Comparison Groups	Database	Data Type	Best Classifier	Validation	Best Result	Features Used
Feng *et al.* [407]	2018	AD vs. NC (93 AD, 100 NC); pMCI vs. NC (76 pMCI, 100 NC); sMCI vs. NC (128 sMCI, 100 NC)	ADNI	MRI, FDG-PET	Dual 3D-CNN + SBi-RNN	10-fold evaluation (repeated 10 times)	AD vs. NC: 94.29% ACC (96.59% sens., 92.38% spec., 0.962 AUC). pMCI vs. NC: 84.66% ACC (83.56% sens., 85.44% spec., 0.896 AUC). sMCI vs. NC: 64.47% ACC (70.43% sens., 48.41% spec., 0.671 AUC).	Joint Multi-modal Feature Map: MRI-derived Gray Matter (GM) density maps and PET-derived glucose metabolic intensity images, both downsampled to 64x64x64 voxels. Features are extracted from the last convolutional layer, cascaded, and flattened into a 1D 2D feature vector for sequential analysis.
Liu *et al.* [199]	2018	AD vs. NC (93 AD, 100 NC); pMCI vs. NC (76 pMCI, 100 NC); sMCI vs. NC (128 sMCI, 100 NC)	ADNI	MRI, FDG-PET	Multi-Modality Cascaded CNNs	Ten-fold cross-validation	AD vs. NC: Acc 93.26%, AUC 95.68%. pMCI vs. NC: Acc 82.95%, AUC 88.43%. sMCI vs. NC: Acc 64.04%, AUC 67.05%. MCI (total) vs. NC: Acc 74.34%, AUC 80.23%.	Local Multi-Modality: 27 overlapping 3D image patches (size 50×41×40) from both MRI and PET at same positions. Correlational: Latent Multi-modal correlation features capturing structural (MRI) and metabolic (PET) activities.

Continued on next page

Table S2 – *continued from previous page*

Reference	Year	Comparison Groups	Database	Data Type	Best Classifier	Validation	Best Result	Features Used
Feng *et al.* [408]	2019	AD vs. NC (93 AD, 100 NC); pMCI vs. NC (76 pMCI, 100 NC); sMCI vs. NC (128 sMCI, 100 NC)	ADNI	MRI, FDG-PET	3D-CNN and Fully Stacked Bidirectional LSTM (FSBi-LSTM, 2 layers)	10-fold CV	AD vs. NC: 94.82% Acc. (97.70% sens., 92.45% spec., AUC 0.9676); pMCI vs. NC: 86.36% Acc. (83.33% sens., 88.78% spec., AUC 0.9111); sMCI vs. NC: 65.35% Acc. (70.59% sens., 59.63% spec., AUC 0.6917)	Voxel-based Imaging: Gray Matter (GM) masks extracted from T1-MRI and metabolic data from FDG-PET. Deep Features: 200 high-level semantic and spatial feature vectors extracted from 3D-CNN feature maps (dimension 2×2×2) representing specific part-by-part brain structure information.
Huang *et al.* [438]	2019	AD vs. pMCI vs. sMCI vs. CN (647 AD, 326 pMCI, 441 sMCI, 731 CN)	ADNI	MRI, FDG-PET	3D-CNN Multi-modality Protocol B1 (CN vs. AD) and Protocol B2 (MCI tasks)	70% train / 10% validation / 20% test split	CN vs. AD: 90.10% Acc. (90.85% sens., 89.21% spec., AUC 0.9084); CN vs. pMCI: 87.46% Acc. (90.73% sens., 80.61% spec., AUC 0.8761); sMCI vs. pMCI: 76.90% Acc. (68.15% sens., 83.93% spec., AUC 0.7961)	Hippocampal Patches: Cropped 3D ROI patches (96×96×48 voxels) centred on the hippocampi. Features capture hippocampal shape, volume, texture, and regional glucose uptake (metabolic activity) from surrounding tissues.

Continued on next page

Table S2 – *continued from previous page*

Reference	Year	Comparison Groups	Database	Data Type	Best Classifier	Validation	Best Result	Features Used
Peng *et al.* [524]	2019	Dataset I: AD vs. MCI vs. NC (49 AD, 93 MCI, 47 NC); Dataset II: AD vs. MCI vs. NC (85 AD, 185 MCI, 90 NC)	ADNI	MRI, PET, genetics	Proposed $\ell_{1,p}$-norm regularized Multiple Kernel Learning (MKL)	10-fold CV (repeated 10 times); 5-fold inner CV for parameter tuning	Dataset I: AD vs. NC: 96.1% Acc. (97.3% sens., 94.9% spec., AUC 0.992); Dataset I: MCI vs. NC: 80.3% Acc. (85.6% sens., 69.8% spec., AUC 0.811); Dataset I: AD vs. MCI: 76.9% Acc. (65.9% sens., 82.7% spec., AUC 0.808); Dataset II: AD vs. NC: 94.5% Acc. (AUC 0.98); Dataset II: MCI vs. NC: 80.2% (AUC 0.82); Dataset II: AD vs. MCI: 80.1% (AUC 0.83)	MRI: Gray Matter (GM) volumes of 93 ROIs. PET: Average intensity (glucose metabolism) of 93 ROIs. Genetics: Single Nucleotide Polymorphisms (SNPs) belonging or proximal to top 135-153 AD genes (e.g., rs429358 and rs769449 from ApoE; rs3025035 from VEGFA; rs822097 from SORCS1).
Lee *et al.* [476]	2019	MCI-C vs. MCI-NC (307 MCI-C, 558 MCI-NC); Auxiliary: AD vs. CN (338 AD, 415 CN)	ADNI	MRI, CSF, cognitive, demographic (longitudinal)	Multi-modal Recurrent Neural Network (GRU) with L1-regularized Logistic Regression	5-fold CV (run 10 times)	MCI-C vs. MCI-NC (6m): 81% Acc. (84% sens., 80% spec, AUC 0.86); MCI-C vs. MCI-NC (12m): 81% Acc. (84% sens., 80% spec, AUC 0.86); MCI-C vs. MCI-NC (18m): 79% Acc. (82% sens., 79% spec); MCI-C vs. MCI-NC (24m): 80% Acc. (81% sens., 80% spec.)	Demographics: Age, sex, years of education. Genetics: APOE $\varepsilon 4$ status (carrier/non-carrier). Cognitive (Longitudinal): ADNI-EF (Executive Function) and ADNI-MEM (Memory) composite scores. CSF (Longitudinal): Amyloid-β 1–42 (Aβ1–42), total tau (t-tau), and threonine 181 phosphorylated tau (p-tau). MRI: Hippocampal volume and entorhinal cortical thickness.

Continued on next page

Table S2 – *continued from previous page*

Reference	Year	Comparison Groups	Database	Data Type	Best Classifier	Validation	Best Result	Features Used
Lee *et al.* [475]	2019	MCI-C vs. MCI-NC (200 MCI-C, 401 MCI-NC)	ADNI	MRI, CSF, cognitive, demographic (longitudinal)	MildInt (Multi-modal GRU integration with Logistic Regression)	5-fold CV (run 10 times)	MCI-C vs. MCI-NC: 79% Acc. (83% sens., 77% spec.,)	Demographics: Age, sex, level of education. Longitudinal Cognitive: Clinical assessment scores (memory/executive). Longitudinal CSF: 5 numerical features including Aβ1–42 and tau variants. MRI (Longitudinal): Total intracranial volume (TICV), hippocampal volume, and entorhinal cortical thickness. Genomics: Numerical vector forms of gene expression, methylation, and Single Nucleotide Polymorphisms (SNPs).
Zhang & Shi [605]	2020	AD vs. NC (119 AD, 163 NC); SMCI vs. PMCI (153 SMCI, 65 PMCI); 4-Class (119 AD, 105 LMCI, 113 EMCI, 163 NC)	ADNI	MRI, FDG-PET	DMFNet_v2 (attention-based deep network)	Train/Test/Val (60/20/20)	AD vs. NC: 95.21% Acc. (93.56% sens., 97.48% spec.); SMCI vs. PMCI: 89.79% Acc. (81.15% sens., 93.46% spec.); 4-Class: 86.15% Acc. (using attention mechanism fusion)	20 most descriptive 2D image slices (resampled to 180x180x180) automatically screened via AlexNet. Features focus on disease-related atrophy in the hippocampus and entorhinal cortex via an attention mechanism that assigns higher weights to branches with valid information.

Continued on next page

Table S2 – *continued from previous page*

**Reference**	**Year**	**Comparison Groups**	**Database**	**Data Type**	**Best Classifier**	**Validation**	**Best Result**	**Features Used**
Shao *et al.* [549]	2020	AD vs. NC (160 AD, 211 NC); LMCI vs. NC (187 LMCI, 211 NC); EMCI vs. LMCI (273 EMCI, 187 LMCI)	ADNI	MRI, FDG-PET	Hypergraph based multi-task feature selection + MK-SVM	10-fold cross-validation, repeated 10 times (9/1 train/test subsets)	AD vs. NC: 92.51% Acc. (94.08% sens., 90.44% spec., AUC 0.95); LMCI vs. NC: 82.53% Acc. (86.09% sens., 78.55% spec., AUC 0.80); EMCI vs. LMCI: 75.48% Acc. (83.84% sens., 63.26% spec., AUC 0.71) (using hypergraph high-order structure modelling)	90 ROIs per modality (180 total, cerebellum removed) based on MarsBaR AAL atlas. Features include normalized Gray Matter (GM) density maps (2x2x2 mm^3 voxels) and FDG-PET glucose utilization measurements, pre-adjusted for age, gender, and education.
Ramzan *et al.* [532]	2019	AD vs. CN vs. SMC vs. EMCI vs. MCI vs. LMCI (25 AD, 25 CN, 25 SMC, 25 EMCI, 13 MCI, 25 LMCI)	ADNI	MRI, rs-fMRI	Off-the-shelf (OTS) ResNet-18	Hold-out validation with a 70/20/10 train/validation/test split ratio	Multiclass Average (OTS): 97.92% Accuracy, 0.9997 AUC; Multiclass FT Model: 97.88% Accuracy, 0.9997 AUC; FT Stage-Specific: CN 100%, AD 98.01%	Automated deep functional features representing patterns of blood oxygenation level-dependent (BOLD) signals in the resting state. These features capture differences in functional connectivity and neural activity between healthy subjects and AD patients. The data is processed as 2D functional images (64x64) converted from 4D scans along the image height and time axis, excluding non-functional slices. Features characterize the pathogenesis and progression across six stages of cognitive decline.

Continued on next page

Table S2 – *continued from previous page*

Reference	Year	Comparison Groups	Database	Data Type	Best Classifier	Validation	Best Result	Features Used
Hao *et al.* [427]	2020	AD vs. NC (51 AD, 52 NC); MCI vs. NC (99 MCI, 52 NC); MCI-C vs. MCI-NC (43 MCI-C, 56 MCI-NC)	ADNI-1	MRI, FDG-PET	MFCC-MK-SVM (Consistent Metric Constraint)	10-fold cross-validation (9/ 1 train/test subsets)	AD vs. NC: 97.60% Acc. (98.43% sens., 96.73% spec., AUC 0.98) (ADNI-1); MCI vs. NC: 84.47% Acc. (94.04% sens., 66.15% spec., AUC 0.81) (ADNI-1); MCI-C vs. MCI-NC: 77.76% Acc. (67.44% sens., 85.54% spec., AUC 0.76) (ADNI-1); LMCI vs. NC: 78.47% Acc. (85.88% sens., 70.16% spec., AUC 0.78) (ADNI-2); EMCI vs. LMCI: 73.87% Acc. (90.55% sens., 49.52% spec., AUC 0.70) (ADNI-2)	ADNI-1: 186 features (93 ROI VBM-MRI volumes + 93 PET average intensities). ADNI-2: 180 features (90 ROIs GM density + 90 PET glucose utilization). Key discriminative ROIs identified: Right Middle Temporal Gyrus, Left Hippocampal Formation, and Precuneus.

Continued on next page

Table S2 – *continued from previous page*

Reference	Year	Comparison Groups	Database	Data Type	Best Classifier	Validation	Best Result	Features Used
Lei *et al.* [478]	2020	NC vs. SMC (44 NC, 44 SMC); NC vs. EMCI (44 NC, 44 EMCI); NC vs. LMCI (44 NC, 38 LMCI); EMCI vs. LMCI (44 EMCI, 38 LMCI)	ADNI	rs-fMRI, DTI (MRI 3.0T)	Low Rank Self-calibrated + Joint Non-Convex Multi-task learning + SVM	Leave-one-out (LOO) cross-validation	NC vs. LMCI: 87.80% Acc. (84.21% sens., 90.91% spec., AUC 0.989); NC vs. SMC: 82.95% Acc. (88.64% sens., 77.27% spec. AUC 0.898); NC vs. EMCI: 85.23% Acc. (85.23% sens., 86.36% spec., AUC 0.841); SMC vs. EMCI: 84.09% Acc. (81.82% sens., 86.36% spec., AUC 0.913); SMC vs. LMCI: 90.24% Acc. (89.47% sens., 90.91% spec., AUC 0.967); EMCI vs. LMCI: 81.71% Acc. (78.95% sens., 84.09% spec., AUC 0.921) (using joint non-convex subspace learning)	4005 elements derived from a 90x90 connectivity matrix (upper triangle). Features represent Functional Brain Networks (FBN) from rs-fMRI and structural connectivity from DTI. The top 50 features with the largest weights were selected for the final classifier.
Kang *et al.* [455]	2020	EMCI vs. NC (70 EMCI, 50 NC)	ADNI	MRI, DTI (FA and MD maps)	VGG16 (Transfer Learning) + LASSO + SVM using multi-modality data	5-fold cross-validation (80/ 20 train/test)	EMCI vs. NC: 94.2% Acc. (97.3% sens., 92.9% spec., AUC 0.953) (fusing FA, MD, and sMRI)	8192-dimension feature vector concatenated from 32 merged RGB slices (256 deep features per slice). Features prioritize the temporal lobe, hippocampus, and callosum. DTI (FA/MD) acted as a critical supplement to sMRI for detecting microstructure changes.

Continued on next page

Table S2 – *continued from previous page*

Reference	Year	Comparison Groups	Database	Data Type	Best Classifier	Validation	Best Result	Features Used
Gupta *et al.* [425]	2020	AD vs. HC (33 AD, 35 HC); MCIs vs. MCIc (30 MCIs, 31 MCIc)	ADNI	MRI, FDG, AV45, DTI (FA), rs-fMRI, APOE genotype	EasyMKL with Combined (VOI + ROI) features	70:30 train-test split; LOOCV for hyper-parameter optimization	AD vs. HC: 98.52% Acc. (AUC 0.978); MCIs vs. MCIc: 95.08% Acc. (AUC 0.969); AD vs. MCIs: 96.68% Acc. (AUC 0.963); HC vs. MCIs: 96.97% Acc. (AUC 0.966); HC vs. MCIc: 96.65% (using Combined VOI+ROI); AD vs. MCIc: 96.20% (using Combined-VOI)	1404 total features. Regional (ROI): 384 AICHA ROIs (sMRI/PET), 200 Craddock ROIs (fMRI), 50 JHU-WM ROIs (DTI), 2 APOE genotypes. Voxel-wise (VOI): GM volumes, glucose rate, REHO, ALFF, and fALFF indices. Significant region: (Left/ Right) Precentral region.
Fang *et al.* [405]	2020	AD vs. MCI vs. NC (93 AD, 204 MCI, 101 NC)	ADNI	MRI (1.5T), FDG-PET	Adaboost ensemble (DCMA method)	65/10/ 25 train/ validation/ test split; 10 repeats	AD vs. NC: 99.27% Acc, 95.89% Sens, 98.72% Spec; AD vs. MCI: 92.57% Acc, 89.71% Sens, 93.59% Spec; MCI vs. NC: 90.35% Acc, 88.36% Sens, 92.56% Spec	Subject-level probabilistic scores from DCNN models. MRI features are extracted from slices indices 92–107 and PET features from indices 35–60, representing whole-brain metabolic and structural information resized to 224x224.
Shojaie *et al.* [554]	2021	AD vs. MCI vs. CN (67 AD, 378 MCI, 277 CN)	ADNI3	Amyloid-PET, Tau-PET, MRI	SVC with MMI-based feature selection	Nested cross-validation (inner 5-fold optimization, outer 6-fold validation)	CN vs. AD: 93.3% Acc, 88.9% Prec, 79.4% Recall, 83.2% F1; MCI vs. AD: 82.4% Acc, 0.718 F1; CN vs. MCI: 75.9% Acc, 0.700 F1; Multiclass: 71.5% Acc, 0.612 F1	Regional SUVR values (Amyloid: frontal pole, accumbens; Tau: entorhinal, amygdala, third ventricle); MRI Structural data (cortical thickness, surface area, cortical volume); AD risk factors: Age, Gender, APOE $\varepsilon 4$ status, and Education.

Continued on next page

Table S2 – *continued from previous page*

Reference	Year	Comparison Groups	Database	Data Type	Best Classifier	Validation	Best Result	Features Used
Palmqvist *et al.* [519]	2021	Converters vs. Non-converters (BioFINDER: 91 AD, 249 stable; ADNI: 102 AD, 441 stable)	Bio-FINDER; ADNI	Plasma biomarkers, cognitive tests, MRI	Parsimonious model (Plasma P-tau, cognition, APOE alleles)	Independent multi-center cohort validation (Bio-FINDER train, ADNI test)	AD Prediction (4-year): 0.92 AUC (BioFINDER Best Model); 0.91 AUC (ADNI Best Model); SCD subgroup: 0.95 AUC; MCI subgroup: 0.86 AUC	Plasma P-tau217/181, APOE $\varepsilon4$ allele count, Memory function (10-word delayed recall z-score), Executive function (Trail-Making Test B z-score), and "AD signature" cortical thickness (temporal AD-specific region).
Venugopalan *et al.* [51]	2021	AD vs. MCI vs. CN (Clinical: 707 AD, 699 MCI, 598 CN; Imaging: 266 AD, 104 MCI, 132 CN)	ADNI	MRI, genetics (SNPs), EHR	SDAE combined with Random Forest (EHR+ Imaging+ SNP)	10% external test set; 10-fold cross-validation on 90% data	Multiclass (CN/ MCI/AD): Imaging+EHR+SNP: 0.88 Acc (CN), 0.80 Acc (MCI), 0.88 Acc (AD); CN vs. AD: 0.86 Acc, 0.86 Prec, 0.87 Recall (Imaging only); CN vs. MCI/AD: 0.89 Acc, 0.9 Prec (SNP only)	1680 EHR features (Memory tests: summary score, RAVLT; medication; metabolic markers); 500 genetic SNPs (on known AD genes); 9108 3D MRI voxels representing 21 extracted brain regions (hippocampus, amygdala, superior temporal, etc.).
Turkson *et al.* [574]	2021	AD vs. MCI vs. NC (150 AD, 150 MCI, 150 NC)	ADNI	MRI (T1 and T2)	Spiking Deep CNN	Three-binary classification (130 train, 20 test per class)	AD vs. NC: 90.15% Acc, 96.5% Sen, 87.1% Spe; AD vs. MCI: 87.3% Acc, 90.2% Sen, 85.3% Spe; NC vs. MCI: 83.9% Acc, 88.9% Sen, 75.6% Spe	MRI (Anatomical): 138 anatomical structure volumes parcellated via AAL atlas; MRI pixel values stacked as multi-dimensional activation maps. Relevant features are those capable of discriminating between AD and cognitively normal groups.

Continued on next page

Table S2 – *continued from previous page*

Reference	Year	Comparison Groups	Database	Data Type	Best Classifier	Validation	Best Result	Features Used
Kamal *et al.* [454]	2021	NC (2560) vs. Very Mild (1792) vs. Mild (717) vs. Moderate Demented (52)	Kaggle, OASIS-3, NCBI (GSE174 367)	MRI, microarray gene expression	CNN (Images); SVC (Genes)	4-class classification (80/20 train/test split)	MRI: 97.6% Acc, 97.8% Prec, 98.4% sens.; Gene: 82.4% Acc, 81.8% Prec	MRI: Binary pixel regions (white pixels) representing Alzheimer's affected brain areas. Genes: 18,234 gene expression levels; most significant identified via LIME include OR8B8, ATP6V1G1, FZD4, and ATP6AP1 for AD, and HTR1F and OR6B2 for non-AD.
El-Sappagh *et al.* [401]	2021	CN (294) vs. MCI (486) vs. AD (268)	ADNI	MRI, PET, genetic, cognitive, clinical	Random Forest	2-Layer hierarchical model (Multi-class L1; Binary L2 sMCI vs. pMCI) (90/10 split)	L1 (AD/MCI/CN): 93.95% Acc (CV), 93.3% Acc (Test); L2 (sMCI/pMCI): 87.08% Acc (CV), 91.86% Acc (Test)	5 Modality Groups (188 features): (1) Cognitive: MMSE, CDRSB, FAQ, ADAS11/13, MOCA; (2) PET: FDG, HCI, SROI; (3) MRI (T1): Volumes of Ventricles, MidTemp, Fusiform, Entorhinal, Hippocampus, ICV, and Whole Brain; (4) Genetic: APOE4; (5) CSF: Tau, pTau, Aβ1–42.

Continued on next page

Table S2 – *continued from previous page*

Reference	Year	Comparison Groups	Database	Data Type	Best Classifier	Validation	Best Result	Features Used
Abdelaziz *et al.* [340]	2021	NC vs. AD (226 NC, 186 AD); NC vs. sMCI (226 NC, 226 sMCI); NC vs. pMCI (226 NC, 167 pMCI)	ADNI	MRI, PET, SNPs (genetics)	Supervised CNN (A unified DL framework for joint classification of stages and regression of clinical scores, designed to handle incomplete Multi-modal data)	K-fold CV (indicated by means/ SD); unified framework using maximum samples	NC vs. AD: 98.22% Acc, 97.78% Sen, 98.76% Spec, 0.973 AUC. NC vs. sMCI: 93.11% Acc, 92.65% Sen, 93.57% Spec, 0.935 AUC. NC vs. pMCI: 97.35% Acc, 97.82% Sen, 96.71% Spec, 0.973 AUC (Results for MRI+PET+SNPs fusion).	Neuroimaging (186 total): GM volumes from 93 MRI ROIs (normalized by intracranial volume) and average intensity values from 93 PET ROIs. Genetic (23,523 total): SNPs annotated for the top 27 risk genes. Clinical Scores (Targets): MMSE, CDR-SOB, CDR-GLOB, and ADAS-Cog. Top Identified ROIs: Superior Frontal Gyrus, Thalamus, Inferior Frontal Gyrus, Hippocampal Formation, and Precuneus.
Zhang *et al.* [606]	2021	AD vs. MCI vs. HC (38 AD, 42 MCI, 40 HC)	ADNI	MRI (T1), PET, CSF	Proposed lp-norm MCSVM (multiclass support vector machine)	5-fold CV (parameter search); 30-trial record average	T1 (AD vs. MCI): 89.63% Acc. T2 (AD vs. HC): 94.58% Acc. T3 (MCI vs. HC): 81.02% Acc. T4 (Multiclass): 88.54% Acc (lp-norm MCSVM with MRI+PET fusion).	Neuroimaging: 90 cerebral ROI volumes extracted from MRI and 90 average intensity values from PET via the AAL template. Biomarkers: CSF protein levels (Aβ42, t-tau, p-tau) used for modality fusion. Clinical Criteria: MMSE scores and Clinical Dementia Rating (CDR).

Continued on next page

Table S2 – *continued from previous page*

Reference	Year	Comparison Groups	Database	Data Type	Best Classifier	Validation	Best Result	Features Used
Wang *et al.* [589]	2021	AD vs. MCI vs. NC (34 AD, 36 MCI, 50 NC)	ADNI	MRI (T1), fMRI	Multi-modal fusion with KCCA + SVM	70% Training / 30% Testing split; repeated 10 times	AD vs. NC: 96% Acc, 100% Sen, 93.3% Spec, 99.3% AUC. AD vs. MCI: 100% Acc, 100% Sen, 100% Spec, 100% AUC. MCI vs. NC: 100% Acc, 100% Sen, 100% Spec, 100% AUC. LMCI vs. EMCI: 100% Acc, 100% Sen, 100% Spec, 100% AUC (Results for KCCA fusion).	Structural (sMRI): 3D Gray Matter features representing neuronal cell distribution and atrophy degrees. Functional (fMRI): Brain Functional Connection Matrix calculated using Pearson correlation coefficients between brain intervals (90 or 116 regions based on AAL template).
Ning *et al.* [513]	2021	AD vs. NC (93 AD, 99 NC – ADNI1; 136 AD, 107 NC – ADNI2); pMCI vs. sMCI (79 pMCI, 121 sMCI – ADNI1; 82 pMCI, 103 sMCI – ADNI2)	ADNI-1 and ADNI-2	MRI (T1), PET	Proposed SRL with Relational Regularizers	10-fold CV	AD vs. NC: 96.9% Acc, 0.976 AUC. pMCI vs. sMCI: 85.9% Acc, 0.856 AUC. MCI vs. NC: 82.6% Acc, 0.820 AUC (Best results across ADNI1/ADNI2).	ROI-based Features: 90 MRI ROI features (GM tissue volume) and 90 PET ROI average intensity values. Top Biomarkers Identified: Hippocampus, Putamen, Insula, and Pallidum (MRI); Orbitofrontal Cortex, Temporal Pole, and Superior Frontal Gyrus (PET).

Continued on next page

Table S2 – *continued from previous page*

Reference	Year	Comparison Groups	Database	Data Type	Best Classifier	Validation	Best Result	Features Used
Yang *et al.* [598]	2022	MCI vs. NC (55 MCI, 64 NC)	Shanghai Pudong cohort	MRI, DTI, ASL, DWI, serum biomarkers	Random Forest (multi-modal)	Train/test	MCI vs. NC: 97.23% Acc, 94.44% Sens, 100% Spec, 0.97 AUC. (Validation: Randomly split training/test sets).	Multi-modal Biomarkers: Medians from 116 brain regions: FA (DTI), ADC (DWI), and CBF (ASL). Serum: Aβ1-40, Aβ1-42, P-tau, glucose, and lipids (TC, TG, LDL, HDL). Key features: FA of left cerebellum_4_6, Age, and Aβ1-40.
Lao *et al.* [474]	2022	AD vs. MCI vs. CN (144 AD, 203 MCI, 181 CN)	ADNI-2	MRI (3.0T), FDG-PET	MP-SAE (Multi-modal SAE)	5-fold cross-validation	AD vs. CN: 96.92% Acc, 100% Spec, 0.985 AUC. pMCI vs. sMCI: 87.78% Acc, 0.847 AUC. CN vs. pMCI: 90.21% Acc, 0.943 AUC. AD vs. sMCI: 89.09% Acc, 0.93 AUC.	Multi-modal Shape & Texture: 90 ROIs from MRI (GM) and PET. 3D-Discrete Wavelet Transform (approximate components LLL subband). 5 Moment Invariants (3D-MIs) representing total spatial variance (J1), spatial covariance (J2, J3), skewness (B3), and kurtosis (B4).
Golovanevsky *et al.* [421]	2022	AD vs. MCI vs. CN (35 AD, 39 MCI, 165 CN)	ADNI (1, 2, GO)	MRI, genetic (SNPs), clinical data	MADDi (Cross-modal attention)	Train/Test (90/10), 3-fold cross-validation	3-class (CN/MCI/AD): 96.88% Acc (held-out test set), 91.41% F1-score. AD individual class: 100% Precision, 100% Acc.; CN individual class: 96.66 Acc.; MCI individual class: 96.66% Acc.	Multi-modal inputs: Imaging: 3 center slices (72x72) from T1 MRI. Genetic: 15,000 SNPs (selected from 3M). Clinical (29 features): Memory, executive function, and language scores; balance tests; and demographics.

Continued on next page

Table S2 – *continued from previous page*

Reference	Year	Comparison Groups	Database	Data Type	Best Classifier	Validation	Best Result	Features Used
Jia & Lao [450]	2022	NC vs. SMC vs. MCI vs. AD (50 NC, 26 SMC, 35 MCI, 34 AD)	ADNI	fMRI (mReHo), MRI (T1)	SVM with KCCA-fused features	70% Train / 30% Test split; 10 experimental replicates	MCI vs. AD: Acc. 95.00%, AUC 0.950, Sens. 90.0%, Spec. 100.0%. NC vs. SMC: Acc. 91.30%, AUC 0.875. NC vs. MCI: Acc. 92.00%, AUC 0.917. SMC vs. MCI: Acc. 94.44%, AUC 0.938.	Spatiotemporal Texture Features: fMRI Mean Regional Homogeneity (mReHo) images reflecting local voxel similarity via Kendall's coefficient; and sMRI texture/nonlinear features learned via residual mapping focusing on brain texture changes.
Dwivedi *et al.* [398]	2022	CN vs. MCI vs. AD (100 CN, 100 MCI, 100 AD)	ADNI	MRI (T1), FDG-PET	ResNet-50 + RELS-TSVM	10-fold cross-validation; 90% Train / 10% Test split	AD vs. MCI: Acc. 97,5%, Spec. 99%, Sens. 96%. CN vs. AD: Acc. 97%, Spec. 97%, Sens. 97%. CN vs. MCI: Acc. 94%, Spec. 91%, Sens. 97%.	Multi-modal Fused Features: Integrated anatomical (MRI) and metabolic (PET) deep features automatically generated from fused imagery. Fused data utilizes Daubechies 1 level 2 wavelet coefficients to capture both frequency changes and tissue detail.

Continued on next page

Table S2 – *continued from previous page*

Reference	Year	Comparison Groups	Database	Data Type	Best Classifier	Validation	Best Result	Features Used
Meng *et al.* [503]	2022	HC vs. EMCI vs. AD (33 HC, 29 EMCI, 23 AD)	ADNI	rs-fMRI, DTI	Multi-modal LassoNet	10 trials; 6:2:2 Train/ Validation/ Test split	AD-HC: Acc. 90.68%, SEN 88.81%, SPE 91.91%, F1 88,25%, AUC 0,912. EMCI-HC: Acc. 88,77%, AUC 0.8975. AD-EMCI: Acc. 83.63%, AUC 0.8478.	Fused Connectivity Features: rs-fMRI BOLD signal time series from 90 ROIs (AAL template); and DTI structural network penalty matrices based on Fractional Anisotropy (FA) values between ROIs. Focuses on coupling strength between functional and structural connectivity.
Wang *et al.* [590]	2023	HC vs. EMCI vs. LMCI vs. AD (24 HC, 24 EMCI, 24 LMCI, 24 AD)	ADNI	rs-fMRI (T1/T2 structure data)	MRMR feature selection + Linear SVM	Nested 5-fold cross-validation (80/20 split per fold)	HC vs. AD: Acc. 96.80%. HC vs. LMCI: Acc. 92.90%. EMCI vs. AD: Acc. 89.50%. HC vs. EMCI: Acc. 85.60%. LMCI vs. AD: Acc. 84.90%. EMCI vs. LMCI: Acc. 83.30%.	2166 Graph Measures: 6 global and 360 local metrics per node. Global: Assortativity, Globe Efficiency, Small World, Hierarchy, Synchronization, and Characteristic Path Length. Local: Clustering Coefficient, Local Efficiency, Betweenness Centrality, Eigenvector Centrality, Nodal Degree, and Shortest Path.
Long *et al.* [494]	2023	AD vs. MCI vs. HC (44 AD, 66 MCI, 58 HC)	Local Clinic (Nanfang Hospital)	rs-fMRI, MRI (MP-RAGE)	SVM (RBF kernel)	Nested 10-fold cross-validation	Multi: 80.36% Acc. (AD&MCI) vs. HC: 88.69% Acc, 0.91 AUC. AD vs. MCI: 86.14% Acc, 0.91 AUC.	AAL3 atlas metrics: Gray Matter Volume (GMV) from sMRI; Hurst Exponent (HE) from fMRI; and bilateral hippocampus seed-based independent connectivity mappings.

Continued on next page

Table S2 – *continued from previous page*

Reference	Year	Comparison Groups	Database	Data Type	Best Classifier	Validation	Best Result	Features Used
Wang *et al.* [591]	2023	AD vs. HC/NC (118 AD, 127 NC)	ADNI	rs-fMRI (3T Philips)	Combined PSI + 2D-CNN	5-fold cross-validation (4:1 split)	Acc: 98.869%, Sens: 99.124%, Spec: 98.614%, AUC: 0.998	Hybrid Brain Network features: 8 topological metrics (Degree, Node/Edge Betweenness, Assortativity, Clustering Coefficient, Global/Local Efficiency, Path Length) and 8 deep texture/object pattern features from 2D-CNN convolutional layers.
Hassouneh *et al.* [428]	2024	MCIs (Stable) vs. MCIc (Conversion) (137 participants)	ADNI	MRI (T1, coronal), PET	Probabilistic Neural Network (PNN)	Not specified	MCIc vs. MCIs: Acc: 92.89%, Prec: 93.5%, Sens: 93.2%, Spec: 93.2%, F1: 92.9%, AUC: 94%	Fused Multi-modal metrics: Coronal T1 ROI volumes (Hippocampus/Entorhinal), 2D-GLCM texture (Contrast, Dissimilarity, Homogeneity, Correlation, ASM), and PET SUVR measuring Amyloid-beta and Tau protein accumulation.
Jitsuishi *et al.* [453]	2022	EMCI vs. LMCI (34 EMCI, 32 LMCI)	ADNI-3	MRI (T1, DWI, rs-fMRI)	AdaBoost	Binary (EMCI vs. LMCI); Validation: Stratified 10-fold CV	EMCI vs. LMCI (Region subset #7): 71% Accuracy; 0.73 AUC; 75% Recall; 77% Precision; 0.70 F1-score. MD-based Subset: 70% Acc; 79% AUC.	Multi-modal Markers: (1) Diffusion: FA and MD values in WM regions (Corpus Callosum, cingulum, insula, thalamus). (2) Functional: RSFC coefficients and graph metrics (clustering coefficient, local efficiency) in cortical areas (frontal 10d/9a, precentral 6mp/SFL, parietal 5L/7AL).

Continued on next page

Table S2 – *continued from previous page*

Reference	Year	Comparison Groups	Database	Data Type	Best Classifier	Validation	Best Result	Features Used
Jin *et al.* [452]	2022	EMCI vs. LMCI (151 EMCI, 209 LMCI total after synthesis)	ADNI	MRI, FDG-PET	Proposed Hybrid Method (FF-GAN + fine-tune)	Binary (EMCI vs. LMCI); Validation: 10-fold CV (paired); 5-fold CV (complete with synthetic data)	EMCI vs. LMCI (Synthetic PET): 84.47% Accuracy; 0.9281 AUC; 85.53% Sens; 81.48% Spec. Validation Set (Real PET): 85.21% Accuracy; 0.9364 AUC.	Fused 3D Image Features: Integrated information from MRI and PET scans. Dominant ROI Clusters: Features from the hippocampus and temporal lobe (MRI-derived) and the cingulate gyrus and precuneus (PET-derived) were most discriminative.
Dong *et al.* [396]	2022	AD vs. MCI vs. NC (52 AD, 52 MCI, 52 NC)	ADNI	MRI (T1), FDG-PET	Latent feature fusion + SVM	10-fold CV; internal 5-fold CV for parameters	AD vs. NC: Acc. 90.74%, Sens. 90.14%, Spec. 88.16%, AUC 96.36%. MCI vs. NC: Acc. 74.73%, Sens. 73.62%, Spec. 74.48%, AUC 77.47%. AD vs. MCI: Acc. 75.54%, Sens. 77.52%, Spec. 78.54%, AUC 85.47%	90 ROIs (AAL template): Average volume values for MRI and average intensity values for PET. Key discriminative regions include the hippocampus, amygdala, insula, and temporal lobe (MRI), and the precuneus and gyri (PET).

Continued on next page

Table S2 – *continued from previous page*

Reference	Year	Comparison Groups	Database	Data Type	Best Classifier	Validation	Best Result	Features Used
Oliveira *et al.* [515]	2024	AD vs. MCI vs. CN (102 AD, 235 MCI, 167 CN)	ADNI 1	MRI (T1 and T2 sequences)	LinSVC or OvsR (with Feature Selection)	Hold-out validation	AD vs. CN: 85.2% Acc, AD vs. MCI: 98.5% Acc, CN vs. MCI: 95.1% Acc, Multi-class: 87.1% Acc	22 GLCM Texture features: Autocorrelation, Contrast, Correlation (1 & 2), Cluster Prominence, Cluster Shade, Dissimilarity, Energy, Entropy, Homogeneity (1 & 2), Maximum Probability, Variance, Sum Average, Sum Variance, Sum Entropy, Difference Variance, Difference Entropy, IMC (1 & 2), IDN, and IDMN.
Alireza Jomeiri *et al.* [613]	2025	AD vs. MCI vs. NC (255 AD, 521 MCI, 376 NC)	ADNI	MRI (1.5T and 3T), PET	Regional Attention-Enhanced Vision Transformer (RAE-ViT)	70/15/15 split (Train/Val/Test); 5-fold cross-validation	Multi-modal (sMRI+PET): 95.8% Acc., 0.97 AUC; Multiclass (sMRI single split): 94.2% Acc., 91.8% Sens., 95.7% Spec., 0.96 AUC; Binary NC vs. AD: 0.96 AUC; NC vs. MCI: 0.91 AUC.	Anatomical Priors: Regional Attention Module (RAM) prioritizes disease-critical ROIs: hippocampus subfields, lateral ventricles, and cortical regions. Global-Local Patterns: Hierarchical self-attention capturing fine-grained patterns within ROIs and long-range structural dependencies across the brain. Multi-resolution: Features extracted from overlapping multi-scale patches (small, medium, large).

Continued on next page

Table S2 – *continued from previous page*

Reference	Year	Comparison Groups	Database	Data Type	Best Classifier	Validation	Best Result	Features Used
Shakhnoza Muksimova *et al.* [509]	2025	Normal vs. MCI vs. AD (Dataset compiled from multiple public sources)	ADNI, OASIS	MRI, PET, CT and longitudinal data	FusionNet	70/15/15 train/validation/test split; k-fold cross-validation	AD vs. MCI vs. Normal: 94% Accuracy, 92% Precision, 93% Recall, 92.5% F1-score.	Modality-Specific: Structural hierarchies (MRI), metabolic activity and amyloid deposition patterns (PET), and high-density structures/atrophy (CT). Longitudinal Dynamics: LSTM-extracted temporal features analyzing subtle changes between sequential time-point scans. Integrated Vectors: Unified 512-dimensional feature representations resulting from Multi-modal vector concatenation.
Vlontzou *et al.* [581]	2025	1463 total: Healthy Controls (449 CN), MCI (740), AD (274)	ADNI	MRI, genetic (SNPs)	SVM (Highest mean balanced accuracy)	5x4 fold nested cross-validation; 80:20 initial Train-Test Split; Multiclass classification via One-versus-One (OVO) decomposition	Multiclass: 87.5% Balanced Accuracy and 90.8% F1-score (Mean results); 89.6% Balanced Accuracy and 92.6% F1 (Max)	MRI-Genetic Integrated Features: 1) 154 MRI Volumetric ROIs: Specifically temporal lobes, hippocampus, amygdala, and lateral ventricles; 2) 54 AD-related SNPs: Including rs429358 (APOE), rs6448453 (CLNK), and rs2081545 (MS4A6A); 3) Demographics: Age, sex, marital status.

Continued on next page

Table S2 – *continued from previous page*

Reference	Year	Comparison Groups	Database	Data Type	Best Classifier	Validation	Best Result	Features Used
Nallapu *et al.* [511]	2024	CN-s (534) vs. AD (131); MCI-s (541) vs. MCI-p (193). Total 2013 participants	ADNI	Plasma, CSF, MRI (HVa)	RF (DA+P+C+H combo)	5-fold internal cross-validation; validated on independent MCI sample	CN-s vs. AD: AUC 0.89. MCI-to-AD Conversion (24 mo): AUC 0.76, 76.1% Sensitivity, 75.0% Specificity. MCI-to-AD (MRI only): AUC 0.70. MCI-to-AD (Plasma only): AUC 0.64.	Multi-modal ATN Features: 1) MRI: Adjusted Hippocampal Volume (HVa) normalized by intracranial volume; 2) Plasma: p-tau181, NfL (Neurofilament light chain); 3) CSF: Aβ42, tau, p-tau181; 4) Genetic/Demographic: Age, sex, education, and number of APOE ε4 alleles.
Aland *et al.* [351]	2025	AD vs. Non-AD (91 "Tumor"/AD-affected, 154 Healthy aging)	ADNI (Training)	MRI, fMRI, EEG	CNN	Hold-out (Training vs. Validation loss)	92.4% Accuracy; 92.1% F1 Score	Multi-modal AI features: MRI (sMRI): Hippocampal volume, cortical thickness, and white matter lesions via image segmentation. EEG: Power spectral density (PSD), entropy, and coherence. fMRI: Activation maps, voxel intensities, and region connectivity. The best binary result utilised the CNN-based MRI classification.
**Studies with fluid biomarkers, cognitive, and language data**								

Continued on next page

Table S2 – *continued from previous page*

Reference	Year	Comparison Groups	Database	Data Type	Best Classifier	Validation	Best Result	Features Used
Aljović *et al.* [356]	2016	AD vs. Healthy (45 AD, 35 Healthy)	Alz-biomarkers database	CSF biomarkers	FF Neural Network (10 hidden neurons)	60 estimation / 20 testing split	AD vs. Healthy: 95.5% Sensitivity, 91.43% Specificity	CSF Biomarkers: 5 biochemical parameters: albumin ratio (CSF/ Serum and/or Plasma), Aβ40, Aβ42, tau-total, and tau-phospho.
Asgari *et al.* [363]	2017	MCI vs. Intact (14 MCI, 27 Intact)	Randomized clinical trial (NCT0157 1427)	Spontaneous spoken utterances	Linear SVM with L1-norm regularization	5-Fold-CV	MCI vs. Intact: Acc 84%, AUC-ROC 79.61% (Sens 80.77%, Spec 75.83%)	Total features: 68-dimensional linguistic vector categorized via LIWC. Sub-categories: (1) Linguistic Dimensions (e.g., pronouns, negations); (2) Psychological Processes (e.g., affective, social, cognitive); (3) Relativity (Time, Space, Motion); (4) Personal Concerns (Occupation, Leisure); (5) Spoken Categories (Fillers, Nonfluencies). Best Subset (10): "Relativity" category (Time/verb tense, Space, Motion).

Continued on next page

Table S2 – *continued from previous page*

Reference	Year	Comparison Groups	Database	Data Type	Best Classifier	Validation	Best Result	Features Used
Hernandez-Dominguez *et al.* [431]	2018	AD vs. MCI vs. HC (257 AD, 43 MCI, 217 HC)	Pitt Corpus (Dementia-Bank)	Audio and text (Cookie Theft task)	SVM	10-fold cross-validation	HC vs. AD: 0.93 AUC (Best model Acc: 94%, RFC Acc: 85%). HC vs. Cognitively Impaired (AD+MCI): 0.87 AUC	105 Multi-modal Features: Coverage: Informativeness and Pertinence (at 60%, 80%, 100% thresholds). Linguistic: vocabulary richness (Brunet's W, Honoré's R, Sichel's S, Yule's K), Type-token ratio (TTR), POS ratios (adjectives, nouns, verbs), and general/average sentence entropy. Phonetic: Mean, kurtosis, skewness, and variance of the first 13 Mel-Frequency Cepstral Coefficients (MFCCs).
Fritsch *et al.* [411]	2019	AD vs. HC (168 AD providing 255 transcriptions, 98 HC providing 244 transcriptions)	Dementia-Bank's Pitt Corpus	Transliterations of "Cookie Theft" picture descriptions (oral/ written language)	NNLM with Long Short-Term Memory (LSTM) cells	Leave-one-speaker-out cross-validation	AD vs. HC: 85.6% Acc. (at EER), 86% Sen. (TPR), 85% Spe. (1-FPR), 0.92 AUC; AD vs. HC (MMSE 21-30): 79.5% Acc., 0.87 AUC	Language Structures: Within-speaker perplexity values evaluated on Alzheimer and control LMs. Features include spontaneous speech patterns, fillers (uhm, uhh), repetitions, paraphrases, and grammatical mistakes captured during the picture description task.

Continued on next page

Table S2 – *continued from previous page*

Reference	Year	Comparison Groups	Database	Data Type	Best Classifier	Validation	Best Result	Features Used
Palmqvist *et al.* [520]	2024	Subjective cognitive decline (279) vs. MCI (535) vs. Dementia (399). Total 1213 patients	Bio-FINDER-1/2 & Bio-FINDER-Primary Care	Blood (Plasma)	APS2 (Amyloid Probability Score 2)	Predefined cutoff values established in independent cohort; single-batch vs. prospective biweekly batch validation	AD pathology vs. No pathology: AUC 0.97 (Primary Care), AUC 0.97 (Secondary Care). Clinical AD Prediction: 91% Diagnostic Accuracy. Subjective Cognitive Decline (SCD): NPV 91%–94% for ruling out AD.	Blood-Based Proteomic Features: The APS2 score integrates: 1) Percentage of plasma phosphorylated tau 217 (p-tau217) relative to non-p-tau217; 2) Plasma Aβ42:Aβ40 ratio. These mitigate effects of comorbidities like kidney disease.
Pan *et al.* [522]	2023	CN vs. SCD vs. MCI vs. AD vs. non-AD (238 CN, 118 SCD, 135 MCI, 89 AD, 29 non-AD)	Chinese Cohort & ADNI	Plasma biomarkers, APOE, cognitive tests	Full Model	1000 times cross-validation	Amyloid Positivity: AUC 0,94. MCI (Full): AUC 0,97 (Acc. 91,9%). AD vs. CN (Plasma): AUC 0,93. AD vs. SCD (Plasma): AUC 0,91. AD vs. MCI (Plasma): AUC 0,92. SCD vs. MCI (Plasma): AUC 0,75.	Multi-modal Indicators: 5 plasma biomarkers (Aβ42, Aβ40, T-tau, P-tau181, NfL); APOE genotypes (e2/e3/e4 risk groups); Cognitive scores (MMSE, MoCA-BC, ACE-III-CV, AVLT recall/recognition, BNT, AFT, STT-A/B, ADL, FAQ); and demographics (age, education, BMI).
**Studies with EEG**								

Continued on next page

Table S2 – *continued from previous page*

Reference	Year	Comparison Groups	Database	Data Type	Best Classifier	Validation	Best Result	Features Used
Ieracitano *et al.* [442]	2020	AD vs. MCI vs. HC (63 AD, 63 MCI, 63 HC)	IRCCS Centro Neurolesi Bonino-Pulejo (Messina, Italy)	EEG (19-channel scalp)	MLP (specifically MLP3 for Multi-modal features)	10-fold Cross-Validation (70% training / 30% testing split per fold)	AD vs. HC: 96.95% ± 0.5% Accuracy; AD vs. MCI: 90.24% ± 0.7% Accuracy; MCI vs. HC: 96.24% ± 0.5% Accuracy; AD vs. MCI vs. HC: 89.22% ± 0.7% Accuracy	Multi-modal (CWT+BiS) features: CWT features (mean, SD, skewness, kurtosis, entropy) and 6 BiS features (including normalized bispectral entropy and spectral moments) extracted from δ, θ, $\alpha1$, $\alpha2$, and β rhythms.
Ieracitano *et al.* [441]	2019	AD vs. MCI vs. HC (63 AD, 63 MCI, 63 HC)	IRCCS Centro Neurolesi Bonino-Pulejo (Messina, Italy)	EEG (19-channel scalp)	CNN1	Epoch-based 70/30 Train/ Test split (no explicit k-fold CV)	Epoch-based: AD vs. HC: 92.95% Accuracy; MCI vs. HC: 91.88% Accuracy; AD vs. MCI: 84.62% Accuracy; AD vs. MCI vs. HC: 83.33% Accuracy. Patient-based (AD vs. HC): 23/24 AD and 15/24 HC correctly identified.	2D Spectral Representations (PSD-images): Gray-scale images (19x159) representing power spectral density across 19 channels and frequency bins (0.5–32 Hz).
Miltiadous *et al.* [504]	2023	AD vs. FTD vs. CN (36 AD, 23 FTD, 29 CN)	AHEPA General University Hospital of Thessaloniki (Greece)	EEG (19-channel scalp)	DICE-net (Dual-Input Convolution Encoder Network)	Leave-One-Subject-Out (LOSO) Validation	AD vs. CN: 83.28% Accuracy; FTD vs. CN: 74.96% Accuracy (Leave-One-Subject-Out validation)	Relative Band Power (RBP) & Spectral Coherence Connectivity (SCC): Image-like 3D matrix representations of power and synchronization across 5 frequency bands (delta to gamma) and 19 channels.

Continued on next page

Table S2 – *continued from previous page*

Reference	Year	Comparison Groups	Database	Data Type	Best Classifier	Validation	Best Result	Features Used
Morabito *et al.* [508]	2016	AD vs. MCI vs. HC (Balanced subset: 23 AD, 23 MCI, 23 HC)	IRCCS Centro Neurolesi "Bonino-Pulejo" (Messina, Italy)	EEG (19-channel scalp)	MC-DCNN	k-fold Cross-Validation (k=4)	AD vs. HC: 85% Accuracy; MCI vs. HC: 85% Accuracy; AD vs. MCI vs. HC: 82% Accuracy (k-fold validation, k=4)	TFR Statistical Parameters: Mean (μ), standard deviation (σ), and skewness (ν) of the energy distributions extracted from CWT maps for each channel across 3 sub-bands (228 total input parameters).
Kang *et al.* [456]	2025	AD vs. FTD vs. CN (35 AD, 22 FTD, 27 CN)	AHEPA General University Hospital (OpenNeuro ID: ds006036 - Eyes-Open)	EEG (19-channel scalp)	DMD + 3D CNN	Leave-N-Subjects-Out (LNSO) Validation (5 batches of 5-fold Subject-wise CV)	FTD vs. CN: 77.06% Accuracy; AD vs. CN: 74.23% Accuracy (Leave-N-Subjects-Out validation)	DMD-based Mode Maps: Spatio-temporal-spectral representations mapped into 50x50x12 3D images, emphasizing coherent dynamics in the 4–40 Hz range.
Stefanou *et al.* [560]	2025	AD vs. FTD vs. CN (36 AD, 23 FTD, 29 CN)	AHEPA General University Hospital (OpenNeuro ID: ds004504 - Eyes-Closed)	EEG (19-channel scalp)	FFT-Spectrogram based CNN	Leave-N-Subjects-Out (LNSO) Validation (5 batches of Subject-level CV)	AD+FTD vs. CN: 80.69% Accuracy; AD vs. CN: 79.45% Accuracy; FTD vs. CN: 72.85% Accuracy (Leave-N-Subjects-Out validation)	FFT Spectrogram Images: RGB image representations (resized to 150x150) of frequency spectrum across 19 channels in the 4–40 Hz range.

Continued on next page

Table S2 – *continued from previous page*

Reference	Year	Comparison Groups	Database	Data Type	Best Classifier	Validation	Best Result	Features Used
Akbar *et al.* [350]	2025	AD vs. FTD vs. CN; AD vs. MCI vs. HC	OpenNeuro (ds004504, ds006036) and OSF Dataset	EEG (18 to 21-channel scalp)	NeuroFusion-Net	Stratified 5-fold Subject-level Cross-Validation (70% training, 15% validation, 15% testing subjects)	OpenNeuro ds004504: 94.27% Accuracy, 0.94 F1-score; OpenNeuro ds006036: 92.15% Accuracy; OSF: 89.5% Accuracy (5-fold stratified subject-level CV; 70/15/15 split)	Hybrid Handcrafted & Deep Features: Log bandpower (delta to gamma), wavelet coefficients (db4), statistical descriptors (mean, SD, skewness, kurtosis), and Permutation Entropy fused with 1D-CNN temporal embeddings.
Alessandrini *et al.* [353]	2022	AD vs. CN (20 AD, 15 Normal)	Clinica Neurologica, Università Politecnica delle Marche (Italy)	EEG (16-channel scalp)	RPCA + RNN	Subject-wise split (30 training subjects, 5 testing subjects); training data further split 75/25 Train/Validation	Original data with PCA: 97.9% Accuracy; Corrupted data (20% erasures) with RPCA filtering: ∼95% Accuracy	Principal Components: 50 PCs (when using PCA reduction) or filtered components (via RPCA) extracted from original 16 EEG signals.
Aljalal *et al.* [355]	2024	MCI vs. HC (29 MCI, 32 HC)	Noor Hospital (Isfahan, Iran) Public Dataset	EEG (resting-state, 19 channels)	Ensemble KNN (ENKNN) with NSGA-II selection	10-fold Cross-Validation	MCI vs. HC: Accuracy 99.81% (10-fold CV)	VMD-Based Entropy Biomarkers: 4 entropy values extracted per channel segment (from VMF1/Beta, VMF2/Alpha, VMF3/Theta, and residual signal). Entropy measures include: Log energy, Shannon, Sure, Threshold (α=0.2), and Norm (p=1.1) entropy. Feature vector length is 4×ch.

Continued on next page

Table S2 – *continued from previous page*

Reference	Year	Comparison Groups	Database	Data Type	Best Classifier	Validation	Best Result	Features Used
Deng *et al.* [393]	2024	HC vs. MCI (84 HC, 53 MCI)	Wayne State Univ. and MADRC (Black Americans)	EEG (resting-state, 64 channels)	Soft Discrimination Model (Weighted Majority Voting)	LOOCV; 10-fold, 5-fold, 3-fold CV (randomly shuffled 10x)	HC vs. MCI: Accuracy 91.97% (LOOCV), 92.55% (10-fold CV)	Multiscale Dynamic Functional Connectivity: Time-varying Pearson correlation coefficients between 12 ROIs (66 region pairs). Primitive Features: Mean, minimum, and maximum of continuous wavelet coefficients (Cj,k) derived from correlation vectors. Final feature vectors formulated from M (10–45) features with smallest P-values via t-test.
Arpaia *et al.* [362]	2025	AD vs. HS (52 AD, 52 HS)	CAUEEG and TUH (Combined hospital datasets)	EEG (rsEEG, 19 channels, eyes closed)	Random Forest (RF) with Global Normalization	LOSO Cross-Validation (74 subjects training / 30 subjects testing)	AD vs. HS: Accuracy 84% ± 10%; MCC = 0.70	Multiscale Fuzzy Entropy (MFE): Calculated independently per channel using scale factors 1–20 (exponential fuzzy membership function, m=2,n=2,r=0.2×SD). Input Features: Mean MFE values for long-scale factors (scales 17–20) calculated for each of the 19 channels, resulting in 19 features per epoch.

Continued on next page

Table S2 – *continued from previous page*

Reference	Year	Comparison Groups	Database	Data Type	Best Classifier	Validation	Best Result	Features Used
Kim *et al.* [466]	2025	SCD vs. MCI vs. AD (20 SCD, 28 MCI, 10 AD)	Korea University Anam Hospital	EEG (resting-state vs. task-based SQT, 19 channels)	Attention-LSTM (Task-based SQT)	5-fold Cross-Validation (80/20 Train-Validation/ Test split)	SCD vs. MCI: Acc 92.00%, AUC 0.97 (REST); MCI vs. AD: Acc 88.75%, AUC 0.97 (SQT); 3-Class: Acc 79.16%, AUCs 0.92/ 0.97/0.95 (SQT)	Multi-modal Raw & Behavioral Features: (1) Raw EEG: Temporal sequences (768 samples $\times$ 19 channels) capturing dynamic changes and spatial relationships. (2) SQT Behavioral Data: Accuracy (Acc), Reaction Time (RT), and standard deviation of reaction time (stdRT) across 10 trials.
Senkaya *et al.* [546]	2025	AD vs. FTD vs. HC (36 AD, 23 FTD, 29 HC)	Thessaloniki AHEPA General Hospital	EEG (rsEEG, 19 channels, eyes closed)	SVM (Quadratic kernel)	Train/Test split (70% training / 30% testing)	AD vs. FTD vs. HC: Accuracy 96.01%, Sensitivity 0.97, Specificity 0.95, AUC 0.98	342 Statistical & Spectral Features: (1) Time domain (7/ch): Kurtosis, Average, RMS, Skewness, SD, Variance, Norm. (2) Spectral domain (11/ ch): Band powers (Delta-Gamma) and 6 power ratios (DTBPR, DABPR, DBBPR, TABPR, TBBPR, ABBPR). Selected Features: 130 informative features (primarily spectral) ranked by Spearman correlation.

Continued on next page

Table S2 – *continued from previous page*

Reference	Year	Comparison Groups	Database	Data Type	Best Classifier	Validation	Best Result	Features Used
Maturana-Candelas *et al.* [500]	2019	AD continuum (51 HC, 51 MCI, 51 ADMIL, 50 ADMOD, 50 ADSEV)	Porto University	EEG (rsEEG, 19 channels, eyes closed)	Quadratic Discriminant Analysis (QDA)	Leave-one-out (LOO) Cross-Validation	Controls vs. AD: Accuracy 79.1% (no MCI); AD vs. Non-AD: Accuracy 69.7% (MCI+HC)	62 Complexity & Irregularity Features: Multiscale Sample Entropy (MSE) and Refined Multiscale Spectral Entropy (rMSSE) computed for 25 scales. Features include: (1) Mean entropy at each scale; (2) Global parameters: Averaged slope values and Areas Under the Curve (AUC) for MSE and rMSSE profiles across whole range, low scales, and high scales.
Trinh *et al.* [572]	2021	AD vs. MCI vs. HC (23 AD, 24 MCI, 27 HC)	Taipei Veterans General Hospital	EEG (rsEEG, 30 electrodes, before/ after memory task)	SVM (Between-run similarity)	Leave-one-participant-out cross-validation (LOPO-CV)	MCI vs. HC: Accuracy 80.39%; AD vs. HC: Accuracy 78.00%; AD vs. MCI: Accuracy 74.47%	Between-Run Similarity (BRS): Euclidean distance between spectral power vectors of Run 1 and Run 2. Spectral info: Powers for Delta, Theta, Low/High Alpha, Low/High Beta, and Gamma bands. Spatial features: Average BRS calculated for 6 scalp regions: Frontal, Central, Parietal, Occipital, Left Temporal, and Right Temporal. BRS values are standardized using HC as benchmark.

Continued on next page

Table S2 – *continued from previous page*

Reference	Year	Comparison Groups	Database	Data Type	Best Classifier	Validation	Best Result	Features Used
Deng *et al.* [394]	2025	AD vs. MCI vs. NC (101 AD, 82 MCI, 57 NC)	CMU Hospital (Chongqing, China)	EEG (rs-EEG + passive auditory ERP, 20 channels)	Ensemble Learning (Integrated rs-EEG/ERP/ Clinical)	5-fold Cross-Validation	NC vs. AD: Acc 90.22%, AUC 0.90 (rsEEG/ERP); NC vs. MCI: Acc 85.40% (rsEEG/ERP); MCI vs. AD: Acc 85.95% (Hybrid); 3-way: Acc 80.36%, AUC 0.94 (Hybrid)	Integrated Multi-modal Features: (1) rs-EEG (684): Abs/Rel power (Delta-Gamma); Hjorth parameters; microstate metrics; functional connectivity (PLV, PLI, wPLI, COH) averaged pairwise. (2) Auditory ERP (120): Peak amplitude and latency for P50 (40–70ms), N100 (80–140ms), and MMN (100–250ms) across all 20 channels. (3) Clinical/ Neuropsych: MMSE and TMT scores.

Continued on next page

Table S2 – *continued from previous page*

Reference	Year	Comparison Groups	Database	Data Type	Best Classifier	Validation	Best Result	Features Used
Kim *et al.* [465]	2024	SCD vs. naMCI vs. aMCI vs. AD (20 SCD, 10 naMCI, 18 aMCI, 10 AD)	Korea University Anam Hospital	EEG (rsEEG vs. Memory-Encoding, 64 channels)	cKNN (Memory-encoding state)	Leave-one-subject-out (LOSO) strategy	4-class (SCD/naMCI/aMCI/AD): Accuracy 93.10%; AUCs: 0.96 (SCD), 0.89 (naMCI), 0.95 (aMCI), 1.00 (AD)	Brain Wave Characteristic Features: (1) Phase Activity: Total, phase-locked, and non-phase-locked activity derived via time-frequency decomposition. (2) Spectral: AP and RP in Delta, Theta, Alpha, Beta1, Beta2, and Gamma bands. (3) Temporal: Data divided into 400ms intervals (-400 to 2000ms) with interval difference features. Extracted across 7 brain regions (FM, LT, RT, CM, LP, RP, PM). Total features: 2772 per encoding session.
Rodrigues *et al.* [535]	2016	AD vs. MCI vs. Control (18 AD [10 mild/mod, 8 advanced], 8 MCI, 11 Controls)	Hospital de São João, Porto	EEG (19 channels)	Surrogate decision tree	LOOCV and 10-fold CV	94.88% Accuracy (86.19% sens., 99.35% spec.) with 10 features	Temporal/Spectral: Maxima (NMax), minima (NMin), zero-crossing rate (Zcr), and mean derivative at a point (Mdif). Relative Power (RP): δ (1-4Hz), θ (4-8Hz), α (8-13Hz), $\beta1$ (13-19Hz), $\beta2$ (19-30Hz), γ (30-40Hz). Spectral Ratios (r1-r4): r1 (θ/α), r2 ($\delta+\theta/\alpha+\beta1+\beta2$), r3 ($\beta1+\beta2/\delta$), r4 ($\beta2/\delta$).

Continued on next page

Table S2 – *continued from previous page*

Reference	Year	Comparison Groups	Database	Data Type	Best Classifier	Validation	Best Result	Features Used
Araújo *et al.* [361]	2022	C vs. MCI vs. ADM vs. ADA (11 C, 8 MCI, 11 ADM, 8 ADA)	Hospital de São João, Porto	EEG (19 channels)	SVM / DT (binary); Med. Gaussian SVM (all-vs-all)	LOOCV	93.8% Accuracy (MCI vs. ADA) on O1 electrode; 88.9% (MCI vs. ADM) on P7	non-linear (10 features): Energy, Shannon Entropy, Logarithmic Entropy, Approximate Entropy, Sample Entropy, Permutation Entropy, Norm Entropy, Hurst Exponent, Lyapunov Exponent, Higuchi Exponent. Statistical (10 features): Max, Mean, Median, Mode, Kurtosis, SD, Variance, Skewness, RMS, and Asymmetry. Extracted from 18 nodes per electrode.
Amezquita-Sanchez *et al.* [359]	2019	MCI vs. AD (37 MCI, 37 AD)	Centro Neurolesi Bonino-Pulejo (IRCCS), Messina, Italy	EEG (19-channel resting-state, Eyes Closed)	Enhanced Probabilistic Neural Network (EPNN)	100 iterations of 70% Train / 30% Test split	MCI vs. AD: 90.3% Accuracy (92.1% Sens., 87.9% Spec.)	non-linear Chaos Indices: Fractal Dimension (FD): Three specific measures investigated: Box Dimension (BD), Higuchi's FD (HFD), and Katz's FD (KFD). Persistence Measure: Hurst Exponent (HE) to quantify signal self-similarity. Features were calculated across 20 frequency bands adaptive to the signal content.

Continued on next page

Table S2 – *continued from previous page*

Reference	Year	Comparison Groups	Database	Data Type	Best Classifier	Validation	Best Result	Features Used
Oltu *et al.* [516]	2021	AD vs. MCI vs. HC (8 AD, 16 MCI, 11 HC)	Baskent University Hospital Neurology Dept., Ankara, Turkey	EEG (19-channel resting-state, Bipolar montage)	Bagged Trees	5-fold Cross-Validation; random 50/50 Train-Test split	AD vs. MCI vs. HC: 96.5% Accuracy; AUC 0.99	277 Total Features: PSD Features (228): Variance and Amplitude Summation of the Power Spectral Density (PSD) for 6 sub-bands across all 19 channels. Connectivity Features (48): Amplitude Summation of interhemispheric coherence values for 8 specific channel pairs (e.g., Fp1-F3 vs. Fp2-F4) across the 6 sub-bands. Calculated using the Burg Autoregressive method.
Safi and Safi [540]	2021	NS vs. AD1 vs. AD2 (35 NS, 31 Mild AD, 20 Moderate AD)	Hospital das Clinicas, Sao Paulo, Brazil	EEG (20-channel, Chebyshev type II filtered)	K-Nearest Neighbors (KNN)	Monte Carlo iteration (100 times) with 75% Train / 25% Test	NS vs. AD1 vs. AD2: 97.64% Accuracy (95.40% Sens., 98.81% Spec.)	6 Multi-Domain Features: Statistical Moments: Variance (2nd order), Skewness (3rd order), and Kurtosis (4th order). Entropy Measures: Shannon Entropy (average information/uncertainty) and Sure Entropy. Hjorth Parameters: Activity (signal power), Mobility (mean frequency), and Complexity (bandwidth/ spectral STD).

Continued on next page

Table S2 – *continued from previous page*

Reference	Year	Comparison Groups	Database	Data Type	Best Classifier	Validation	Best Result	Features Used
Cejnek *et al.* [380]	2021	AD vs. MCI vs. CN (59 AD, 7 MCI, 102 CN)	University Hospital Hradec Králové, Czech Republic	EEG (21-channel)	Linear Neuron (ELBND method)	Exhaustive leave-p-out (p=3) Cross-Validation	AD vs. CN: 90.29% Accuracy (89.51% Sens., 90.73% Spec.) at channel T6; MCI vs. CN: 91.62% Accuracy at channel T6	Adaptive Novelty Descriptors: Coefficients (c(k)): Derived from the absolute product of the LNU prediction error e(k) and adaptive weight increments Δw(k). Segment Descriptor: The Standard Deviation of the novelty coefficients across the entire EEG segment (1000 samples). Parameters: History size n=6, regularization term ϵ=1.
Puri *et al.* [526]	2022	AD vs. NC (12 AD, 11 NC)	AFAVA Association, Univ. of Valladolid, Spain	EEG (16-channel)	LS-SVM (RBF Kernel)	10-fold Cross-Validation	AD vs. NC: 92.90% Accuracy; AUC 0.989 (using IMF-4)	10 Statistical & Morphological Features: Statistical: Mean, Variance, Kurtosis, Energy, Maximum, Minimum, and Interquartile Range (IQR). Temporal Complexity: Hjorth parameters (Activity, Mobility, and Complexity). Extracted from 9 Intrinsic Mode Functions (IMFs) generated via Empirical Mode Decomposition.

Continued on next page

Table S2 – *continued from previous page*

Reference	Year	Comparison Groups	Database	Data Type	Best Classifier	Validation	Best Result	Features Used
Amini *et al.* [360]	2021	MCI vs. AD vs. HC (64 MCI, 64 AD, 64 HC)	Dataset from Fiscon *et al.*	EEG (multichannel, Eyes Closed)	Convolutional Neural Network (CNN)	Random split: 80% Train / 20% Validation	MCI vs. AD vs. HC: 82.3% Accuracy; AUC 0.988	7 Time-Dependent Power Spectrum Descriptors (TD-PSD): Moments: Root squared 0-order (m0 - overall power), 2nd-order (m2), and 4th-order (m4) moments. Spectral Shape: Sparseness (energy concentration), Irregularity Factor (IF - ratio of zero crossings to peaks), and Covariance (COV). Energy Operator: Teager Energy Operator (TEO) to detect instantaneous amplitude changes.
Rodrigues *et al.* [536]	2021	C vs. MCI vs. ADM vs. ADA (11 C, 8 MCI, 11 ADM, 8 ADA)	University Hospital Center of São João (UHCSJ), Porto, Portugal	EEG (19-channel, International 10-20)	Artificial Neural Network (Feed-forward MLP)	Leave-one-out Cross-Validation	C vs. MCI: 98.06% Acc.; AUC 0.989. C vs. ADM: 95.99% Acc.; AUC 0.963. C vs. ADA: 94.74% Acc.; AUC 0.961. MCI vs. ADM: 88.84% Acc. MCI vs. ADA: 98.63% Acc.; AUC 0.990. All vs. All: 95.55% Acc.; AUC 0.947.	Lacstral & Cepstral Distance Measures: Distances: Six specific distance types calculated between EEG sub-band pairs (δ,θ,α,β,γ): D1 (Normalized RMS), D2 (RMS without 1st component), D3 (Euclidean), D4 (Quefrency weighted), D5 (Quefrency rooted weighted), and D6 (Quefrency squared weighted).

Continued on next page

Table S2 – *continued from previous page*

Reference	Year	Comparison Groups	Database	Data Type	Best Classifier	Validation	Best Result	Features Used
Houmani *et al.* [437]	2018	SCI vs. MCI vs. AD vs. Other (22 SCI, 58 MCI, 49 AD, 40 Other)	Charles-Foix Hospital, Ivry-sur-Seine, France	EEG (30-electrode)	Polynomial SVM	Leave-one-subject-out Cross-Validation	SCI vs. AD: 91.6% Accuracy. 3-class (SCI/AD/Other): 81.8% to 88.8% Acc. 4-class: SCI (81.8%), AD (89.8%), MCI (60.3%), Other (45%).	Synchrony & Complexity Features: Epoch-based Entropy (EpEn): A complexity measure calculated on piecewise stationary epochs using HMM and Gaussian Mixture Models. Bump Models: Measures of local synchrony capturing 5 parameters per oscillatory event: Position (time/frequency), Width (time/frequency), and Amplitude.
Puri *et al.* [527]	2023	AD vs. MCI vs. NC (59 AD, 7 MCI, 102 NC)	Publicly available dataset (same as Cejnek *et al.*)	EEG (21-channel digital, 128Hz sampling)	Ensemble Bagged Tree (EBT)	10-fold Cross-Validation	AD vs. NC: 97.70% Acc. AD vs. MCI: 94.70% Acc. MCI vs. NC: 94.90% Acc. AD vs. MCI vs. NC: 95.20% Acc.	Multiscale Permutation Entropy (MPE): Information Metrics: Shannon Permutation Entropy (SPE), Tsalli's PE (TPE), and Renyi PE (RPE). Features were extracted from each IMF after dual decomposition (DWT followed by VMD). Parameters: SPE (τ=1,m=6), RPE (m=5,a=2,τ=1), TPE (m=5,q=0.1,τ=1).

Continued on next page

Table S2 – *continued from previous page*

Reference	Year	Comparison Groups	Database	Data Type	Best Classifier	Validation	Best Result	Features Used
Khare *et al.* [461]	2023	AD vs. NC (12 AD, 11 NC)	Alzheimer's Patients' Relatives Association of Valladolid	EEG (16-channel)	XBM	10-fold CV; Holdout (90/10 split); LOSO	AD vs. NC: 99.85% Acc	85 Features per channel (Total 1360): Extracted from 7 subbands. non-linear (42): Higuchi fractal dimension, Hurst exponent, Hjorth mobility/ complexity, Katz fractal dimension, etc.. Entropy (22): Log energy, Approximate, Permutation, Renyi, Shannon, Fuzzy entropy, etc.. Statistical (21): Variance, Mean Absolute Deviation (MAD), Skewness, Kurtosis, Interquartile range, etc.. Significant Features: Univariate analysis identified the top 9 features for channel F3: MVTSR (Mean value of square root), EWL (Enhanced Wavelength), LTKEO, NZR, SpecEN, and SurEN.

Continued on next page

Table S2 – *continued from previous page*

Reference	Year	Comparison Groups	Database	Data Type	Best Classifier	Validation	Best Result	Features Used
Nour *et al.* [514]	2023	AD vs. HC (104 AD, 36 HC combined from two datasets)	Florida State University EEG database	EEG (19-channel)	DEL Model	5-fold CV (80/ 20 train/test split),	AD vs. HC: 97.9% ± 1.1% Acc (AUC: 0.98)	Raw Signal Epochs: No manual feature extraction used. Raw EEG signal segments (1s epochs) are converted into 128x19 2D arrays. Features are automatically learned and extracted through the convolution layers of the internal 2D-CNN models within the deep ensemble framework.
Puri *et al.* [528]	2023	AD vs. MCI vs. HC (I: 12 AD, 11 HC; II: 59 AD, 7 MCI, 102 HC)	Dataset-I: AFAVA; Dataset-II: CUPCRD	EEG (16 and 21-channel)	SVM	10-fold CV; LOSO (80/20 split),	AD vs. HC: 98.90% Acc; AD vs. MCI vs. HC: 96.50% Acc	20 Initial Features: Extracted from 6 decomposed rhythms (delta, theta, alpha, beta1, beta2, gamma). 12 Selected Significant Features (Kruskal-Wallis, $p < 0.001$): Higuchi's fractal dimension (Rank 1), Renyi entropy, Hjorth complexity, Karl's fractal dimension, Band Power Delta, Tsallis entropy, Shannon entropy, Hjorth mobility, Kroskav entropy, Log Root Sum of Sequential Variation, RMS, and Hjorth Activity.

Continued on next page

Table S2 – *continued from previous page*

**Reference**	**Year**	**Comparison Groups**	**Database**	**Data Type**	**Best Classifier**	**Validation**	**Best Result**	**Features Used**
Rostamikia *et al.* [537]	2024	AD vs. FTD vs. HC (36 AD, 23 FTD, 29 HC)	AHEPA University Hospital	EEG (19-channel)	SVM	10-fold CV (70/30 train/test split),	Dementia (AD+FTD) vs. HC: 93.5% Acc; AD vs. FTD: 87.8% Acc	4 Groups of Features: Time-domain: Mean and Variance. Frequency-domain: Power for 5 subbands (delta, theta, alpha, beta, gamma) and Total Power. Complex: Katz Fractal Dimension (KFD), Lyapunov Exponents (LE), and Approximate Entropy (ApEn). Connectivity: Cross-correlation (171 features per subject) and Coherence across all subbands. Selection: Significant differences identified in Cz/Pz (dementia) and Fp1 (AD vs. FTD differential diagnosis).

Continued on next page

Table S2 – *continued from previous page*

Reference	Year	Comparison Groups	Database	Data Type	Best Classifier	Validation	Best Result	Features Used
Siuly *et al.* [557]	2024	AD1 vs. AD2 vs. NC (31 mild, 20 moderate, 35 NC); MCI vs. HC (11 MCI, 16 HC)	Hospital das Clínicas (AD); Unnamed (MCI)	EEG (20 and 19-channel)	LSTM	75% Training / 25% Testing	NC vs. AD1 vs. AD2: 97.00% Acc; MCI vs. Healthy: >99% Acc	Sequential Features (20x16 matrix per channel): Calculated from 5 rhythms (delta, theta, alpha, beta, gamma) divided into 16 windows. Statistical: Mean and Median. Spectral: Lomb-Scargle periodogram Power Spectral Density (LPSD) and Welch's PSD (WPSD). AD Biomarkers: Gamma and Beta rhythms in channels Cz, F4, P4, T6, Pz. MCI Biomarkers: Gamma and Delta rhythms in C3, Fp1, Fz, F8, Cz.
Tzimourta *et al.* [575]	2019	AD vs. Control (8 mild AD, 6 moderate AD, 10 Controls)	AHEPA General Hospital	EEG (19-channel)	Random Forests	10-fold CV	CN vs. Moderate AD: 96.76% Acc; CN vs. Mild AD: 91.77% Acc; Mild vs. Moderate: 91.71% Acc; CN vs. AD: 91.80% Acc	38 Features per channel: Time-based (8): Shannon entropy, Multiscale entropy (MSE), Mean, Variance, Standard deviation, Skewness, Kurtosis, and IQR. Spectral (30): Energy, Relative Band Power (RBP), Approximate entropy, Permutation entropy, Tsallis entropy, and Sample entropy—each calculated for five rhythms (δ, θ, α, β, γ).

Continued on next page

Table S2 – *continued from previous page*

Reference	Year	Comparison Groups	Database	Data Type	Best Classifier	Validation	Best Result	Features Used
Kayasandik *et al.* [459]	2022	TMS+ vs. TMS- (8 improved, 6 declined MMSE responders)	Istanbul Medipol University	EEG (32-channel)	SVM	LOOCV with Monte Carlo technique	Post-TMS (EEG2): 93% Acc; Pre-TMS (EEG1): 79% Acc	Average Power Spectral Density (PSD): Extracted for 5 bands (delta, theta, alpha, beta, gamma) using FFT across 2s non-overlapping windows. Selection Comparison: Evaluated PCA (20 informative features), Wavelet Transform (top 10% largest coefficients), and SWLDA weights. Significant Biomarker: Theta band features were identified as the most informative post-TMS (EEG2) to distinguish responders.

Continued on next page

Table S2 – *continued from previous page*

Reference	Year	Comparison Groups	Database	Data Type	Best Classifier	Validation	Best Result	Features Used
Fan *et al.* [404]	2018	AD severity vs. HC (15 HC, 15 AD1, 69 AD2, 24 AD3)	Taipei Veterans General Hospital	EEG (19-channel)	LASSO	Leave-one-subject-out cross-validation	AD1 vs. AD3: 82.05% Acc (0.87 AUC); HC vs. AD3: 79.49% Acc (0.83 AUC); AD2 vs. AD3: 72.40% Acc (0.71 AUC)	Multiscale Entropy (MSE): Complexity measure of time series signals across 20 time scale factors for 19 EEG channels (Total 380 features). Parameters: pattern length m=2, similarity criterion r=0.15. Selection: LASSO and Elastic Net identified consistent biomarkers in the right temporal, occipital, and parietal regions, particularly at short-term (scales 1-4) and long-term complexity scales.

Continued on next page

Table S2 – *continued from previous page*

Reference	Year	Comparison Groups	Database	Data Type	Best Classifier	Validation	Best Result	Features Used
Ding *et al.* [395]	2022	AD vs. MCI vs. NC (72 AD, 116 MCI, 113 NC)	Tongji Hospital Memory Clinic	EEG (60-channel)	RUS Boosting (Acc) / RF (AUC)	5-fold cross-validation; Permutation tests	AD vs. NC: 72.43% Acc (80.08% AUC); AD vs. MCI: 69.11% Acc (70.82% AUC); MCI vs. NC: 59.91% Acc (63.95% AUC)	Multi-category Feature Set: Spectral: 6 Band Power Ratios (e.g., theta/(alpha+beta1)) and CWT coefficients (Bump mother wavelet). Complexity: Multi-scale (1-20) Permutation, Sample, and Wavelet entropies, plus Lempel-Ziv (LZ) complexity. Connectivity: Correlation and cross-power spectral density correlation coefficients. Dimensionality Reduction: PCA applied to retain latent components representing 95% variability.

Continued on next page

Table S2 – *continued from previous page*

Reference	Year	Comparison Groups	Database	Data Type	Best Classifier	Validation	Best Result	Features Used
Hachamnia *et al.* [426]	2025	AD vs. FTD vs. CN (36 AD, 23 FTD, 29 CN)	Public (OpenNeuro ds004504)	EEG (Resting state, eyes closed)	LGBM	Monte Carlo CV (80:20 train/test split)	AD vs. CN: 95.64% Acc, 99.15% AUC, 94.81% Sens; FTD vs. CN: 96.89% Acc, 99.55% AUC, 96.34% Sens; AD vs. FTD: 95.04% Acc, 98.91% AUC, 91.09% Sens; AD/FTD/CN (3-class): 93.21% Acc.	Time Domain: Hjorth parameters (Mobility, Activity, Complexity), Autocorrelation (lagged values in [-1,1]), and Component Analysis (PCA/ICA eigenvectors). Frequency Domain: Spectral Entropy (Shannon entropy of power spectrum), Relative Band Power (delta, theta, alpha, beta, gamma), FFT coefficients, and Daubechies Wavelet Transform (DWT) coefficients.
Acharya *et al.* [342]	2025	AD vs. FD vs. Control (36 AD, 23 FD, 29 Control)	Public (OpenNeuro ds004504)	EEG (converted to power spectrogram images)	EEGConvNeXt	Hold-out strategy (80:20 split)	AD vs. FD vs. HC: 95.70% Acc, 96.08% Recall; AD vs. FD: 98.21% Acc, 98.17% Recall; AD vs. HC: 96.32% Acc, 96.44% Recall; FD vs. HC: 98.67% Acc, 98.64% Recall.	EEG Power Spectrogram Images: RGB image tensors resized to 224x224x3 capturing time-frequency patterns. Key discriminative features identified via Grad-CAM heatmaps include reduced alpha power, lower temporal stability, and increased theta band kurtosis (3.8).

Continued on next page

Table S2 – *continued from previous page*

Reference	Year	Comparison Groups	Database	Data Type	Best Classifier	Validation	Best Result	Features Used
Goerttler *et al.* [420]	2023	Dataset II: AD vs. CN (36 AD, 29 CN)	Public (OpenNeuro ds004504)	EEG (routine)	SVM (with spatial-spectral balance)	10-fold CV (patient-wise)	AD vs. CN (Dataset I): ∼82% Accuracy; AD vs. CN (Dataset II): ∼80% Accuracy (achieved by balancing spatial and spectral information).	3D Feature Tensors: Dimension configurations balancing resolution across Spectral (PSDs), Temporal (group averaging across segments), and Spatial (graph pooling via spectral clustering on functional connectivity) dimensions. Total features kept constant at 60 (Dataset I) or 180 (Dataset II).
Nasr Mohammed [507]	2024	AD vs. FTD vs. Healthy (36 AD, 23 FTD, 29 CN)	Public (OpenNeuro ds004504)	EEG (Resting state, eyes closed)	LightGBM	Train/Test split (70:30)	AD vs. Healthy: 98.64% Acc, 1.0 AUC, 0.99 Recall; AD vs. FTD: 98.64% Acc, 1.0 AUC, 0.99 Recall; 3-class (AD/FTD/HC): 98.9% Accuracy.	Mel Frequency Cepstral Coefficients (MFCC): 228 features encoding the shape and energy distribution across frequency bands, effectively capturing characteristics originally modeled for speech/audio.
Jang *et al.* [446]	2024	SCD vs. AD (20 SCD, 10 AD)	Korea University Medicine	EEG (Word Recognition Task)	Linear SVM	Train/Val/Test (3:1:1 ratio) split; averaged 50 trials	SCD vs. AD (Old Words): 86% Accuracy, 91% Sensitivity, 76% Specificity; SCD vs. AD (New Words): 57% Accuracy, 73% Sensitivity.	Time-Frequency Power: Total of 144 features derived from 6 channel groups, 6 frequency bands (delta 2-4Hz to low gamma 30-50Hz), and four 500ms post-stimulus windows. Metrics separated by Old Words (OW) and New Words (NW) events.

Continued on next page

Table S2 – *continued from previous page*

Reference	Year	Comparison Groups	Database	Data Type	Best Classifier	Validation	Best Result	Features Used
Tavares *et al.* [568]	2019	AD vs. HS (19 AD, 17 HS)	FMUSP-HC, Univ. São Paulo	EEG (resting-state, 32-channel)	SVM (best trade-off FS/ accuracy)	Leave-one-subject-out (LOSO) CV	AD vs. HS: (LR and Ada Boost) 97.14% Accuracy, 98.55% F1 score; (SVM) 95.43% Accuracy, 97.65% F1 score (after feature/ channel reduction).	Relative Power Spectral Density (%PSD): Matrix of 224 features (32 electrodes x 7 bands). Bands include delta (1.5-6Hz), theta (6.5-8Hz), alpha1, alpha2, beta1, beta2, and beta3 (21.5-30Hz). Selection reduced features to an average of 68.5.
Gopu *et al.* [422]	2024	AD vs. MCI vs. HC (109 subjects; AD/ MCI suspected: 86, HC: 23)	suspected dementia clinic	EEG (multi-channel, Resting state)	KNN	10-fold CV (80:20 split)	AD vs. MCI: 100% Acc (KNN/ANN, No PCA); AD vs. HC: 86.2% Acc (KNN/LSVM/SVMK, PCA); HC vs. MCI: 88.9% Acc (Tree, No PCA); AD/MCI/HC (3-class): 98.9% Acc (PCA).	IMF Facets: Phase and instantaneous amplitude/ frequency information extracted from 5 Intrinsic Mode Functions (IMFs) per channel. Dimensionality reduced to 39 components via PCA while retaining 95% variance.
Hemalatha *et al.* [430]	2026	AD vs. CN (36 AD, 29 Cognitive Normal)	OpenNeuro (Thessaloniki)	EEG (19 channels)	FF-CLSTM (Feature Fusion Conv-LSTM)	60% Train / 20% Test / 20% Validation split	99.8% Accuracy	Spectral: Power Spectral Density (PSD), Spectral Edge Frequency (SEF), Peak frequency, and Spectral Entropy. Moments: Hjorth second- and fourth-order moments (n0, n2, n4). CNN Features: High-level spatial-temporal patterns extracted automatically from deep convolutional layers.

Continued on next page

Table S2 – *continued from previous page*

Reference	Year	Comparison Groups	Database	Data Type	Best Classifier	Validation	Best Result	Features Used
Jiao *et al.* [451]	2023	AD vs. MCI vs. HC (330 AD, 189 MCI, 246 HC) + subtypes (47 FTD, 57 VCI, 21 DLB)	Xiangya Hospital, Central South University	EEG (16-channel resting-state)	LDA (Binary) & SVM (3-way)	Hold-out with internal CV: 80% training set, 20% testing set. Within training set: 5-fold cross-validation (80/20 train/validation split per fold).	HC vs. AD: 85.8% Acc. (LDA); HC vs. MCI: 79.8% Acc. (LDA); 3-way: 70.2% Acc. (SVM).	178 Total Features: Absolute and relative PSD (delta, theta, alpha, beta, gamma); Hjorth Parameters: Activity, mobility, and complexity; Dynamics: STFT time-frequency properties and sample entropy; Microstates: Lifetime, occurrence rate, and converting rate.
Dottori *et al.* [397]	2017	bvFTD vs. AD vs. HC (13 bvFTD, 13 AD, 25 bvFTD-HC, 18 AD-HC)	Multicenter project (Argentina, Chile, etc.)	EEG (128-channel resting-state)	SVM (Combined NPV + CNV)	Hold-out with Repetitions: Training on subset of 7 randomly selected subjects per group; procedure repeated 1,000 times.	AD vs. HC: 88.0% Acc., 0.77 AUC; bvFTD vs. HC: 87.4% Acc., 0.78 AUC; bvFTD vs. AD: 72.9% Acc., 0.70 AUC.	Connectivity (CNV): Weighted Symbolic Mutual Information (wSMI) index in alpha/beta bands across specific fronto-parietal and fronto-temporal ROIs. Neuropsychological (NPV): ACE global score, RAVLT immediate/delayed recall, and IFS total/subtest scores (abstraction, verbal fluency, inhibitory control).

Continued on next page

Table S2 – *continued from previous page*

Reference	Year	Comparison Groups	Database	Data Type	Best Classifier	Validation	Best Result	Features Used
Siuly *et al.* [556]	2020	MCI vs. HC (11 MCI, 16 HC)	Cardiac catheterization units, Isfahan, Iran	EEG (19-channel resting-state)	Extreme Learning Machine (ELM)	10-fold subject-wise cross-validation (repeated 10 times and averaged). Also validated via Leave-one-out cross-validation (LOOCV).	MCI vs. HC: 98.78% Acc., 0.9798 AUC.	Time Dynamics: 4th order Auto-regressive (AR) model coefficients. Complexity: Permutation Entropy (PE) and a 6-bin histogram representing the order distribution patterns of the signal.
Klepl *et al.* [468]	2023	AD vs. HC (20 AD, 20 HC)	Sheffield Teaching Hospital memory clinic	EEG (resting-state, 23 bipolar channels)	AGGCN	Repeated (30x) 10-fold stratified group cross-validation (one group = subject identifier); 8 folds train, 1 validation, 1 test.	AD vs. HC: 89.1% Acc., 0.895 AUC (Eyes-Closed state).	Adaptive Graph: Power Spectral Density (PSD) from 1 to 45 Hz (1 Hz increments) used as node features. Connectivity: Dynamically learned brain graph structure based on Pearson correlation similarity between the enhanced PSD node features.
Carbonaro *et al.* [379]	2022	AD vs. MCI vs. HC (18 AD, 18 MCI, 18 HC)	Greek Association of AD and Related Disorders	EEG (eyes-closed resting, 17 channels)	Conv-LSTM	5-fold cross-validation (by segment split) and 6-fold cross-validation (by subject split: 45 subjects train, 9 subjects validation).	3-way: 99.0% Acc. (Segment), 67% (Subject); AD vs. HC: 95.6% Acc. (Segment), 83.4% (Subject); MCI vs. HC: 92.7% Acc. (Segment), 80.5% (Subject); AD vs. MCI: 94.1% Acc. (Segment), 80.5% (Subject).	Frequency Domain: Raw Fast Fourier Transform (FFT) coefficients resulting in 1000 data points per channel per epoch. Input Format: 3D tensors shaped (epochs x 1000 spectral points x 17 EEG channels).

Continued on next page

Table S2 – *continued from previous page*

Reference	Year	Comparison Groups	Database	Data Type	Best Classifier	Validation	Best Result	Features Used
Adebisi *et al.* [343]	2024	AD vs. MCI vs. VD vs. NC (16 AD, 14 MCI, 10 VD, 10 NC)	Kyungpook National University Hospital, South Korea	EEG (eyes-open resting, 23 channels)	GCN-net (Delta band)	Hold-out: Trials divided into 80% (data from 40 subjects) for training, 10% (5 subjects) validation, and 10% (5 subjects) testing.	NC vs. MCI vs. AD vs. VD: 95.07% Acc., 0.92 F1 (Delta band).	Complex Network (BFNs): Subject-specific brain functional networks based on Phase Lag Index (PLI). Topological Metrics: Rich-club, transitivity, and assortativity coefficients. Information Theory: Statistical and differential entropy features extracted across sub-bands.
Klepl *et al.* [467]	2022	AD vs. HC (20 AD, 20 HC)	Sheffield Teaching Hospital memory clinic	EEG (resting, 23 bipolar channels)	GNN (AEC)	Repeated (50x) 20-fold cross-validation (subject-wise split). 5-fold CV used for initial hyper-parameter search.	AD vs. HC: 92.0% Acc., 0.984 AUC (Alpha band).	Multi-modal Graph: Node Features: Power Spectral Density (PSD) from 0 to 100 Hz. Edges: Adjacency matrices from 8 FC measures (Correlation, Coherence, iCOH, PLI, wPLI, PLV, MI, and Amplitude Envelope Correlation (AEC)).
Mehraram *et al.* [502]	2020	AD vs. DLB vs. PDD vs. HC (32 AD, 25 DLB, 21 PDD, 18 HC)	Newcastle University memory clinic cohort	EEG (128-electrode resting-state)	Random Forest	Six-fold cross-validation with ten repetitions.	LBDs (DLB+PDD) vs. HC: 76% Acc., 0.82 AUC (Beta band); DLB vs. AD: 66% Acc., 0.78 AUC (Beta/Theta bands).	Weighted Graph Measures: WPLI matrices in theta, alpha, and beta bands. Graph Theory: Weighted Node Degree (K), Clustering Coefficient (C), Characteristic Path Length (L), Small-worldness (σ), and Modularity (Q).

Continued on next page

Table S2 – *continued from previous page*

Reference	Year	Comparison Groups	Database	Data Type	Best Classifier	Validation	Best Result	Features Used
Shan *et al.* [548]	2022	AD vs. HC (19 AD, 20 HC)	Sheffield Teaching Hospital memory clinic	EEG (resting-state, 23 bipolar channels)	ST-GCN (WC-based)	Hold-out with 10-fold CV on Training: Two-thirds data used for 10-fold cross-validation (training/ validation); remaining one-third used as independent testing set.	AD vs. HC: 92.3% Acc. (EC state, Full band, Wavelet Coherence adjacency).	Spatial-Temporal: Node Features: Raw voltage values of EEG mini-epochs. Adjacency: Topological coupling matrices derived from Pearson Correlation (PC), MSC, IPC, Wavelet Coherence (WC), PLV, and PLI.
Babiloni *et al.* [365]	2018	ADMCI vs. DLBMCI vs. Nold (30 ADMCI, 23 DLBMCI, 30 Nold)	European PDWAVES and DLB Consortia archive	EEG (resting-state eyes-closed)	ROC analysis (specific markers)	N/A (ROC Analysis): Pairwise binary blind classifications using area under the ROC curve.	Nold vs. ADMCI: 81.7% Acc., 0.86 AUC (Occipital alpha 2); Nold vs. DLBMCI: 81.3% Acc., 0.89 AUC (Parietal delta); ADMCI vs. DLBMCI: 72.5% Acc., 0.72 AUC (Parietal delta).	Cortical Sources: Regional normalized current density (eLORETA) in 8 sub-bands (Delta, Theta, Alpha 1–3, Beta 1–2, Gamma). Spatial: Source density calculated in 6 ROIs (Frontal, Central, Parietal, Temporal, Occipital, Limbic).

Continued on next page

Table S2 – *continued from previous page*

Reference	Year	Comparison Groups	Database	Data Type	Best Classifier	Validation	Best Result	Features Used
Rodrigues & Rodrigues [390]	2025	C vs. MCI vs. Moderate AD (7 MCI, 7 AD, 7 Controls balanced set)	Cejnek *et al.* / Figshare	EEG	ExtraTrees (C vs. MCI); Gradient-Boosting (C vs. AD)	7-fold cross-validation	100% Accuracy (C vs. MCI and C vs. AD); 92.86% (AD vs. MCI)	Time-Frequency Statistics: 8,170 total features resulting from 19 EEG channels × 43 metrics × 10 statistical compressors. Metrics include: 6 entropy types (Sample, Permutation, Approximate, etc.), RMS, Skewness, Kurtosis, 5 PSD bands (δ, θ, α, β, γ), Hjorth complexity, and Katz/Higuchi fractal dimensions. Compressors include mean, median, SD, and 25th-95th quantiles.
Bairagi *et al.* [367]	2018	AD vs. HC (50 AD, 50 Normal)	SKN Medical College and General Hospital	EEG (multichannel)	SVM	Hold-out: 2/3 data samples used for training, 1/3 data samples for testing (50 patients train, 50 patients test).	AD vs. HC: 94% Acc. (96% Sens., 92% Spec.).	Spectral/Wavelet: EEG Relative Power (RP) in sub-bands (D, H, A, B); Mean and variance of 5th level discrete wavelet coefficients. Novel Tool: Transient oscillation detection via frequency bump modelling.

Continued on next page

Table S2 – *continued from previous page*

Reference	Year	Comparison Groups	Database	Data Type	Best Classifier	Validation	Best Result	Features Used
Farina *et al.* [406]	2020	AD vs. aMCI vs. HC (60 AD, 64 aMCI, 65 HC)	Dokuz Eylul University Outpatient Memory Clinic	EEG, MRI	sMRI Model	5-fold CV (80:20); repeated 100x	AD vs. HC: 1.00 AUC (MRI); 0.76 AUC (EEG Rel. Power EC); aMCI vs. HC: 0.73 AUC (MRI); 0.61 AUC (EEG Abs. Power EO); AD vs. aMCI: 1.00 AUC (MRI); 0.67 AUC (EEG Rel. Power EO).	EEG Features: Absolute/Relative power across 7 bands (delta 1-4Hz through gamma 30-45Hz), power ratios (theta/gamma, alpha2/alpha1), global theta, and Weighted Phase Lag Index (WPLI) connectivity for 435 channel pairs. sMRI Features: Grey matter (GM) density vectors consisting of ∼2735 voxels in 3D space. Cognitive: MMSE scores.
Yu *et al.* [601]	2024	AD vs. non-AD (63 AD, 80 non-AD)	CMUH (Precision Medicine Project)	EEG, genetic (genotypes, PRSs)	SVM	5-fold CV; Test set (8:2 ratio)	AD vs. non-AD: (Genetic features) Accuracy: 0.930, AUC: 0.931, Sensitivity: 0.940, Specificity: 0.922; (multi-modal) Accuracy: 0.921, AUC: 0.916, Sensitivity: 0.880, Specificity: 0.951.	EEG (1121 total): 95 Absolute Power, 152 Relative Power, 19 Sample Entropy, and 855 Functional Connectivity (Phase Locking Values - PLV) across 5 bands. Genetic (2690 total): 16 SNPs on Chromosome 19 (including rs429358 in APOE) and 148 PRSs (categorized by traits like brain/gray matter volume and LDL cholesterol).
**Studies with MEG**								

Continued on next page

Table S2 – *continued from previous page*

Reference	Year	Comparison Groups	Database	Data Type	Best Classifier	Validation	Best Result	Features Used
Vaghari *et al.* [576]	2022	MCI vs. Control (158 MCI, 166 Controls)	BioFIND	MEG (resting-state); MRI	SVM	10-fold CV (100 permutations)	67.8% Balanced Accuracy (No covariates)	Sensor-level: Normalised power in Alpha (8-12 Hz) and Beta (13-30 Hz) across 102 magnetometers and 204 gradiometers (712 features). Source-level: Power in 38 ROIs (76 features) and source connectivity using AEC (Amplitude Envelope Correlation) between 38 ROIs via Hilbert transform (1406 features).
Kocagoncu *et al.* [469]	2022	Controls vs. Cognitively Frail vs. MCI vs. AD (38 CN, 26 Frail, 15 MCI, 11 AD)	Cam-CAN and local clinics	MEG (simultaneous EEG/ MEG); MRI	N/A (Focus on group marker differentiation)	1000 permutations for cluster statistics	Significant differentiation between clinical (AD/MCI) and non-clinical groups	Neurophysiological: Evoked cross-modal mismatch response (RMS time series 200-500ms) for Novelty (STD-DN) and Associative (STD-DA) contrasts in 6 source ROIs (L/R IFG, STG, HG). Structural: Mean regional Gray Matter Volume (GMV) from bilateral hippocampal and entorhinal ROIs (Harvard-Oxford atlas).

Continued on next page

Table S2 – *continued from previous page*

Reference	Year	Comparison Groups	Database	Data Type	Best Classifier	Validation	Best Result	Features Used
Ahmad *et al.* [347]	2024	MCI vs. HC (158 MCI, 166 HC)	BioFIND	MEG; MRI	GLMNET with sign constraints (LCMV on GRAD)	10-fold nested CV (20 Monte Carlo iterations)	74.57% Accuracy (71.55% sens., 73.29% F1)	Spectral: Relative Power Spectral Density (rPSD) across six bands: δ (2-4Hz), θ (4-8Hz), α (8-12Hz), β (12-35Hz), low γ (30-48Hz), and high γ (52-86Hz). Applied to 612 MAG sensors, 1224 GRAD sensors, or 3000 source dipoles.
Liu *et al.* [492]	2024	MCI vs. HC (144 MCI, 163 HC)	BioFIND	MEG (rsMEG); MRI	InterFusion (Spatial-Channel Cross-Attention)	5-fold cross-validation	82.7% Accuracy (0.785 F1, 0.678 sens., 0.957 spec.)	Structural: 3D brain morphology patches from sMRI (192x192x182 voxels). Functional: Magnetometer (MAG) sensor features (102 x 8192) across 6 frequency bands (delta, theta, alpha, beta, low/high gamma), particularly High-Gamma (52-86Hz).

Continued on next page

Table S2 – *continued from previous page*

Reference	Year	Comparison Groups	Database	Data Type	Best Classifier	Validation	Best Result	Features Used
Giovannetti *et al.* [418]	2021	HC vs. sMCI vs. pMCI (33 HC, 27 sMCI, 27 pMCI)	Hospital Universitario San Carlos, Madrid	MEG (rsMEG); MRI	Deep-MEG Ensemble #1 (binary); Ensemble #2 (3-class)	Leave-one-patient-out (LOPO)	89% Accuracy (sMCI vs. pMCI, pre-conversion); 74% Accuracy (3-class)	Connectivity Maps: Topologically organized 90x90 FC maps of PLV (Phase Locking Value) and MC (Magnitude Coefficient). RGB Images: Combinations of MC_cent_ma/am in β and $\beta 1$ bands and Intra-ROI PLV in $\beta 1$. Deep spatiotemporal features from Conv5 AlexNet layer.
Yang *et al.* [597]	2021	MCI vs. HC (20 MCI, 20 HC)	Hospital Universitario de San Carlos, Madrid	MEG (source-space); MRI	SVM (Euclidean kernel) for LOO; 3-NN for MCR	MCR random split (95/5%) and 20-fold LOO CV	93.83% Mean Acc (MCR); 83.8% Acc (LOO) using hyper-connected ROIs	Connectivity Indicators: Magnitude-squared spectral coherence (MS-COH) spatial indices identifying hyper-connected ROIs (e.g., LF, LT, RC, RT, LP, LO). Wavelet Features: SD (Standard Deviation) of the approximate derivative of wavelet coefficients representing non-stationary multi-resolution properties in 14 frequency ranges.

Continued on next page

Table S2 – *continued from previous page*

Reference	Year	Comparison Groups	Database	Data Type	Best Classifier	Validation	Best Result	Features Used
Lopez-Martin *et al.* [495]	2020	MCI vs. HC (78 MCI, 54 Healthy aging)	Centre for Biomedical Technology (Madrid, Spain)	MEG	Randomized 2D-CNN	Two-stage nested cross-validation (Outer & inner iterations)	0.92 Average F1-score; 0.91 Accuracy	NMI Features (FS2 set): 870 total features extracted from normalized mutual information (NMI) between 102 magnetometers (5,151 sensor pairs). For each sensor pair, 5 summary statistics were computed: mean, median, SD, mean absolute deviation (MAD), and range. FS2 was the intersection of features from Random Forest importance, Boruta, and RFE.
Vaghari *et al.* [577]	2022	MCI vs. HEC (144 MCI, 163 Healthy Elder Controls)	BioFIND	MEG; MRI	Late combination of modality-specific classifiers using EasyMKL	5-fold cross-validation repeated 1000 times	77% Accuracy (multi-modal)	Multi-modal late combination: sMRI (T1): Mean gray-matter volume across 110 ROIs of the Harvard-Oxford Atlas. MEG: Best result used the covariance of planar gradiometer (GRD) data (20,706 features) in the low Gamma range (30–48 Hz). Covariance captures both brain activity and connectivity.

Continued on next page

Table S2 – *continued from previous page*

Reference	Year	Comparison Groups	Database	Data Type	Best Classifier	Validation	Best Result	Features Used
Ikeda *et al.* [443]	2020	Early stage AD vs. NC (20 AD [6 MCI, 14 AD dementia], 27 Normal Controls)	Nakajima study (Japan)	MEG; MRI	SVM	6-fold cross-validation (randomly split into 1700 training/340 test segments)	83% Discrimination Accuracy (70% sens., 93% spec.); 0.91 AUC	MEG/sMRI Source-level features: 2,040 total parameters. 6 values per region: (1) absolute power EC, (2) absolute power EO, (3) WCN power EC, (4) WCN power EO, (5) EC-EO power difference, and (6) WCN value for EC-EO difference. Each value was calculated across 5 frequency bands ($\theta1$, $\theta2$, $\alpha1$, $\alpha2$, β) in 68 regions (Desikan-Killiany atlas).
Pusil *et al.* [529]	2019	Stable MCI vs. Progressive MCI (27 sMCI, 27 pMCI)	Hospital Universitario San Carlos (Madrid, Spain)	MEG; MRI	SVM	5-fold cross-validation repeated 100 times (80% training/20% test split)	100% Accuracy (Multiplex integrated scenario)	Dynamic Connectivity (DTW): 30 multiplex features representing temporal distances (Dynamic Time Warping) between fluctuations of functional coupling strength (Phase Coupling Estimation - PCE) across 90 ROIs (AAL atlas) across two sessions. Calculated for 5 bands (δ, θ, α, β, γ); the γ band performed best individually.

Continued on next page

Table S2 – *continued from previous page*

Reference	Year	Comparison Groups	Database	Data Type	Best Classifier	Validation	Best Result	Features Used
Xu *et al.* [595]	2021	NC vs. sMCI vs. pMCI (53 NC, 48 sMCI, 28 pMCI)	Hospital Universitario San Carlos (Madrid, Spain)	MEG; MRI	MG2G (Multiple Graph Gaussian embedding model)	5-fold cross-validation	79% Acc. (NC vs. sMCI); 78% Acc. (sMCI vs. pMCI); 82% Acc. (NC vs. pMCI)	Stochastic Graph Embeddings: Latent node embeddings represented as multivariate Gaussian distributions in a 16-dimensional latent space. Each of the 68 brain regions (Desikan-Killiany atlas) is represented by a mean vector (node position) and a variance vector (uncertainty). Inputs derived from MI networks in the Alpha band (8-12 Hz).
Javed *et al.* [447]	2024	Cross-sec: SCD vs. MCI vs. HC (85 SCD, 142 MCI, 116 HC). Long: 22 stable vs. 23 progressive	BioFIND dataset (UCM/UH)	MEG (source-reconstructed); MRI	SVM	Hold-out with internal CV: 75% training (seen), 25% testing (unseen). Within training set: Leave-one-out cross-validation.	HC vs. MCI: 86% Acc., 0.91 AUC (SVM); HC vs. SCD: 75% Acc., 0.78 AUC (SVM); SCD vs. MCI: 76% Acc., 0.82 AUC (SVM).	Multi-modal Biomarkers: Long-range temporal correlations (LRTCs) measured by DFA exponents. Dynamics: Functional E/I balance (fE/I) values. Structural Anatomy: Bilateral medial temporal lobe (MTL) volumes extracted from T1-MRI (Hippocampus, Parahippocampus, and Entorhinal cortex).

Continued on next page

Table S2 – *continued from previous page*

Reference	Year	Comparison Groups	Database	Data Type	Best Classifier	Validation	Best Result	Features Used
Samanta *et al.* [543]	2024	MCI vs. Control (5 MCI, 16 Controls)	Ulster University	MEG (source-level); MRI	Graph-BrainConvNet (G-BcN)	Leave-one-subject-out (LOSO)	92.63 ± 0.96% Accuracy (Gamma rhythm, C2 condition)	Connectivity: Phase synchrony (iCOH) weights between 122 source time series. Node Features (17 per source): Peak amplitude, latency, AUC, slope, peak-to-peak amp, mean absolute amp, RMS amp, SD, skewness, kurtosis, ZORK, variance/ skewness/kurtosis of power, maximum power, spectral entropy, and bandpower.
